# Latitude- and depth-driven divergence in protist trophic strategies revealed by a machine learning model

**DOI:** 10.3389/fmicb.2025.1602162

**Published:** 2025-09-01

**Authors:** Elaina Thomas, Mora J. Groussman, Sacha N. Coesel, Nicholas J. Hawco, Randelle M. Bundy, E. Virginia Armbrust

**Affiliations:** ^1^School of Oceanography, University of Washington, Seattle, WA, United States; ^2^Department of Oceanography, University of Hawai'i Mānoa, Honolulu, HI, United States

**Keywords:** protist, marine, North Pacific, mixotrophy, trophic mode, machine learning, plankton

## Abstract

Protists are ubiquitous across the ocean, holding different roles in the food web depending on their trophic capabilities. Many protists are mixotrophs, which are capable of both photosynthesizing and ingesting prey. However, there is limited knowledge of which protist species are mixotrophs in nature, as well as their activity and distribution throughout the ocean. Here, we present Marine PRotist *In Situ* trophic Mode predictor (MarPRISM), a refined XGBoost-based machine learning model for predicting the *in situ* trophic mode (phototrophy, mixotrophy, and heterotrophy) of marine protist species based on transcriptional profiles. We used MarPRISM to generate 1,462 trophic mode predictions for 28 environmental protist species based on 335 metatranscriptomes collected across the North Pacific Ocean, from the surface to 130 m depth, over the diel cycle, and within nutrient-amended incubations. Eight environmental species were identified as having mixotrophic capabilities, including six dinoflagellates, one bolidophyte, and one haptophyte. The species with mixotrophic capabilities varied in how they shifted their trophic mode across the surface ocean and in response to the experimental amendment of nitrate and iron. Limited light availability appeared to lead one species to shift from mixotrophy at the surface toward heterotrophy between 41 and 130 m depth. We used transcript abundance to evaluate the abundance of species with different trophic capabilities (species with mixotrophic capabilities, phototrophic specialists, and heterotrophic specialists). At the surface within the subtropical gyre, transcript abundance was similar among protist species with different trophic capabilities. In the gyre, the protist community was nitrate-limited, and experimental nitrate amendment favored phototrophic specialists. Increasing nitrate availability with latitude was correlated with phototrophic specialists being the dominant protist trophic group in the transition zone between the subtropical and subpolar gyres under high nitrogen availability. In contrast, under lower nitrogen conditions in the transition zone, protist species with different trophic capabilities comprised equal portions of the surface community. Light and nitrate availability influenced the transcript abundance of phototrophic specialists across depth; phototrophic specialists had high transcript abundance at 130 m in the subtropical gyre and at the surface in the transition zone, while species with mixotrophic capabilities and heterotrophic specialists showed less variation in transcript abundance with depth.

## Introduction

Single-celled eukaryotes (protists) are widespread and abundant members of marine planktonic communities. Protist species traditionally assumed to be phototrophic due to their possession of plastids are increasingly recognized as mixotrophs (Flynn et al., 2013), able to rely on both photosynthesis and heterotrophy (a strategy known as mixotrophy) or utilize each mode independently to acquire carbon, nitrogen, phosphorus, and trace elements such as iron and vitamins. Mixotrophic plankton are predicted to enhance primary production, trophic transfer efficiency, and carbon export (Mitra et al., 2014; Ward and Follows, 2016). Further, mixotrophs exert control over lower trophic levels, with mixotrophic flagellates estimated to carry out 40% or more of bacterivory in oligotrophic surface waters (Zubkov and Tarran, 2008).

Mixotrophic protists compete with strictly phototrophic protists for dissolved inorganic nutrients and light, and with strictly heterotrophic protists for prey. The requirement of mixotrophs to allocate biomass and energy among more functions than specialists may lead to greater respiratory demand, lower photosynthetic efficiency than phototrophs, and lower grazing rates than heterotrophs (Raven, 1997; Berge et al., 2017). Mixotrophs are predicted to be favored when the ability to synergistically use light, prey, and nutrients through photosynthesis and phagocytosis overcomes the limitations experienced by specialists. When prey is limiting, mixotrophs can use energy from photosynthesis to fix carbon and thus may outcompete heterotrophic specialists. When nutrient concentrations are limiting, mixotrophs may access nutrients trapped in prey cells to support photosynthesis and potentially outcompete photosynthetic specialists. When sufficient light is available, mixotrophs may use the excess energy generated by photosynthesis for phagocytosis, potentially allowing mixotrophs to suppress prey to densities too low for heterotrophs to efficiently graze (Hsu et al., 2022). The complexity of mixotrophic lineages challenges the application of universal trait trade-offs to functional groups (Mitra et al., 2023b). For example, obligate mixotrophic species must utilize both photosynthesis and phagocytosis to support growth. In contrast, facultative mixotrophic species can grow without prey when provided light, or in the dark when provided ample prey. Even closely related mixotrophic protist species can exhibit different trophic strategies, varying in their allocation of resources to photosynthesis and grazing (Wilken et al., 2019).

The study of mixotrophic protists has evolved significantly over the years. Prey ingestion has traditionally been detected in the lab or at sea using fluorescent prey (naturally fluorescent or labeled) and microscopy (e.g., Stoecker et al., 1997; Connell et al., 2020), or stable isotope probing (e.g., Frias-Lopez et al., 2009; Orsi et al., 2018). These methods classify a cell as behaving mixotrophically when prey ingestion coincides with the presence of a plastid. While informative, these techniques are labor-intensive, limiting their applicability for studying diverse protist species across vast oceanic regions. Mathematical models offer an alternative approach, enabling broader generalizations about mixotrophs across different species and oceanic regions. Pairing a meta-analysis and dynamic model, Edwards (2019) predicted mixotrophic protists to dominate over heterotrophic and phototrophic specialists in well-lit, nutrient-poor gyre surface waters (Edwards, 2019). Models based on optimal resource allocation can predict the optimal trophic behavior of mixotrophic protists without having to define trade-offs *a priori* (Moeller et al., 2024). Using this method, Moeller et al. (2024) identified phagotrophy as the dominant strategy of mixotrophic protists across the oceans, used primarily for nitrogen acquisition. These model results generate testable hypotheses that can be explored with field data.

Recent studies examining the distribution of mixotrophic protists compared to their specialist counterparts (Edwards et al., 2023; Dong et al., 2024; Edwards et al., 2024) use relatively few cultured isolates to infer trophic capabilities of protists in the ocean based on the behavior of their close relatives in culture. An alternative approach is to predict trophic capabilities directly from sequencing data. Burns et al. (2018) laid the groundwork for predicting trophic capabilities based on the presence or absence of specific genes. They developed a gene-based model trained on 35 eukaryotic genomes, using protein clusters to distinguish between phagotrophs and phototrophs. Random Forest models were employed for feature selection, and a neural network classifier was trained to predict trophic capabilities. To account for genome reduction in parasitic phagotrophs, the authors also created specialized models. While transcriptomes risk underestimating genomic potential, the model was validated on 112 genomes and transcriptomes, accurately predicting trophic capabilities across diverse eukaryotic species. The model was subsequently experimentally validated in green algae (Bock et al., 2021), and used to investigate conflicting evidence for phagotrophy by *Micromonas* (Jimenez et al., 2021), predict the potential for phagotrophy in *Braarudosphaera bigelowii* (Suzuki et al., 2021), which was later confirmed (Mak et al., 2024), and predict the mixotrophic capabilities of *Phaeocystis* and other haptophytes (Koppelle et al., 2022).

The application of machine learning beyond individual species and isolates to include meta-omic data offers a promising approach to identify and examine diverse mixotrophic protists in their natural environments without the need for cultivation. Alexander et al. (2023) built a Random Forest model to predict trophic capabilities from marine protist metagenomes and -transcriptomes based on the presence and absence of Kyoto Encyclopedia of Genes and Genomes (KEGG) orthologs (Kanehisa and Goto, 2000). Like the Burns et al. (2018) model, their model predicted species' trophic capabilities based on genomic potential rather than gene expression. The Alexander et al. (2023) model did not identify any species as mixotrophs across the oceans, despite the presence of known mixotrophs in the examined regions. To address the continuum of protist trophic capabilities, Alexander et al. (2023) introduced a heterotrophy index to classify transcriptomes and metagenome-assembled genomes along a gradient from highly phototrophic to highly heterotrophic.

To predict the *in situ* trophic mode rather than the trophic capabilities of marine protist species, Lambert et al. (2022) developed an Extreme Gradient Boosting (XGBoost) model trained on transcriptional profiles rather than genomic content. The Lambert et al. (2022) model was trained on transcriptomes generated through the Marine Microbial Eukaryote Transcriptome Sequencing Project (MMETSP) (Keeling et al., 2014) based on transcriptional patterns grouped at the level of protein families (Pfams). A key feature of the (Lambert et al. 2022) model is its ability to assign multiple trophic modes to a single species under different growth conditions, acknowledging the flexibility of protist metabolism. The model does not assume that different species use the same gene expression strategies when switching between trophic modes, nor that any predefined marker genes will be important for predicting trophic mode. However, the model does assume that protist species regulate their trophic mode through changes in gene expression—that is, that transcriptional shifts underlie transitions between phototrophy, mixotrophy, and heterotrophy. The (Lambert et al. 2022) model enabled predictions of phototrophy, mixotrophy, and heterotrophy for protist species in their natural environment. Recent studies (Lasek-Nesselquist and Johnson, 2019; Van Vlierberghe et al., 2021; Groussman et al., 2023a) have identified entries present in the MMETSP-derived training dataset with low sequence abundance and high rates of contamination, suggesting that the (Lambert et al. 2022) model could be further improved.

We aimed to enhance the model developed by (Lambert et al. 2022) by incorporating recent advancements in machine learning software and addressing the identified issues within the training dataset. We broadened the application of the updated model by analyzing both the *in situ* trophic mode (phototrophy, mixotrophy, heterotrophy) of marine protist species, and by aggregating these trophic mode predictions to predict overall trophic capabilities (species with mixotrophic capabilities, phototrophic specialists, heterotrophic specialists). We paired the model with quantification of transcript abundance to examine the abundance of mixotrophs relative to phototrophic and heterotrophic specialists. We sought to apply the model to additional metatranscriptomes from surface transects, depth profiles (to 130 m), nutrient amendment incubations, and diel experiments collected across the North Pacific subtropical–subpolar transition zone. The gyre waters south of the transition zone enable examination of the composition and trophic mode of protists in an oligotrophic environment dominated by *Prochlorococcus* biomass (Hynes et al., 2024), and serve as a contrast to the more nitrate-rich waters north of the chlorophyll front where net primary and community production are significantly higher (Juranek et al., 2012, 2020) and functionally diverse pico- and nano-eukaryotes are abundant (Juranek et al., 2012; Kavanaugh et al., 2014; Juranek et al., 2020).

## Methods

### Machine learning model development

We trained and tested both Random Forest and XGBoost (Chen and Guestrin, 2016) machine learning models, using Scikit-learn version 1.5.1 and XGBoost version 1.7.4, respectively. Both models were trained with poly(A)-selected marine eukaryotic transcriptomes generated through the MMETSP (Keeling et al., 2014) (Supplementary Figure S1a). As described by Lambert et al. (2022), the assemblies, functional annotations, and mapping results for these transcriptomes were derived from re-assembly efforts (Johnson et al., 2017; Patro et al., 2017; Johnson et al., 2019). Our training dataset included those assemblies from the MMETSP-generated transcriptomes that passed quality thresholds defined by Groussman et al. (2023a): at least 1,200 total sequences, at least 500 total assigned Pfam domains, and < 50% contamination from non-target organisms (percent of ribosomal protein sequences with taxonomic identity other than the recorded identity) (Lasek-Nesselquist and Johnson, 2019; Van Vlierberghe et al., 2021; Groussman et al., 2023a) (Supplementary Figure S1a). Removal of transcriptomes with low sequence abundance or high rates of contamination resulted in a training set of 387 MMETSP-derived transcriptomes that could be assigned a trophic mode label: 258 phototrophic, 85 mixotrophic, and 44 heterotrophic (Supplementary Data Sheet S1). The trophic mode labels are the same as those used by Lambert et al. (2022), but the final set of entries in the new training dataset is reduced. The trophic mode labels assigned to each transcriptome were based on the trophic capabilities of taxa reported in the literature and the specific culture growth conditions: a transcriptome was labeled as mixotrophic if it came from a taxa known to be capable of mixotrophy that was grown in the light with bacteria present, as phototrophic if the same species was grown in the light without bacteria, or as heterotrophic if the same species was grown in the dark with bacteria. Compared to the original Lambert et al. (2022) training dataset, the new cleaned training dataset reduced the number of phototrophic entries by 17, mixotrophic entries by 8, and heterotrophic entries by 34 (Supplementary Data Sheets 1, 2). Input to train the machine learning models consisted of transcripts per million (TPM) values aggregated by Pfam, species, and sample. A machine learning model was also tested based on the TPM values converted to binary. Pfams with zero mapped transcripts in a given sample were assigned a count of zero for that sample (Lambert et al., 2022).

We conducted feature selection on different versions of the training dataset (Supplementary Figure S1a). Because the training dataset was imbalanced with 258 phototrophic, 85 mixotrophic, and 44 heterotrophic entries, feature selection was conducted on four versions of the dataset after removing the contaminated and low-sequence transcriptomes, each with a single random subset of phototrophic transcriptomes of different subsample sizes (number of phototrophic transcriptomes = 50, 80, 100, and 120), along with all mixotrophic and heterotrophic transcriptomes. This was repeated with TPM values converted to binary. We also performed feature selection on four versions of the training dataset in which transcriptomes from *Micromonas* species originally labeled as mixotrophic were reclassified as phototrophic. In each version, a single random subset of phototrophic transcriptomes was selected (number of phototrophic transcriptomes = 50, 80, 100, and 120), while all mixotrophic and heterotrophic transcriptomes were retained. Finally, we ran feature selection on the training dataset containing the transcriptomes with low sequence abundance and high rates of contamination to enable comparison of model performance with and without removal of these transcriptomes from the training dataset; feature selection was conducted on four versions of this training dataset with a single random subset of phototrophic transcriptomes (number of phototrophic transcriptomes = 80, 100, 120, and 140), along with all of the mixotrophic and heterotrophic transcriptomes.

The mean decrease in accuracy method was used to determine the feature Pfams essential for model performance (Supplementary Figure S1a). It was run based on a Random Forest or XGBoost (Chen and Guestrin, 2016) model using the undersampled training datasets with a custom script (https://github.com/armbrustlab/MarPRISM/blob/main/modelDevelopmentTesting/mda.py). The input data was the TPM values of the Pfams scaled between zero and one using Scikit-learn's MinMaxScaler for the majority of models, while one model was tested using TPM values of the Pfams converted to binary. The training dataset (one of the four randomly undersampled versions) was split into training and test sets with 10 splits (iterations) and a test size of 30% randomly selected data using Scikit-learn's ShuffleSplit. The model was fit to the training set, predictions were made for the test set, and the F1 score—the harmonic mean of precision and recall scores—of predictions was calculated. Then, for each Pfam, expression values were randomly shuffled across samples, the model re-predicted the target variable, and the F1 score was recalculated. The importance score was quantified for each Pfam: the difference in the F1 score before and after shuffling, normalized by the original F1 score [(F1 score – F1 score after shuffling)/F1 score]. For each Pfam, the importance scores were averaged across the 10 iterations. For each model, Pfams with a negative mean importance score (a mean decrease in F1 score after shuffling) across iterations for at least one of the four randomly undersampled datasets were retained. Thus, Pfams were selected for the feature set if their inclusion increased model performance by any magnitude.

Grid searches with Scikit-learn's GridSearchCV and 5-fold cross-validation were conducted to select the best-performing hyperparameters for the Random Forest and XGBoost models. A custom script (https://github.com/armbrustlab/MarPRISM/blob/main/modelDevelopmentTesting/parameter_gridsearch.py) was run on a single randomly sampled set of 100 phototrophic transcriptomes, as well as on all mixotrophic and heterotrophic transcriptomes from the previously described training datasets. The following hyperparameters were evaluated for the Random Forest model: n_estimators (10, 100, 1,000, 10,000), max_depth (1, 10, 1,000, None), min_samples_split (2, 5, 10, 20), min_samples_leaf (1, 3, 5, 10), and min_weight_fraction_leaf (0, 0.2, 0.5). For the XGBoost model, the following hyperparameters were tested: n_estimators (10, 100, 1,000), max_depth (3, 10, 20), learning_rate (0.05, 0.1, 0.15, 0.2), gamma (0, 0.5, 1), and reg_lambda (0, 0.5, 1). Treating the Random Forest and XGBoost models separately, the F1 score was calculated for each combination of hyperparameters. The set of hyperparameters with the highest mean F1 score (averaged across 5-fold cross-validation) was selected for each model.

### Performance of different machine learning models

Performance of the Random Forest and XGBoost models was estimated using cross-validation (Supplementary Figure S1a), where models were trained on 83% of the training dataset and tested on the remaining data. For cross-validation, the proportion of transcriptomes of each trophic mode in each training and test set of transcriptomes was preserved using Scikit-learn's StratifiedShuffleSplit. Cross-validation was performed with six splits. Model performance was evaluated with F1 score. Mean F1 score was calculated for each model across the three trophic modes weighted by the number of true instances of each class, and for each trophic mode separately with Scikit-learn. The F1 scores of different models were compared with one-way analysis of variance tests (ANOVAs).

Separation of the phototrophic, mixotrophic, and heterotrophic training dataset transcriptomes was visualized using *t*-distributed stochastic neighbor embedding (*t*-SNE) with the R package Rtsne after scaling the TPM of each Pfam by its mean and standard deviation across samples. The clustering of transcriptomes based on features in *t*-SNE space was quantified with silhouette scoring using the R package cluster; the silhouette score is a measure of the distance in *t*-SNE space of each training dataset transcriptome to transcriptomes in its cluster (trophic mode) compared to the distance to transcriptomes in other clusters (trophic modes). The phototrophic, mixotrophic, and heterotrophic training dataset transcriptomes were also visualized with hierarchical clustering; the expression of each feature Pfam, in TPM, was averaged (median) across the training dataset transcriptomes of each trophic mode, then the trophic modes were hierarchically clustered based on Euclidean distance after scaling the median TPM values. Additionally, the expression of feature and non-feature Pfams in the training dataset transcriptomes was compared with a one-tailed *t*-test.

Transcriptomes not included in the training dataset were used for further testing (Supplementary Data Sheet S3). These transcriptomes originated from cultures of protists grown under varied laboratory conditions (Lie et al., 2017, 2018; McKie-Krisberg et al., 2018; Onyshchenko et al., 2021; Lambert et al., 2022; Graff van Creveld et al., 2023; Charvet et al., 2024). Some of these transcriptomes were processed and used for testing by Lambert et al. (2022), while other transcriptomes, from *Pterosperma cristatum* (Charvet et al., 2024), *Amphora coffeaeformis, Chaetoceros* sp., and *Cylindrotheca closterium* (Graff van Creveld et al., 2023), were newly added as tests in this study. In assigning an expected trophic mode to transcriptomes of *Micromonas polaris*, we considered the species to have mixotrophic capabilities given its inclusion in the Mixoplankton Database (Mitra et al., 2023a); however, we recognize that there is debate regarding the mixotrophic capabilities of this species (Jimenez et al., 2021). We assigned an expected trophic mode of phototrophy or mixotrophy to transcriptomes from *P. cristatum* grown in F/2 media with bacteria and light, as well as in F/20 media with bacteria and light, both sampled on day 11 because prey ingestion was not significantly different from the unfed control in these treatments but there was a low baseline feeding frequency (~6 and ~13% of cells, respectively) (Charvet et al., 2024). In contrast, transcriptomes from *P. cristatum* grown in F/20 with bacteria and light, sampled on day 16 were assigned an expected trophic mode of heterotrophy or mixotrophy, as 60% of cells exhibited feeding, a significant increase compared to the control, and there was a significant reduction or abatement of photosynthetic activity based on transcriptomic analysis (Charvet et al., 2024). *Ochromonas* sp. CCMP1393, grown in the dark with bacteria was assigned an expected trophic mode of heterotrophy or mixotrophy, as chlorophyll content and gene expression measurements indicated that the strain maintained photosynthetic readiness by keeping its enzymatic machinery active. In contrast, *Ochromonas* sp. BG-1 appeared to shut down its photosynthetic capacity under the same conditions and was thus assigned an expected trophic mode of heterotrophy. TPM for the additional transcriptomes were derived from publicly available salmon mappings (Patro et al., 2017). Pfam annotations for these transcriptomes were generated through the following: the publicly available assembled transcriptome for each species was six-frame translated with transeq (version 6.6.0.0) (Rice et al., 2000); the longest reading frame (minimum 100 amino acid length) was selected for each contig; the amino acid sequence of the longest reading frame was compared to the Pfam database (v34.0) (Bateman et al., 2004) with hmmsearch (version 3.3) using “cut_tc” trusted cutoff score (Finn et al., 2011); only Pfam annotations with an e-value less than 1e-05 were retained; the Pfam annotation with the best bitscore for each contig was selected; then, TPM was summed by Pfam. Trophic predictions were generated for these transcriptomes using the new models, which were then compared to the expected trophic mode and trophic predictions made by the earlier iteration of the model (Lambert et al., 2022). The MMETSP entries removed from the training dataset due to low sequence abundance or high rates of contamination were also used for testing of the new models (Supplementary Data Sheet S4).

### Sample and environmental data collection

Samples for metatranscriptomes were collected during four oceanographic research cruises in the northeast Pacific Ocean (Supplementary Figure S1b, Supplementary Data Sheet S5): a diel study (ALOHA diel) conducted (July 2015) ~100 km NE of Station ALOHA (A Long-term Oligotrophic Habitat Assessment) in the North Pacific Subtropical Gyre (22.45°N, 158°W) (Wilson et al., 2017), and three Gradients cruises, each of which followed 158°W. Gradients one (G1) transited from 21.45 to 37.8°N from 20 April 2016 to 04 May 2016; Gradients two (G2) transited from 21.3 to 42.43°N from 26 May 2017 to 13 June 2017; and Gradients three (G3) transited from 21.26 to 42.33°N from 10 April 2019 to 29 April 2019 (Juranek et al., 2020; Groussman et al., 2024c). During the ALOHA diel cruise, samples were collected from 15 m from a single water mass, following a Lagrangian drifter, every 4 h over 4 days. Samples for the surface transects were collected from 15 m during G1 and G2, and from 7 m during G3. The majority of the Gradients surface metatranscriptomic samples were collected at dawn. Three sets of surface water nutrient amendment experiments were conducted during G2 at 32.93, 37, and 41.42°N. The surface water community from 15 m was prefiltered through 100 μm mesh, then grown in 20 L bottles with different quantities of nitrate, phosphate, and dissolved iron added: at 32.93°N, 0.5 μM nitrate + 0.05 μM phosphate (+LoNP), 5 μM nitrate + 0.5 μM phosphate (+HiNP), 0.5 nM iron + 5 μM nitrate + 0.5 μM phosphate (+NPFe); at 37°N, 1 nM iron (+Fe), 5 μM nitrate + 0.5 μM phosphate (+NP), 1 nM iron + 5 μM nitrate + 0.5 μM phosphate (+NPFe); and at 41.42°N, 0.3 nM iron (+LoFe), 2 nM iron (+HiFe), 2 nM iron + 10 μM nitrate + 1 μM phosphate (+NPFe) (Supplementary Data Sheet S6). Samples from these incubations, including from controls (no nutrient amendment), were collected for metatranscriptomes along with chlorophyll *a* measurements after 0 and 96 h. A diel study (G3 diel) was conducted during G3 at ~41.6°N, with samples collected approximately every 4 h for 3 days from 15 m following a Lagrangian drifter. During G3, samples for metatranscriptomes were collected from depth profiles at 32.92, 37, 41.67, and 42.33°N. The mixed layer depth (MLD) was determined from the depth at which potential density was 0.03 kg/m^3^ greater than the potential density at 10 dbar (de Boyer Montégut et al., 2004) using the Gibbs-SeaWater Oceanographic Toolbox (McDougall and Barker, 2011) applied to conductivity, temperature, and depth-rosette (CTD) profiles. The euphotic zone depth was determined as the depth with 1% of surface photosynthetically active radiation (PAR). While we refer to the incubations conducted at 32.93°N during G2 and the depth profile at 32.92°N during G3 as in the gyre, it should be noted that these locations are near the southern boundary of the transition zone in winter.

Measurements of nutrients, PAR, temperature, bacteria and picoeukaryote biomass, and net community production for the G1–G3 surface transects and depth profiles originate from previous studies (Ribalet et al., 2019; Juranek et al., 2020; Pinedo-González et al., 2020; Park et al., 2023; Hawco et al., 2025) and data repositories (Cain et al., 2020a,b,c; Juranek, 2020a,b,c; White, 2020, 2021; Simons CMAP Curator, 2022; NASA Goddard Space Flight Center, Ocean Ecology Laboratory, Ocean Biology Processing Group, 2022; Dave Karl Lab, 2023; John, 2023; Ribalet et al., 2024) and were downloaded from the Simons Collaborative Marine Atlas Project (https://simonscmap.com) (Ashkezari et al., 2021) (Supplementary Data Sheet S7). Dissolved iron concentrations measured on station not underway were measured at depths ≤ 50 m, and were averaged by station following Hawco et al. (2025). Nitrate/nitrite concentrations were taken from depths < 24 m for the surface transects. Surface PAR was averaged daily with a spatial resolution of 9 km from the 469, 555, and 645 bands of the Moderate Resolution Imaging Spectroradiometer (MODIS) Aqua satellite, then averaged by latitude across the respective cruise's sampling dates. The temperature for each surface transect was averaged by latitude. Heterotrophic bacteria biomass was obtained from an Influx Cell Sorter from depths of ≤ 15 m, then averaged by cruise, latitude, and longitude. Nitrate/nitrite concentrations were not available for the same depth profile casts as the metatranscriptomes, so casts with nitrate/nitrite measurements from the same station were used for each depth profile: station 6, cast 1 at 32.93°N; station 5, cast 1 at 37°N; station 4, cast 10 at 41.68°N; and station 8, cast 3 at 42.33°N.

### Sequence data processing

Metatranscriptomic samples were extracted, eukaryotic mRNAs were poly(A)-selected, internal standards were prepared, and mRNA was used to construct libraries for Illumina RNA sequencing, as previously described (Groussman et al., 2024c). RNA sequences were trimmed, quality controlled, and *de novo* assembled, and transcripts were mapped against the *de novo* assemblies according to previously described methods (Groussman et al., 2021, 2024c) (Supplementary Figure S1b). Transcripts from the G2 incubations were mapped to the G2 surface assembly. Contigs were functionally annotated using the Pfam database (Bateman et al., 2004) with hmmsearch (Finn et al., 2011) using “cut_tc” trusted cutoff score. Additionally, only Pfam annotations with an e-value less than 1e-05 were retained. Contigs were taxonomically annotated with Diamond last common ancestor (Buchfink et al., 2015), retaining hits with an e-value less than 1e-05 and within 10% of the best bit score using the Marine Functional EukaRyotic Reference Taxa (MarFERReT) reference sequence library (Groussman et al., 2023a, 2024c) (Supplementary Figure S1b). For the samples not included in the North Pacific Eukaryotic Gene Catalog (Groussman et al., 2024c), the following software was used: G2 incubations: trimmomatic (version 0.36) (Bolger et al., 2014), kallisto (version 0.50.0) (Bray et al., 2016); G3 depth profiles: trimmomatic (version 0.39) (Bolger et al., 2014), trinity (version 2.15.1) (Grabherr et al., 2011), kallisto (version 0.46.1) (Bray et al., 2016), transeq (version 6.6.0.0) (Rice et al., 2000), hmmsearch (version 3.3) (Finn et al., 2011) against the Pfam database (version 34.0) (Bateman et al., 2004), and diamond (version 2.0.5.143). For each metatranscriptomic sample, contigs and their mapped transcripts were aggregated into species bins based on their taxonomic annotation—reflecting the closest known relative in the reference database rather than definitive species identities (Groussman et al., 2024c). We subsequently analyzed these species bins across samples, while recognizing that identical species bin labels may represent distinct species as contigs were grouped solely based on their closest species-level annotation.

To normalize sequence reads to TPM, the estimated number of reads mapped to each contig—outputted by the mapping software kallisto (Bray et al., 2016)—was divided by the contig nucleotide length to generate reads per kilobase (RPK). The RPK for each contig was summed by species bin and sample, and then divided by 1 million to generate a conversion factor. The RPK, divided by the conversion factor, yields the TPM per contig. TPMs were summed by Pfam for each species bin and each sample (Supplementary Figure S1b). Feature Pfams with zero transcripts mapped in a given species bin/sample were assigned TPM counts of zero. The TPM counts were scaled between zero and one with Scikit-learn's MinMaxScaler.

### Criteria for application of the model to environmental metatranscriptomes

We made trophic predictions for environmental species bins only because the models were trained on transcriptomes from individual species. The transcriptional completeness of species bins was evaluated using the eukaryotic Core Transcribed Genes (CTG), the 605 Pfams present in translated transcriptomes of ≥95% of the eukaryotic species in the MarFERReT reference library (Groussman et al., 2023a). We required each species bin to have at least one mapped read to ≥70% of the CTGs in a given sample for a trophic prediction to be made; this cutoff was chosen as 70% completeness is the standard for high-quality metagenome-assembled genomes (Benoit et al., 2024). The scaled TPM data for each species bin and sample that met this criterion was used as input for the trained machine learning model to generate trophic predictions. To exclude predictions likely resulting from model failure—since phototrophy and heterotrophy should not be expected for the same species bin under the same conditions—for the G1–G3 surface samples, we removed all trophic predictions for a species bin at a given latitude if both phototrophy and heterotrophy each accounted for more than 25% of predictions across replicates and size fractions (Supplementary Figure S1b). A similar exclusion criterion was applied to G3 depth profile samples (per latitude and depth), G2 incubation samples (per latitude, treatment, and time point), and ALOHA and G3 diel samples (per date) (Supplementary Figure S1b).

We determined the trophic capabilities of species bins based on the variety of their *in situ* trophic mode predictions across metatranscriptomic samples, rather than using a model trained to predict inherent trophic capabilities. This approach was necessary because transcriptomes reflect gene expression at the time of sampling, not the full genetic potential (genome) of the species. Specifically, a species bin was labeled as having mixotrophic capabilities if ≥23% of its predictions aggregated across the G1–G3 surface, ALOHA diel, G2 incubation, G3 diel, and G3 depth samples (after excluding trophic predictions determined to be model failure) differed from its dominant trophic mode (trophic mode with the most predictions for the species bin across the G1–G3 surface, ALOHA diel, G2 incubation, G3 diel, and G3 depth samples), and the different trophic predictions were not just split between replicates or size fractions. If >77% of a species bin's trophic predictions aggregated across metatranscriptomic samples were for mixotrophy, the species bin would also be labeled as having mixotrophic capabilities. If a species bin was not determined to have mixotrophic capabilities, it was labeled a phototrophic or heterotrophic specialist based on its dominant trophic mode across predictions.

We integrated *in situ* trophic predictions of species bins with mixotrophic capabilities across the G1–G3 surface transects with surface measurements of nitrate/nitrite, iron, PAR, temperature, and the biomass of *Prochlorococcus, Synechococcus*, heterotrophic bacteria, and picoeukaryotes. Observations were aligned based on cruise and proximity in latitude (within 0.5°), selecting the closest metadata measurement or averaging in the case of ties. Generalized Additive Models (GAMs) were then applied to quantify the partial effects of these environmental variables on the number of predictions for each trophic mode. To account for multiple hypothesis testing across environmental variables and trophic modes, we applied the Benjamini–Hochberg method. There were not enough trophic predictions to apply a similar analysis to the G3 depth profiles.

### Distribution of environmental species bins based on transcript abundance

Transcript concentrations of species bins across the North Pacific Ocean were calculated following the methods of Groussman et al. (2024c). Trimmed paired-end reads were merged with Fast Length Adjustment of SHort reads (FLASH) (minimum overlap 150 bp, maximum overlap 250 bp, and maximum mismatch ratio 25%) (Magoč and Salzberg, 2011), then merged reads were mapped to the spiked-in custom standards using bowtie2 with default parameters (Langmead and Salzberg, 2012). For the samples not included in the North Pacific Eukaryotic Gene Catalog (Groussman et al., 2024c), the following software was used: G2 incubations: FLASH (version 1.2.11) (Magoč and Salzberg, 2011) and bowtie2 (version 2.5.2) (Langmead and Salzberg, 2012); G3 depth profiles: FLASH (version 1.2.11) (Magoč and Salzberg, 2011) and bowtie2 (version 2.5.2) (Langmead and Salzberg, 2012). Transcript abundance was summed between size fractions when different size fractions of metatranscriptomes were collected from the same sample. The number of transcripts per liter was calculated based on counts of the custom standards (Groussman et al., 2024c). Transcript concentrations correlate with cellular carbon biomass across broad taxonomic groups, with dinoflagellates containing 6.4 times more transcripts per unit of carbon biomass compared to other plankton taxonomic groups (Coesel et al., 2025). To compare abundance across taxonomic groups using transcripts, we scaled down the dinoflagellate transcript concentrations by a factor of 6.4.

Changes in transcript abundance across latitude were analyzed for each trophic group (species bins with mixotrophic capabilities, phototrophic specialists, and heterotrophic specialists) using GAMs. Additionally, for each surface transect, a GAM assessed the effect of latitude on transcript abundance, while another incorporated an interaction term to allow the relationship to vary among trophic groups. An ANOVA was used to compare these models, testing whether the effect of latitude on transcript abundance differed significantly among trophic groups. The same analysis was run separately for each cruise for the latitudes south and north of the salinity isohaline (34.82).

Bulk chlorophyll *a* was compared between control and nutrient amendment treatments across the G2 incubations grouping by station with ANOVAs followed by *post-hoc* Tukey tests. Transcript abundance of species bins was compared between control and nutrient amendment treatments across the G2 incubations with one-tailed *t*-tests followed by multiple hypothesis testing correction grouped by latitude and treatment using the Benjamini–Hochberg method. Transcript abundance across depth profiles for each trophic group was compared with one-way ANOVAs. Transcript abundance between trophic groups at each depth of the depth profiles was evaluated with one-way ANOVAs.

## Results

### Model development and testing

Since the development of the original Lambert et al. (2022) machine learning model to predict the *in situ* trophic mode of marine protist species based on the expression of Pfams, high contamination levels or low sequence abundance were identified for ~13% (59/446) of the MMETSP-derived transcriptomes in the training dataset (Lasek-Nesselquist and Johnson, 2019; Van Vlierberghe et al., 2021; Groussman et al., 2023a). We evaluated whether model performance was improved with a refined version of the training dataset that was trained using fewer but only high-quality transcriptomes. We compared two MMETSP-derived training datasets—one that consisted of the TPM expression of Pfams for the 446 transcriptomes that could be assigned a trophic mode as described by Lambert et al. (2022), and another that consisted of the TPM expression of the Pfams for the 387 transcriptomes that could be assigned a trophic mode and passed the metrics of at least 1,200 total sequences, at least 500 total assigned Pfam domains, and < 50% non-target sequences. Both datasets were used to train XGBoost and Random Forest models. The best-performing hyperparameters were selected for each model and training dataset (Supplementary Data Sheet S8). We evaluated model performance given the different training datasets and software versions of the XGBoost (0.90 vs. 1.7.4) and Random Forest (0.21.3 vs. 1.5.1) models.

First, we compared the number of features identified via mean decrease in accuracy between the originally used and updated software versions of the XGBoost and Random Forest models using the original training dataset that included the contaminated and low-sequence transcriptomes. We expected a reduction in the number of feature Pfams with the updated software versions, as we predicted algorithmic improvements would enhance the models' ability to filter out less informative features. Use of the updated software versions decreased the number of feature Pfams identified with the XGBoost model from 265 to 260. Similarly, the number of feature Pfams identified with the Random Forest model decreased from 901 to 593. A reduction in feature Pfams also occurred when the cleaned training dataset, rather than the original training dataset, was used along with updated software: the number of features identified with the XGBoost model was reduced from 260 to 183, and the number of features identified with the Random Forest model was reduced from 593 to 511. Thus, for the XGBoost model, the greatest reduction in features resulted from the use of the cleaned training dataset, whereas for the Random Forest model, the greatest reduction in features resulted from the use of updated software. We used the updated software versions of the models for further evaluation.

We next examined whether the XGBoost or Random Forest models yielded better performance based on F1 score when the two models were trained with the original or the cleaned training dataset. There was no significant difference (one-way ANOVAs, *p*-values > 0.05) in performance of the XGBoost and Random Forest models in overall performance or performance by trophic mode, depending on whether the original or cleaned training dataset was used (Supplementary Figure S2). Further, we found no significant difference (one-way ANOVAs, *p*-values > 0.05) in performance of the XGBoost model based on overall performance or performance by trophic mode when the feature set contained only the Pfams determined essential for the XGBoost model or the union of features determined essential for the XGBoost and Random Forest models (Supplementary Figure S2). Overall model performance was high—mean F1 scores >0.9—across all of these model variations (Supplementary Figure S2a). Each of the models had the lowest performance for mixotrophic transcriptomes, although each still had a mean F1 score >0.8 for this trophic mode (Supplementary Figure S2b). We chose the cleaned dataset for training, the XGBoost model, and the 183 XGBoost feature Pfams for subsequent analyses as this combination performed well overall (mean F1 score = 0.94, standard error = 1.5e-02), individually for mixotrophy predictions (mean F1 score = 0.89, standard error = 2.5e-02), and relied on the fewest feature Pfams. We name this model MarPRISM (https://github.com/armbrustlab/MarPRISM).

The assignment of trophic mode labels to the transcriptomes in the training dataset may contain errors. One specific case of uncertainty in the training dataset is *Micromonas*, for which the literature is divided regarding its mixotrophic capabilities (e.g., McKie-Krisberg et al., 2018; Jimenez et al., 2021). We performed cross-validation on a modified version of the training dataset—in which *Micromonas* transcriptomes originally labeled mixotrophic were labeled phototrophic, and observed no significant difference (oneway ANOVAs, *p*-values > 0.05) in overall or trophic mode-specific performance (Supplementary Figure S2).

To evaluate the impact of feature expression on model performance, we compared MarPRISM using TPM-based Pfam expression values to a version of the model using binarized expression (where TPM > 0 was set to 1). MarPRISM identified 183 feature Pfams when using TPM values, whereas 245 feature Pfams were required when using binarized expression data. Thirty-seven of the Pfams were features used by both models. Using hyperparameters optimized for the binarized expression model (Supplementary Data Sheet S8), we observed no significant difference (one-way ANOVAs, *p*-values > 0.05) in overall or trophic mode-specific performance between the TPM- and binary-based XGBoost models (Supplementary Figure S2). We selected MarPRISM for downstream analysis because it uses continuous expression data—which contains more information—and achieved comparable performance with fewer features, suggesting it captures trophic mode signals more efficiently.

Cross-validation was further used to explore the performance of MarPRISM. A cumulative confusion matrix based on cross-validation showed that MarPRISM most often correctly predicted the expected trophic mode (Supplementary Figure S3a). The most common error was misclassifying mixotrophic transcriptomes as phototrophic (Supplementary Figure S3a). Other errors were rare, including a few phototrophic transcriptomes predicted as mixotrophic, and even fewer predicted as heterotrophic (Supplementary Figure S3a). The previous version of the model (Lambert et al., 2022) also most often predicted the correct trophic mode, but shared the same primary error: mixotrophic transcriptomes being misclassified as phototrophic (Supplementary Figure S3b). Like MarPRISM, it made few errors overall, including occasional phototrophic-to-mixotrophic and phototrophic-to-heterotrophic misclassifications of transcriptomes (Supplementary Figure S3b). However, unlike MarPRISM, it also misclassified a small number of heterotrophic transcriptomes as mixotrophic (Supplementary Figure S3b).

**Figure 1:**
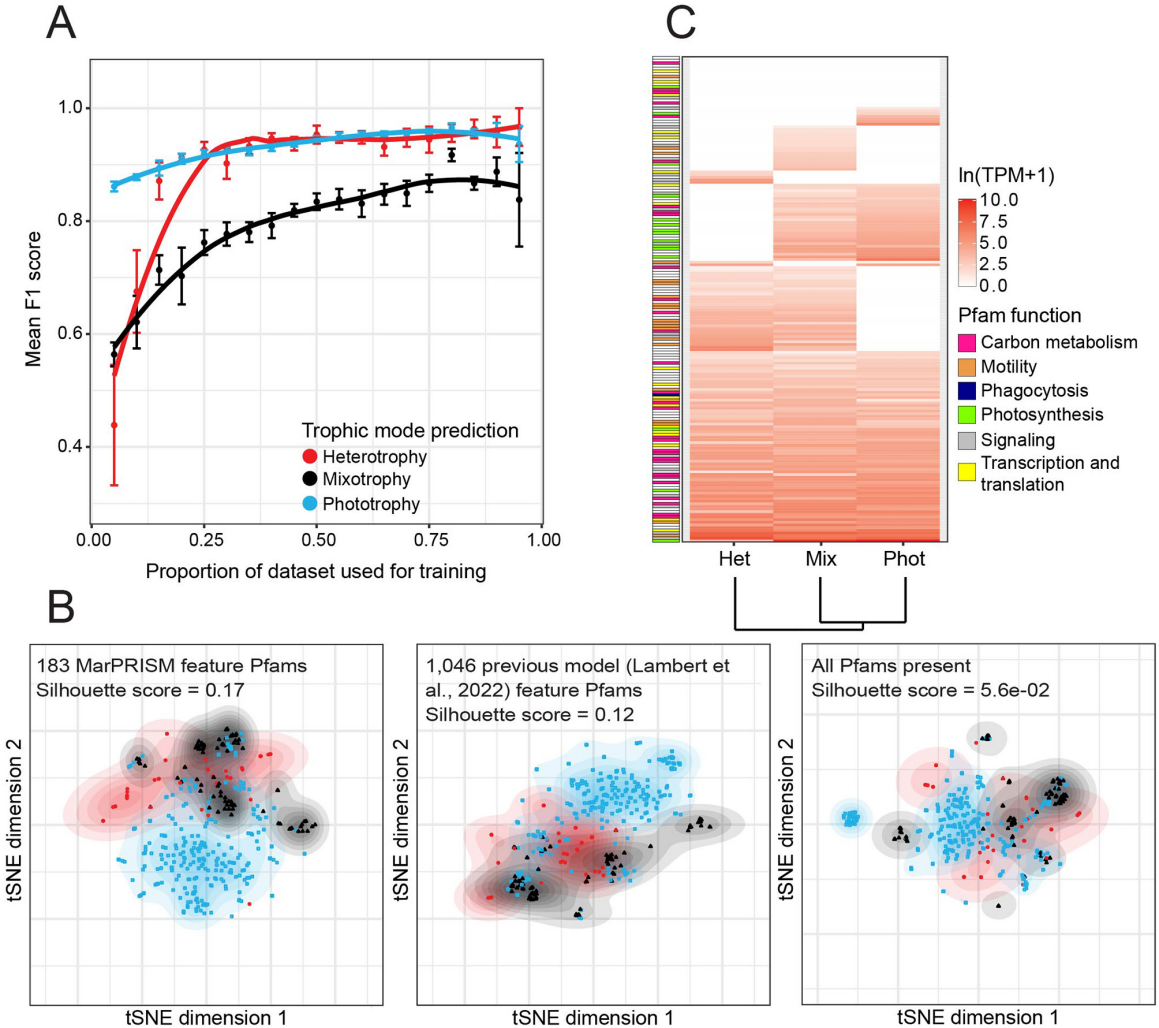
Performance of MarPRISM evaluated using the training dataset. **(A)** Mean F1 [2 ×precision × recall/(precision + recall)] score by trophic mode for MarPRISM against the proportion of MarPRISM training dataset transcriptomes used for training, determined from 6-fold cross-validation. Error bars represent the standard error of 6-fold cross-validation. **(B)** Separation of the heterotrophy, mixotrophy, and phototrophy training dataset entries based on t-distributed stochastic neighbor embedding (*t*-SNE) using the 183 feature Pfams of MarPRISM, 1,046 feature Pfams of the previous version of the model (Lambert et al., 2022), or all Pfams present in the MarPRISM training dataset. The expression of Pfams in transcripts per million (TPM) was normalized by mean and standard deviation, then averaged (median) by trophic mode label across the training dataset transcriptomes. Contours represent 2D-kernel density estimations for transcriptomes of each trophic mode. The mean silhouette score, a measurement of consistency within clusters, is included for each set of Pfams. **(C)** Median TPM averaged by trophic mode for the 183 MarPRISM feature Pfams in the MarPRISM training dataset transcriptomes. The median TPM values of the feature Pfams were natural log-transformed, scaled, and then the trophic modes were hierarchically clustered based on Euclidean distance. The feature Pfams were arranged based on the trophic modes for which they had non-zero median expression, and their maximum median expression. Feature Pfams were manually grouped into broad functional categories: carbon metabolism (pink), motility (orange), phagocytosis (navy), photosynthesis (green), signaling (gray), and transcription and translation (yellow).

MarPRISM's sensitivity to the number of transcriptomes used for training was quantified with cross-validation. The mean F1 score increased with the proportion of the cleaned training dataset used for training, leveling off but never decreasing for each trophic mode, suggesting the model was not overfit (Figure 1A). Even with only 75% of the training dataset used, F1 scores remained close to those obtained using the full dataset for each trophic mode (Figure 1A).

The number of feature Pfams used by MarPRISM was reduced from the previous version of the model (Lambert et al., 2022), from 1,046 to 183. To evaluate the impact of this reduction, we analyzed how the training dataset transcriptomes separated based on different feature sets using *t*-SNE analysis. The training dataset transcriptomes separated more clearly based on silhouette score (silhouette scores range from −1 to 1) when *t*-SNE analysis was performed on the scaled TPM expression of the 183 feature Pfams (mean silhouette score = 0.17) than when performed on either the 1,046 previous feature Pfams (mean silhouette score = 0.12) or all of the Pfams present in the training dataset transcriptomes (mean silhouette score = 5.6e-02) (Figure 1B). The phototrophic and heterotrophic transcriptomes separated well based on the 183 MarPRISM feature Pfams; however, there was still some overlap between trophic modes (Figure 1B).

We analyzed MarPRISM's feature Pfams (Supplementary Data Sheet S9) to evaluate how these Pfams distinguished between species behaving heterotrophically, mixotrophically, and phototrophically. Around 65% (117/183) of the feature Pfams used by MarPRISM were also used by the previous version of the model (Lambert et al., 2022). Around 20% (36/183) of the feature Pfams were eukaryotic CTGs (Groussman et al., 2023a). The mean TPM of the 183 feature Pfams (307.22) was significantly higher (two-tailed *t*-test, *p*-value = 1.5e-113) than the mean TPM of the other Pfams in the cleaned training dataset (39.54). We evaluated the expression of MarPRISM's feature Pfams across the cleaned training dataset (Figure 1C, Supplementary Data Sheet S9). The largest proportion of feature Pfams, 72/183 (39%), had non-zero median expression across each set of transcriptomes: phototrophic, mixotrophic, and heterotrophic—indicating expression in at least half of the transcriptomes in each set. Around 35% (63/183) of the feature Pfams had non-zero median expression in two trophic modes. Of the feature Pfams expressed in two trophic modes, the largest proportion was shared between the mixotrophic and heterotrophic transcriptomes (32/183, 17%), followed by the phototrophic and mixotrophic transcriptomes (29/183, 16%); two feature Pfams were shared between the phototrophic and heterotrophic transcriptomes (2/183, 1%). Approximately 16% (29/183) of the feature Pfams had non-zero median expression in just one trophic mode: 17/183 (9%) in the mixotrophic, 7/183 (4%) in the phototrophic, and 5/183 (3%) in the heterotrophic transcriptomes.

Based on annotation, we categorized ~60% (106/183) of the feature Pfams into six broad functions: carbon metabolism, motility, phagocytosis, photosynthesis, signaling, and transcription and translation (Figure 1C, Supplementary Data Sheet S9). We examined the expression of these feature Pfams across the training dataset transcriptomes. Photosynthesis-related feature Pfams were characteristic of phototrophic and mixotrophic transcriptomes: 13/15 (87%) photosynthesis-related feature Pfams had non-zero median expression in the phototrophic and mixotrophic transcriptomes compared to 3/15 (20%) in the heterotrophic transcriptomes (Figure 1C, Supplementary Data Sheet S9). One of the feature Pfams, the ELMO/CED-12 family, is a domain involved in phagocytosis of apoptotic cells in mammals (Gumienny et al., 2001) (Figure 1C, Supplementary Data Sheet S9). This Pfam had non-zero median expression across all three trophic modes but exhibited higher median expression in the heterotrophic (42.53 TPM) and mixotrophic (29.64 TPM) transcriptomes than the phototrophic transcriptomes (18.21 TPM) (Figure 1C, Supplementary Data Sheet S9). Motility-related feature Pfams were characteristic of mixotrophic and heterotrophic transcriptomes: a third (7/21) of the motility-related feature Pfams had non-zero median expression in the phototrophic transcriptomes compared to almost all of the motility-related feature Pfams in the mixotrophic and heterotrophic transcriptomes, 19/21 (90%) and 18/21 (86%), respectively (Figure 1C, Supplementary Data Sheet S9). The feature Pfams related to carbon metabolism, signaling, and transcription and translation did not show clear patterns across the training dataset transcriptomes (Figure 1C, Supplementary Data Sheet S9).

We quantified the performance of MarPRISM by testing its ability to make trophic predictions for 27 cultured protist transcriptomes not included in the training dataset. MarPRISM correctly predicted the expected trophic mode across all transcriptomic replicates for 78% (21/27) of the protist cultures (Supplementary Data Sheet S3). When replicates were considered individually, MarPRISM correctly predicted the expected trophic mode for 79% (60/76) of the transcriptomes (Supplementary Data Sheet S3). For comparison, the previous version of the model Lambert et al., (2022) correctly predicted the expected trophic mode for 81% (22/27) of the protist cultures across all of the transcriptomic replicates, and 82% (62/76) of the individual replicate transcriptomes (Supplementary Data Sheet S3). MarPRISM and the previous version of the model differed in which cultures they correctly predicted. Unlike the previous version of the model, MarPRISM accurately predicted *Ochromonas* sp. CCMP1393 grown with bacteria in the dark to be behaving mixotrophically (expected trophic mode was mixotrophy or heterotrophy) (Supplementary Figure S4, Supplementary Data Sheet S3). Unlike the previous version of the model, MarPRISM incorrectly predicted *Ochromonas* sp. BG-1 grown with bacteria in the dark to be behaving mixotrophically rather than heterotrophically, and *Chrysochromulina* sp. AL-TEMP12 grown with bacteria in the light sampled at night to be growing phototrophically rather than mixotrophically (Supplementary Figure S4, Supplementary Data Sheet S3). Notably, both models correctly predicted the non-photosynthetic diatom *Nitzschia* sp. Nitz4 to be growing heterotrophically (Supplementary Figure S4, Supplementary Data Sheet S3), despite all diatom transcriptomes in the training dataset being derived from phototrophically growing species. Also of interest, given the ongoing debate about *Micromonas'* trophic capabilities (McKie-Krisberg et al., 2018; Jimenez et al., 2021), both models predicted *M. polaris*—when grown in the light with bacteria under both high and low nutrient conditions—to be growing mixotrophically (expected trophic mode for high nutrient conditions was phototrophy or mixotrophy) (Supplementary Figure S4, Supplementary Data Sheet S3). We acknowledge that the measured accuracy of the models—based on their performance on the test transcriptomes—depends on the expected trophic mode labels we assigned to those transcriptomes. If we followed the findings of Jimenez et al. (2021) and assumed that *Micromonas* lacks mixotrophic capabilities, we would label all of the *M. polaris* transcriptomes used for testing as phototrophic. Given this change, MarPRISM would correctly predict the expected trophic mode of 70% (19/27) of protist cultures across all transcriptomic replicates and 68% (52/76) of individual transcriptomes. If we excluded *M. polaris* from the test transcriptomes, MarPRISM's accuracy would be 76% for both protist cultures across all transcriptomic replicates (19/25) and individual transcriptomes (52/68).

Finally, we tested the ability of MarPRISM to predict the expected trophic mode of MMETSP-derived transcriptomes removed from the training dataset due to low sequence abundance or high rates of contamination (Supplementary Data Sheet S4). MarPRISM correctly predicted the expected trophic mode of 78% (46/59) of these transcriptomes, which is comparable to the accuracy of MarPRISM's predictions for the 27 cultured protist transcriptomes despite the potential noisiness of the transcriptomes removed from the training dataset. MarPRISM's performance was not compared to the previous version of the model for these transcriptomes, as these contaminated or low-sequence transcriptomes were included in the training of the (Lambert et al. 2022) model. Overall, MarPRISM and the previous version of the model performed comparably, correctly predicting the expected trophic mode for ~80% of the test transcriptomes. We estimate a potential error rate of 23% for MarPRISM, with more confidence in predictions that align between replicates and size fractions.

### Confidence in trophic predictions for environmental species bins

We defined sufficient coverage for a trophic mode prediction to be generated as the expression of at least 70% of eukaryotic CTGs (Groussman et al., 2023a) within a given environmental species bin and sample. We noted that the transcriptomes for some species bins were split between size fractions, likely reflecting species whose size distribution spanned the 3 μm filter fraction. The total number of trophic predictions and the number of species-level taxa for which predictions were possible depended on the cutoff used to define sufficient species bin coverage for assigning a trophic prediction (Supplementary Figure S5). However, the selected 70% CTG recovery threshold lies near the tail end of the distributions, where both metrics plateaued (Supplementary Figure S5). Consequently, the number of predictions and species-level taxa with trophic predictions varied little around this threshold.

We used multiple approaches to quantify the reliability of MarPRISM trophic predictions for species bins within the North Pacific Ocean metatranscriptomes. First, we examined trophic predictions for species bins that were split between phototrophy and heterotrophy across replicates and size fractions and were thus excluded, as these predictions were hypothesized by Lambert et al. (2022) to conflict with model decision boundaries. Given this criterion, 0–9% of predictions for species bins/sample pairs were excluded (Supplementary Data Sheet S10). For the G1 and G2 surface samples, ~7% of species bin/sample pairs were excluded (G1: 21/315; G2: 21/298). For the G3 surface and depth samples, ~1.5% (3/211) and 3% (4/146) of species bin/sample pairs were excluded, respectively. For both diel studies, 0% of species bin/time point pairs were excluded (ALOHA diel: 0/162; G3 diel: 0/147). Approximately 9% (23/255) of the species bin/treatment/time point pairs were excluded from the G2 incubation samples. Eight species bins had trophic predictions excluded, and the following four species bins had high percentages of predictions excluded: 20–33% of predictions for *Brandtodinium nutricula, Scrippsiella trochoidea, Pelagodinium beii*, and *Prorocentrum minimum* resulted in split predictions of heterotrophy and phototrophy between replicates and size fractions (Supplementary Data Sheet S11). We excluded these trophic predictions that were split between phototrophy and heterotrophy between replicates and size fractions.

Second, we evaluated whether the reduced number of feature Pfams used by MarPRISM resulted in sensitivity to sequencing coverage. For each species bin that met the 70% CTG recovery cutoff, we quantified the proportion of trophic predictions in agreement across replicate metatranscriptomes and size fractions—after excluding trophic predictions assumed to have resulted from model failure—under the premise that divergence in these predictions reflected prediction uncertainty. Above the cutoff of 70% CTG recovery, the proportion of trophic predictions in agreement across replicates and size fractions was significantly but weakly negatively correlated (Kendall rank correlation test, tau = −0.13, *p*-value = 2.4e-04) with CTG coverage of the species bins (Supplementary Figure S6). Thus, above the 70% CTG recovery cutoff, species bins with lower CTG coverage did not receive more uncertain trophic predictions than species bins with higher CTG coverage. Most trophic predictions were in agreement across replicates and size fractions (Supplementary Figure S6). Phototrophy (mean = 0.98) and heterotrophy predictions (mean = 0.93) had more agreement among replicates and size fractions than mixotrophy predictions (mean = 0.80).

Lastly, we evaluated the reproducibility of trophic predictions in two diel studies in which samples were collected with Lagrangian tracking every 4 h for either 4 (ALOHA diel) or 3 (G3 diel) days. Two species bins from the ALOHA diel study—*Karenia brevis* and *Karlodinium veneficum*—and three species bins from the G3 diel study—*Bathycoccus prasinos, Triparma pacifica*, and *Oxytricha trifallax*—received highly reproducible trophic predictions over the diel cycle (Supplementary Figure S7). The species bins identified as *K. brevis, K. veneficum*, and *O. trifallax* were consistently predicted as heterotrophic *in situ*, even for those time points where a single replicate could be tested (Supplementary Figure S7). The *B. prasinos* and *T. pacifica* species bins were consistently predicted as phototrophic (Supplementary Figure S7c). Two species bins corresponding to *Azadinium spinosum* and *P. minimum* received a mix of trophic predictions across the ALOHA diel study (Supplementary Figure S7a). Few samples had sufficient sequencing coverage for the *S. trochoidea* and *Prymnesium polylepis* species bins to make trophic predictions across the ALOHA diel study (Supplementary Figure S7a). Trophic predictions for a given species bin did not consistently differ across the diel cycle or days (Supplementary Figure S7), suggesting that the trophic mode of these species bins was stable over the daily cycle. In line with this, Connell et al. (2020) found no diel pattern in the grazing activity of mixotrophic nanoplankton at Station ALOHA. This allowed for a comparison of trophic predictions across G1–G3 in instances where samples were collected at different times of day.

### Trophic predictions elucidated the trophic capabilities of environmental species bins

A total of 28 environmental species bins had sufficient sequencing depth, as defined by ≥70% CTG recovery, to receive trophic predictions. After excluding predictions split between phototrophy and heterotrophy as previously described, a total of 1,462 trophic predictions were made for these 28 species bins across 335 metatranscriptomic samples (Supplementary Data Sheets 12, 13). Most trophic predictions were for dinoflagellate species bins (676), followed by pelagophytes (203), ciliates (184), chlorophytes (167), bolidophytes (109), haptophytes (86), dictyochophytes (36), and MAST-4 cells (1) (Supplementary Data Sheet S13). The 28 species bins with trophic predictions were abundant (Supplementary Data Sheet S14), representing 47% of the transcript abundance of protists identified at the species level across the ALOHA diel samples, 76% across the G1 surface transect, 67% across the G2 surface transect, 45% across the G2 incubation samples, 71% across the G3 surface transect, 61% across the G3 diel samples, and 55% throughout the G3 depth profiles.

We aimed to identify which species bins displayed mixotrophic capabilities—i.e., the ability to shift trophic mode between phototrophy, mixotrophy, and/or heterotrophy in response to environmental conditions. MarPRISM had an accuracy rate of ~77% when tested on cultured protist transcriptomes not included in the training dataset (Supplementary Data Sheets S3, S4), indicating an estimated 23% error rate for our trophic predictions. This could result in a species bin appearing to have variable trophic predictions across field samples due to model error rather than a true biological signal. No species bin received primarily mixotrophy predictions. Thus, for a species bin to be labeled as having mixotrophic capabilities, ≥23% of the species bin's predictions aggregated across all of the field samples had to differ from its dominant trophic mode (the trophic mode with the most predictions across all of the field samples). This threshold is statistically justified as it corresponds to the estimated model error rate, thereby accounting for prediction noise. To ensure that observed trophic variability was reproducible, we required that the different trophic predictions could not be attributed solely to replicates or size fractions for a species bin to be labeled as having mixotrophic capabilities (Supplementary Figure S1b). This approach minimized false positives arising from model error.

**Figure 2:**
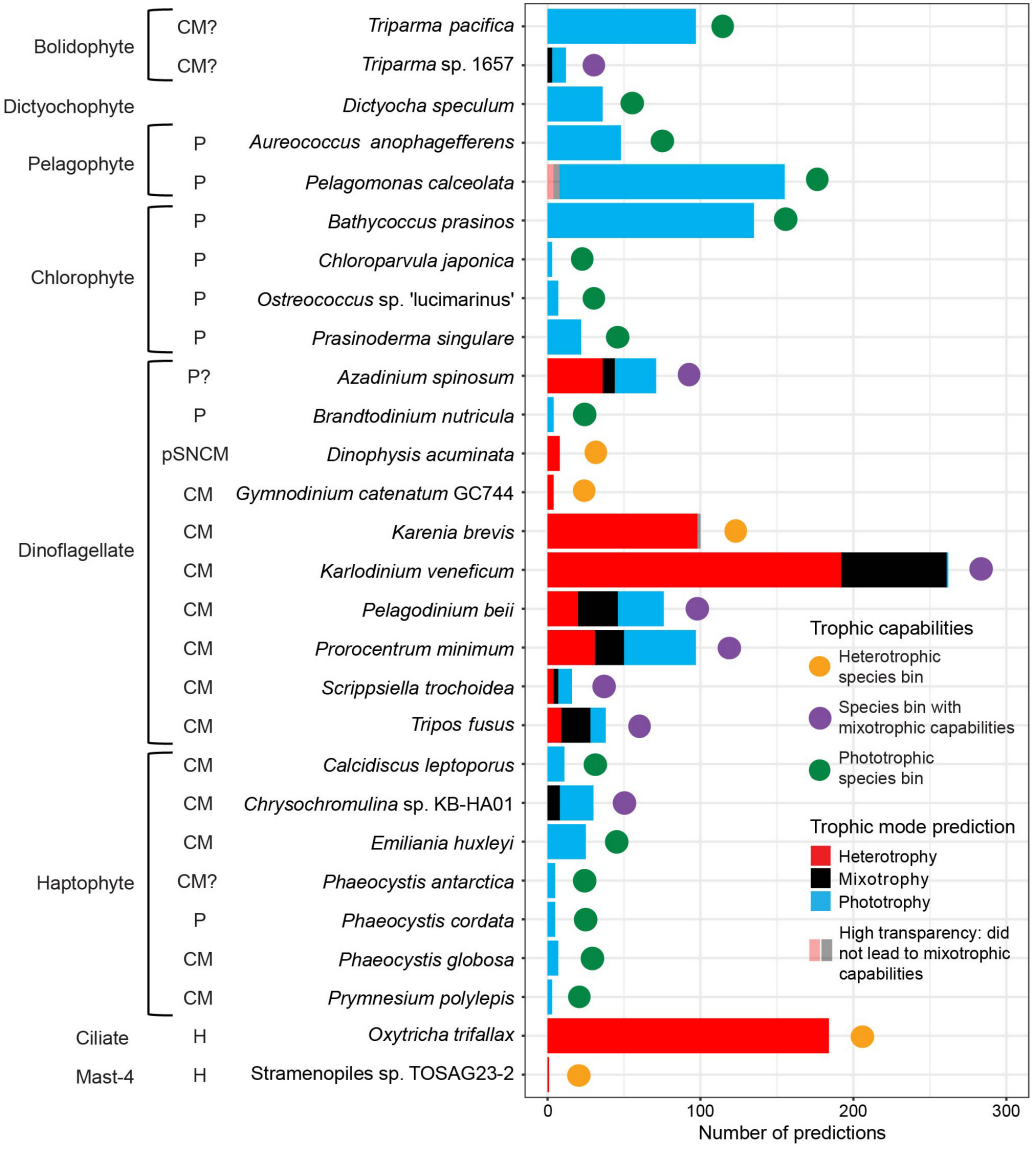
Number of trophic predictions and trophic capabilities of environmental species bins across five cruise datasets: G1–G3 surface, ALOHA diel, G2 incubation, G3 diel, and G3 depth profile samples. Trophic predictions were summed by species bin across replicates and size fractions. Species bins are labeled with the species name of their closest relative based on last common ancestor analysis, and whether previous literature (Nygaard and Tobiesen, 1993; Chang and Carpenter, 1994; Jacobson and Andersen, 1994; Havskum and Riemann, 1996; Li et al., 1996; Stoecker et al., 1997; Li et al., 1999, 2001; Jeong et al., 2005; Calbet et al., 2011; Tillmann et al., 2014; Gast et al., 2018; Glibert et al., 2009; Avrahami and Frada, 2020; Koppelle et al., 2022; Lambert et al., 2022; Li et al., 2022), many collected by Mitra et al. (2023a), indicated their closest relative to be a phototroph (P), heterotroph (H), constitutive mixotroph (CM), or plastidic specialist non-constitutive mixotroph (pSNCM), followed by? if the trophic capabilities were uncertain due to disagreement or low taxonomic resolution in the literature, or whether the trophic capabilities of their closest relative were unknown (no label). Trophic capabilities of the species bins were defined as follows. Mixotrophic capabilities (purple circle): ≥23% of its predictions were assigned trophic mode(s) different from its majority trophic mode, and the different trophic predictions were not solely split between replicates or size fractions. Heterotrophic (orange circle): received all or almost all heterotrophy predictions (<23% non-heterotrophy predictions). Phototrophic (green circle): received all or almost all phototrophy predictions (<23% non-phototrophy predictions). Trophic predictions that did not lead to a species bin being labeled as having mixotrophic capabilities are marked with high transparency: heterotrophy and mixotrophy predictions for *Pelagomonas calceolata* and mixotrophy predictions for *Karenia brevis*.

Based on this criterion, eight species bins were defined as having mixotrophic capabilities (Figure 2). These species bins were phylogenetically diverse, with the closest relatives *Triparma* sp. 1657, *Chrysochromulina* sp. KB-HA01 and the dinoflagellates *A. spinosum, K. veneficum, P. beii, P. minimum, S. trochoidea*, and *Tripos fusus* (Figure 2). The proportion of phototrophy, mixotrophy, and heterotrophy predictions varied across these species bins. *P. beii* was the only species bin to have a relatively similar number of predictions for each trophic mode (Figure 2, Supplementary Data Sheet S15). Both the *Triparma* sp. 1657 and *Chrysochromulina* sp. KB-HA01 species bins received ~75% phototrophy and 25% mixotrophy predictions (Figure 2, Supplementary Data Sheet S15). The *P. minimum* and *S. trochoidea* species bins received ~50% phototrophy, 30% heterotrophy, and 20% mixotrophy predictions (Figure 2, Supplementary Data Sheet S15). The *K. veneficum* species bin received ~75% heterotrophy predictions and 25% mixotrophy predictions (Figure 2, Supplementary Data Sheet S15). The *A. spinosum* species bin received ~50% heterotrophy, 40% phototrophy, and 10% mixotrophy predictions (Figure 2, Supplementary Data Sheet S15). The *T. fusus* species bin received ~50% mixotrophy, 25% phototrophy, and 25% heterotrophy predictions (Figure 2, Supplementary Data Sheet S15). Thus, three of these species bins were around the threshold for being labeled as having mixotrophic capabilities: *Triparma* sp. 1657 had 25% of predictions differing from its dominant trophic mode, while both *Chrysochromulina* sp. KB-HA01 and *K. veneficum* had 27%.

For the species bins not found to have mixotrophic capabilities, the dominant trophic mode across the species bin's aggregated predictions determined its trophic specialization. Fifteen species bins were defined as phototrophic specialists, including all the pelagophyte, chlorophyte, and the majority of haptophyte species bins, as well as one bolidophyte, one dictyochophyte, and one dinoflagellate species bin (Figure 2). Of these, only the species bin identified as *Pelagomonas calceolata* displayed predictions−5% (8/155)—other than phototrophy (Figure 2, Supplementary Data Sheet S15). Five species bins were defined as heterotrophic specialists: the dinoflagellates *Dinophysis acuminata, Gymnodinium catenatum* GC744, and *K. brevis*, the ciliate *O. trifallax*, and the MAST-4 flagellate Stramenopiles sp. TOSAG23-2 (Figure 2). Of these, only the *K. brevis* species bin displayed predictions –2% (2/100)—other than heterotrophy (Figure 2, Supplementary Data Sheet S15).

We compared trophic predictions for the environmental species bins to literature-reported trophic capabilities of their closest relatives. Of the eight species bins predicted to have mixotrophic capabilities, seven had a closest relative either known or hypothesized to be a constitutive mixotroph with the ability to photosynthesize and ingest prey (Figure 2, Supplementary Data Sheet S15). Trophic predictions found the species bin identified as *A. spinosum* to have mixotrophic capabilities (Figure 2, Supplementary Data Sheet S15). Although little is known about the mixotrophic capabilities of *Azadinium* in the literature, so far, the genus has not been found to ingest prey (Tillmann et al., 2014). Of the 15 species bins we identified as phototrophic specialists, eight had a closest relative shown to be an obligate phototroph, six had a closest relative known or hypothesized to be a constitutive mixotroph, and one had a closest relative with no literature reports (Figure 2, Supplementary Data Sheet S15). Of the five species bins identified as heterotrophic specialists, two species bins had closest relatives known to be strictly heterotrophic, and three had closest relatives described as plastidic specialist non-constitutive or constitutive mixotrophs (Figure 2, Supplementary Data Sheet S15). The overall concurrence between our predictions and literature reports provided further confidence in the use of MarPRISM to predict the *in situ* trophic mode for species bins with high sequencing coverage.

### Intraspecies trophic changes observed across the surface ocean, nutrient incubations, and depth

**Figure 3:**
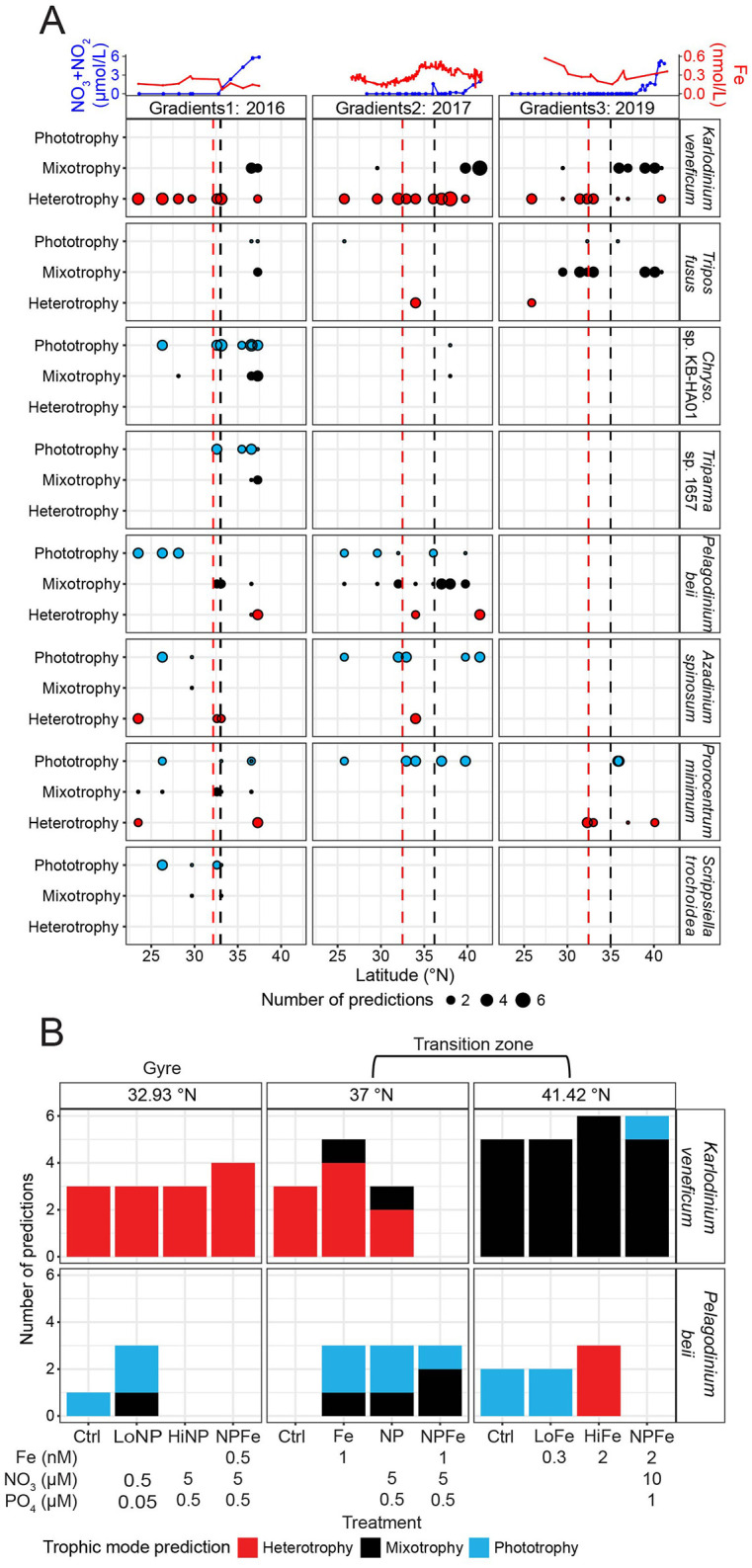
*In situ* trophic predictions across the surface ocean and G2 nutrient amendment incubations for the species bins defined as having mixotrophic capabilities. **(A)** Top panel: surface nitrate/nitrite (blue, μmol/L) and dissolved iron concentrations (red, nmol/L) across latitude. Lower panels: number of trophic predictions for species bins (taxonomy is indicated on the right side of panels; note genus abbreviation *Chryso*. is *Chrysochromulina*) across latitude, summed across replicates and size fractions. Red dashed line: location of salinity isohaline (34.82); black dashed line: location of transition zone chlorophyll front (0.15 mg m^−3^ chlorophyll). **(B)** Trophic predictions across G2 nutrient amendment incubations for the *Karlodinium veneficum* and *Pelagodinium beii* species bins summed across replicates and size fractions. *K. veneficum* and *P. beii* were the only species bins with mixotrophic capabilities that had sufficient trophic predictions across the G2 incubations to analyze. Different ratios of iron, nitrate, and phosphate were added to incubations of the surface water community collected from 15 m at 32.93, 37, and 41.42°N during G2. The control (no nutrient amendment) and nutrient-amended treatments were sampled after 96 h.

We next examined whether the trophic predictions for the eight species bins with mixotrophic capabilities displayed trophic shifts across the G1–G3 surface transects. We hypothesized that species would shift their trophic mode toward phototrophy with latitude, as Lambert et al. (2022) had found this to be the case for three species bins across the G1 surface transect, presumably due to higher nitrate availability in the transition zone reducing the need for prey ingestion. The *K. veneficum* species bin showed the most consistent trophic shifts across the three cruise transects. In the gyre, the *K. veneficum* species bin was predicted to be heterotrophic. In contrast, at the more northern stations, the species bin received only mixotrophy predictions or a combination of mixotrophy and heterotrophy predictions (Figure 3A). During G3, the *T. fusus* species bin showed a similar pattern to the *K. veneficum* species bin, as it was predicted to shift from heterotrophy within the gyre to mixotrophy predictions at the more northern stations. The *Chrysochromulina* sp. KB-HA01 and *Triparma* sp. 1657 species bins only had enough trophic predictions during G1 to analyze, and both displayed a shift from phototrophy toward mixotrophy with increasing latitude (Figure 3A). The *P. beii* species bin shifted from phototrophy to mixotrophy and heterotrophy with increasing latitude during G1 and G2 (Figure 3A). Neither the *A. spinosum, P. minimum*, nor *S. trochoidea* species bin displayed consistent trophic shifts with latitude (Figure 3A). Consistent with different species bins exhibiting distinct latitudinal shifts in *in situ* trophic mode, across the surface transects, for the species bins with mixotrophic capabilities, we did not find any significant partial effects (GAMs assessing partial effects, adjusted *p*-values > 0.05) between the number of predictions for each trophic mode and nitrate/nitrite, iron, PAR, temperature, or the biomass of *Prochlorococcus, Synechococcus*, heterotrophic bacteria, or picoeukaryotes (Supplementary Figure S8).

To disentangle the effects of nitrate and iron availability on trophic shifts, we examined trophic predictions of the *K. veneficum* and *P. beii* species bins across a series of on-deck experiments (Figure 3B). These were the only species bins with mixotrophic capabilities that had enough predictions across incubations to analyze. Three sets of nutrient amendment experiments of the surface water community, collected from 15 m, were conducted during G2: one with seawater samples at the gyre's northern edge at 32.93°N where macronutrient concentrations were low, one in the transition zone at 37°N where macronutrient concentrations continued to be low, and one in the transition zone at 41.42°N where nitrate was present in micromolar concentrations. At the gyre station, in the control treatment sampled after 96 h, the *K. veneficum* species bin was predicted to be heterotrophic, and none of the nutrient amendments resulted in a change in its trophic predictions (Figure 3B). In the control treatment at 37°N, sampled at 96 h, the species bin was also predicted to be heterotrophic. The addition of iron (+Fe) resulted in 80% of predictions for heterotrophy and 20% for mixotrophy for the *K. veneficum* species bin. Given that MarPRISM has a potential error rate of 23%, the mixotrophy prediction in the +Fe treatment could be due to model error. However, the addition of nitrate and phosphate (+NP) resulted in a mix of heterotrophy and mixotrophy predictions that surpassed the potential error rate (Figure 3B). At 41.42°N, the *K. veneficum* species bin received mixotrophy predictions in the control treatment and when iron was added in low (+LoFe) and high amounts (+HiFe) (Figure 3B). The combined addition of nitrate, phosphate, and high amounts of iron (+NPFe) resulted in a mix of mixotrophy and phototrophy predictions; however, only 17% of predictions were for phototrophy, which could be due to model error (Figure 3B). The *P. beii* species bin showed the opposite pattern to the *K. veneficum* species bin across the surface transects, shifting from phototrophy to mixotrophy to heterotrophy predictions with increasing latitude (Figure 3A). Fewer nutrient amendment samples could be analyzed for the *P. beii* species bin due to fewer samples meeting the 70% CTG recovery cutoff, and more predictions being excluded for being split between phototrophy and heterotrophy, yet the general trend for this species bin was also different from that of the *K. veneficum* species bin in response to experimental nutrient amendment. In the gyre incubations, the *P. beii* species bin shifted from phototrophy predictions to a mix of phototrophy and mixotrophy predictions with the combined addition of low amounts of nitrate and phosphate (+LoNP) (Figure 3B). At 37°N, trophic predictions were not possible for the *P. beii* species bin in the control treatment, although the *in situ* predictions for this species bin at this site were all mixotrophic (Figures 3A, B). The addition of iron (+Fe) and macronutrients (+NP) both resulted in a majority of phototrophy predictions and one mixotrophy prediction whereas the addition of all three nutrients in combination (+NPFe) resulted in a majority of mixotrophy predictions (Figure 3B). At 41.42°N, the control treatment and addition of low amounts of iron (+LoFe) resulted in all phototrophy predictions (Figure 3B). When high amounts of iron were added (+HiFe), predictions for the *P. beii* species bin shifted from all phototrophic to all heterotrophic (Figure 3B). In the gyre, experimental nitrate amendment affected the trophic mode of the *P. beii* species bin, while in the transition zone, both nitrate and iron amendment exerted control over the trophic mode of the *K. veneficum* and *P. beii* species bins. The trophic shifts for these species bins in response to experimental nitrate amendment mirrored their trophic shifts from the gyre to the transition zone with increasing nitrate availability.

**Figure 4:**
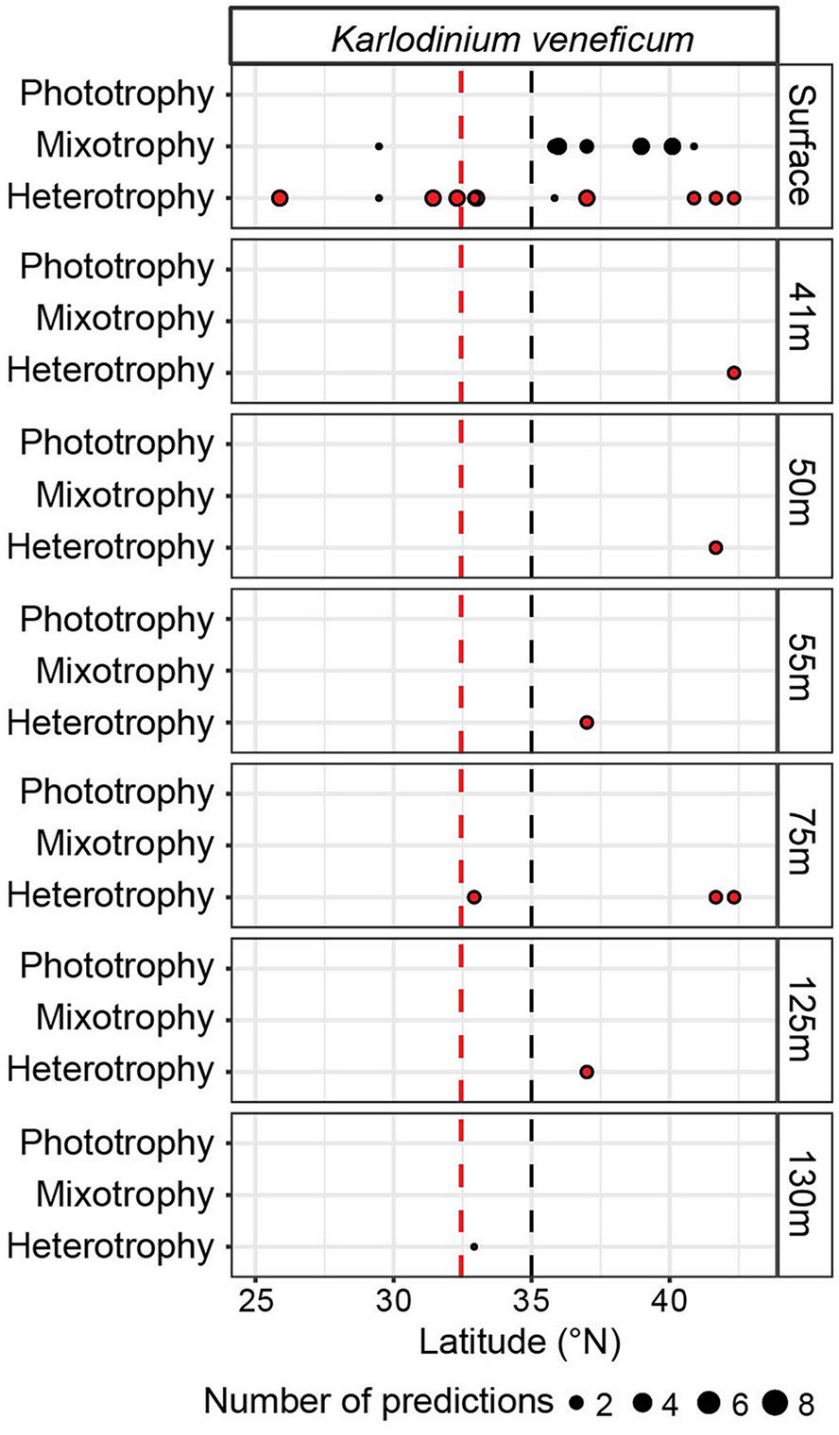
*In situ* trophic predictions across G3 upper-ocean depths for the *Karlodinium veneficum* species bin. Trophic predictions for the *K. veneficum* species bin throughout the G3 surface transect and G3 depth profiles were summed across replicates and size fractions. *K. veneficum* was the only species bin with mixotrophic capabilities that had enough predictions to analyze for the G3 depth profiles. Red dashed line: location of salinity isohaline (34.82); black dashed line: location of transition zone chlorophyll front (0.15 mg m^−3^ chlorophyll).

At four sites (32.92, 37, 41.67, and 42.33°N) on G3, we investigated trophic predictions across depth (down to 130 m), a transition across which both nutrient and light availability vary. *K. veneficum* was the only species bin determined to have mixotrophic capabilities with sufficient trophic predictions across the depth profiles to examine. At the surface, at 7 and 15 m, the *K. veneficum* species bin received heterotrophy predictions in the gyre compared to a mix of heterotrophy and mixotrophy predictions in the transition zone (Figure 4). In contrast, at depths from 41 to 130 m, spanning within and below the euphotic zone, only heterotrophy was predicted, suggesting that heterotrophy was more prevalent for this species bin under low light (Figure 4).

### Abundance of species bins across the surface ocean varied with trophic capabilities

**Figure 5:**
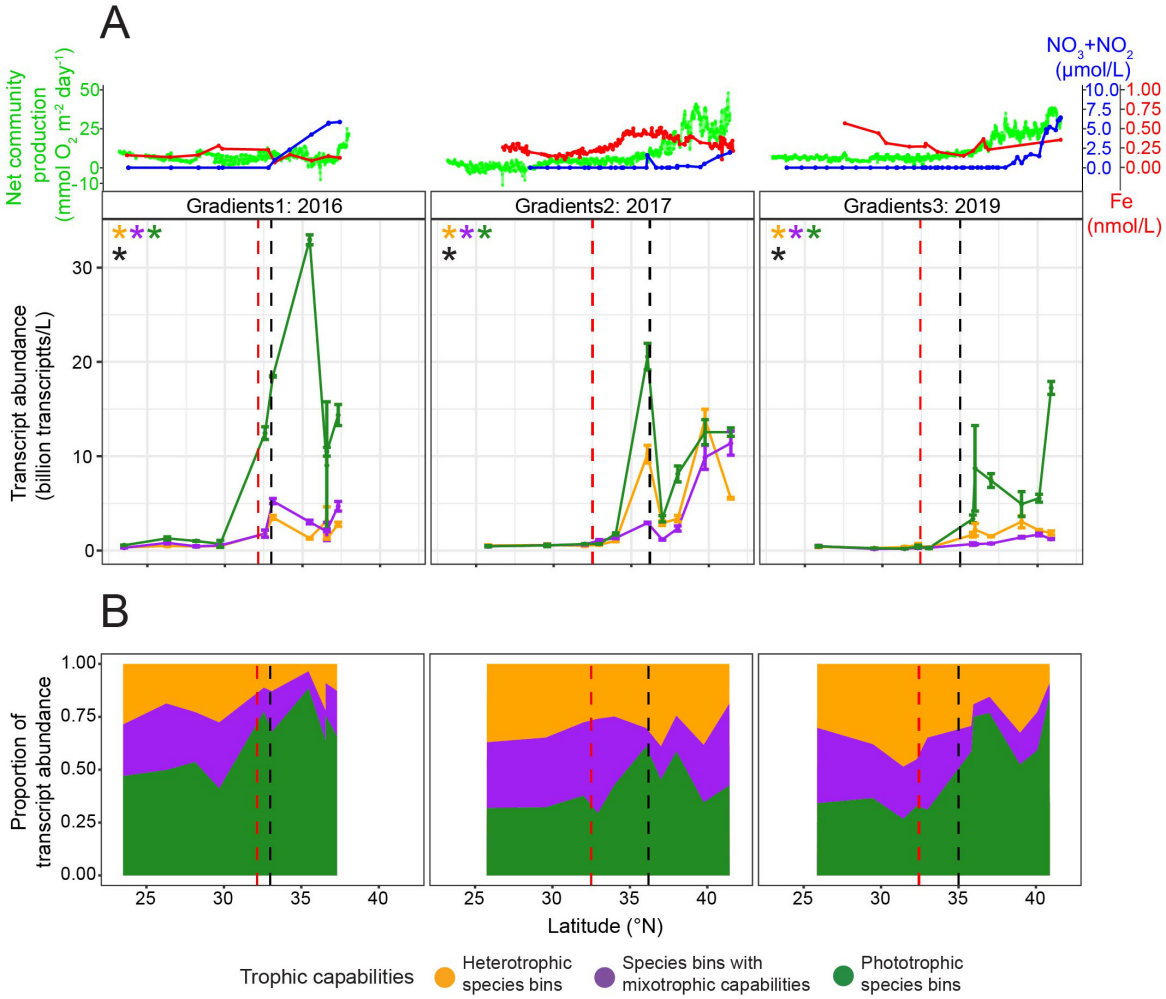
Transcript abundance across the G1–G3 surface transects for the 28 species bins for which trophic predictions were possible. **(A)** Top panel: surface net community production (green, mmol O_2_ m^−2^ day^−1^), nitrate/nitrite (blue, μmol/L), and dissolved iron concentrations (red, nmol/L) across latitude. Lower panel: Average transcript abundance of species bins by trophic group across surface waters. Transcript abundance was summed across five heterotrophic species bins (orange), eight species bins with mixotrophic capabilities (purple), and 15 phototrophic species bins (green), then averaged across replicates. Transcripts per liter for dinoflagellate species bins were corrected by dividing by 6.4 (Coesel et al., 2025). Error bars represent standard error. An asterisk signifies a significant difference (*p*-value ≤ 0.05) in total transcript abundance for the respective trophic group (asterisk color) across latitude, as determined by generalized additive models (GAMs). A significant difference in the relationship between transcript abundance and latitude across trophic groups (ANOVA on GAMs to determine significance of trophic group as a predictor, *p*-value ≤ 0.05) is marked with a black asterisk. **(B)** Proportion of transcript abundance by trophic group. The total transcript abundance for each trophic group was averaged across replicates, then divided by the total transcript abundance of the 28 species bins at each latitude. Red dashed line: location of salinity isohaline (34.82); black dashed line: location of transition zone chlorophyll front (0.15 mg m^−3^ chlorophyll).

For the 28 species bins with trophic predictions, we examined whether species bins with mixotrophic capabilities and trophic specialists were differentially distributed across the North Pacific surface ocean by quantifying transcript abundance (Figure 2). We hypothesized that the abundance of species bins with different trophic capabilities would differ in response to nutrient availability. Poly(A) sequencing, which was used to generate the eukaryotic metatranscriptomes in this study, introduces bias in transcript representation (Viscardi and Arribere, 2022), which could influence the transcript abundance of different taxa. However, Coesel et al. (2025) demonstrated—using several of the same datasets analyzed here—that poly(A)-selected transcript abundance correlates with estimated carbon biomass across broad taxonomic groups, with dinoflagellates producing ~6.4 times more transcripts per unit carbon biomass than other eukaryotes. After accounting for this offset, our transcript data can be interpreted as a semi-quantitative proxy for comparing biomass across taxa. During all three cruises, there was a significant difference in the relationship between total transcript abundance and latitude across the eight species bins with mixotrophic capabilities, 15 phototrophic species bins, and five heterotrophic species bins (ANOVAs on GAMs to determine significance of trophic group as a predictor, *p*-values ≤ 0.05) (Figure 5A). In the oligotrophic gyre, the three trophic groups of species bins had similarly low transcript abundance (ANOVAs on GAMs to determine significance of trophic group as a predictor at latitudes south of salinity isohaline (34.82), *p*-values > 0.05), making up relatively equal proportions of transcript abundance (Figure 5). Across the transition zone, nutrient availability and the transcript abundance of species bins with different trophic capabilities varied between the three cruises. Nitrate availability increased with latitude during all three cruises but reached higher concentrations in the transition zone during G1 and G3 than G2, while dissolved iron concentrations and net community production were higher in the transition zone during G2 and G3 than G1 (Figure 5A). All three trophic groups significantly increased (GAMs, *p*-values ≤ 0.05) in total transcript abundance with latitude, but phototrophic species bins increased to a greater extent than the other trophic groups during all three cruises (GAMs, predicted transcript abundance ranges) (Figure 5A). The total transcript abundance of the phototrophic species bins followed a similar pattern to nitrate availability along the transects (Figure 5A). In the transition zone during all three cruises, there was a significant difference in transcript abundance across the three trophic groups (ANOVAs on GAMs to determine significance of trophic group as a predictor at latitudes north of salinity isohaline (34.82), *p*-values ≤ 0.05), and the phototrophic species bins reached the highest total transcript abundances (Figure 5A). This led the phototrophic specialists to comprise the majority of transcript abundance of the species bins of interest in the transition zone during G1 and G3 (Figure 5B). In contrast, during G2 when nitrate concentrations were lower in the transition zone, the three trophic groups represented similar proportions of transcript abundance in the transition zone. This was due to an enhanced increase in the total transcript abundance with latitude of both the species bins with mixotrophic capabilities and heterotrophic species bins during G2 compared to G1 and G3 (GAMs, predicted transcript abundance ranges) (Figure 5).

Of the 28 species bins with trophic predictions, a few species bins had particularly high transcript abundance in the surface ocean. Transcript abundance of the species bins with mixotrophic capabilities was dominated by the species bins most closely related to *Chrysochromulina* sp. KB-HA01, *K. veneficum*, and *P. beii* (Supplementary Figure S9). Of the phototrophic species bins, the *P. calceolata* species bin and, to a lesser extent, the *B. prasinos* species bin had the highest transcript abundance (Supplementary Figure S9). During G1 and G3, the *P. calceolata* species bin had particularly high transcript abundance across much of the transition zone (Supplementary Figure S9). In contrast, during G2, the *P. calceolata* species bin had high transcript abundance at a single location, near the transition zone chlorophyll front (Supplementary Figure S9), which contributed to the observed rise and fall in total transcript abundance of phototrophic species bins across the transition zone (Figure 5A). The species bin most closely related to *O. trifallax* made up a large proportion of the transcript abundance of the heterotrophic species bins (Supplementary Figure S9).

**Figure 6:**
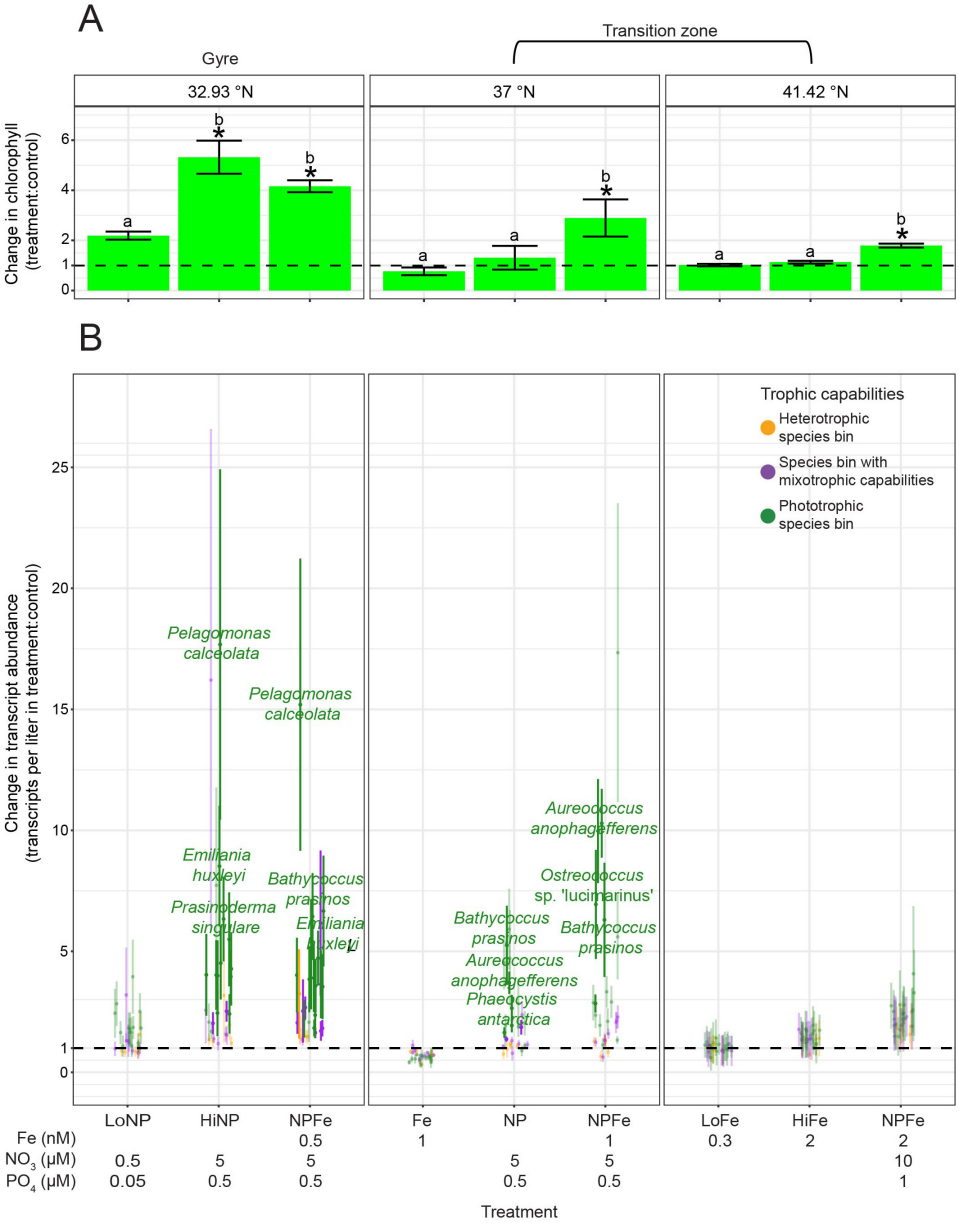
Change in chlorophyll *a* concentration, and change in transcript abundance for the 28 species bins for which trophic predictions were possible across G2 nutrient amendment incubations. **(A)** Change in average chlorophyll *a* concentration between control (no nutrient amendment) and nutrient-amended treatments after 96 h. Different ratios of iron, nitrate, and phosphate were added to the surface water community collected from 15 m at 32.93, 37, and 41.42°N during G2. The control and nutrient-amended treatments were sampled after 96 h. Asterisks indicate that the chlorophyll *a* concentration was significantly different between the control and nutrient amendment (ANOVA followed by *post-hoc* Tukey test, *p*-value ≤ 0.05). Different letters signify that the chlorophyll *a* concentration was significantly different between treatments, grouped by station (ANOVA followed by *post-hoc* Tukey test, *p*-value ≤ 0.05). **(B)** Average change in transcripts per liter for five heterotrophic species bins (orange), eight species bin with mixotrophic capabilities (purple), and 15 phototrophic species bins (green) between the control and nutrient-amended treatments after 96 h. Transcripts per liter for dinoflagellate species bins were corrected by dividing by 6.4 (Coesel et al., 2025). Species bins are marked with an opaque circle (rather than a circle with high transparency) if their change in transcript abundance was significantly greater in the nutrient amendment than the control (one-tailed *t*-test, multiple hypothesis testing correction grouped by latitude and treatment using Benjamini–Hochberg method, adjusted *p*-value ≤ 0.05). Species bins with significantly higher transcript abundance in the nutrient amendment than the control, and one of the three highest changes in transcript abundance for the respective latitude and treatment, are taxonomically labeled. Error bars represent the standard error of the change in transcript abundance in the nutrient amendment vs. the control. Dashed lines at one on the *y*-axes represent no change between the control and nutrient amendment.

To identify the limiting nutrients across the North Pacific surface ocean and disentangle the effects of nutrient availability on the transcript abundance of species bins with mixotrophic capabilities compared to phototrophic and heterotrophic specialists, we again analyzed the nutrient amendment incubations of the surface water community conducted during G2. At the gyre station at 32.93°N, after 96 h, the combined addition of 0.5 μM nitrate and 0.05 μM phosphate (+LoNP) did not result in a significant difference (*post-hoc* Tukey test, *p*-value = 0.14) in chlorophyll *a* or significant increases (one-tailed *t*-tests, adjusted *p*-values > 0.05) in the transcript abundance of any of the 28 species bins (Figure 6). The addition of an order of magnitude more nitrate and phosphate, with or without 0.5 nM iron (+HiNP or +NPFe), resulted in >4-fold significant increases (*post-hoc* Tukey tests, *p*-values ≤ 0.05) in chlorophyll *a* and significant increases (one-tailed *t*-tests, adjusted *p*-values ≤ 0.05) in the transcript abundance of 12 and 14 of the phototrophic species bins, respectively (Figure 6). There was no significant difference (*post-hoc* Tukey test, *p*-value = 0.16) in chlorophyll *a* between the +HiNP and +NPFe treatments (Figure 6A). The *P. calceolata, Emiliania huxleyi*, and *B. prasinos* species bins had >5-fold significant increases in transcript abundance in response to both the +HiNP and +NPFe treatments, with the *P. calceolata* species bin increasing over 17- and 15-fold, respectively (Figure 6B). While the phototrophic species bins had the largest increases in transcript abundance, there were also significant increases (one-tailed *t*-tests, adjusted *p*-values ≤ 0.05) in the transcript abundance of two species bins with mixotrophic capabilities in the +HiNP treatment and seven species bins with mixotrophic capabilities and one heterotrophic species bin in the +NPFe treatment (Figure 6B). At 37°N, the addition of 1 nM iron (+Fe) did not result in a significant difference in chlorophyll a (*post-hoc* Tukey test, *p*-value = 0.97) or significant increases (one-tailed *t*-tests, adjusted *p*-values > 0.05) in the transcript abundance of any of the target species bins (Figure 6). The addition of 5 μM nitrate and 0.5 μM phosphate (+NP) did not result in a significant difference in chlorophyll *a* (*post-hoc* Tukey test, *p*-value = 0.94) but did lead to significant increases (one-tailed *t*-tests, adjusted *p*-values ≤ 0.05) in the transcript abundance of five phototrophic species, from ~1.5- to 5.5-fold, and of two species bins with mixotrophic capabilities, from ~1.5- to 2-fold (Figure 6). The addition of 1 nM iron with nitrate and phosphate (+NPFe) led to a significant increase (*post-hoc* Tukey test, *p*-value = 3.1e-02) of ~3-fold in chlorophyll *a* and significant increases (one-tailed *t*-tests, adjusted *p*-values ≤ 0.05) in the transcript abundance of five phototrophic species bins, from ~3- to 10.5-fold, with the species bins corresponding to *Aureococcus anophagefferens, Ostreococcus* sp. ‘lucimarinus,' *B. prasinos*, and *P. calceolata* significantly increasing more than 6-fold (Figure 6). At 41.42°N, the addition of small (0.3 nM, +LoFe) and large amounts of iron (2 nM, +HiFe) did not result in significant differences (*post-hoc* Tukey tests, *p*-values > 0.05) in chlorophyll *a* or significant increases (one-tailed *t*-tests, adjusted *p*-values > 0.05) in the transcript abundance of any of the target species bins (Figure 6). The addition of 2 nM of iron with nitrate and phosphate (+NPFe) resulted in a significant (*post-hoc* Tukey test, *p*-value = 1.5e-06) yet modest— < 2-fold—increase in chlorophyll *a* but no significant increases (one-tailed *t*-tests, adjusted *p*-values > 0.05) in the transcript abundance any of the target species bins (Figure 6). These nutrient-amended incubations showed that at 32.93°N in the gyre, the surface water protist community was limited by nitrate while at 37 and 41.42°N in the transition zone, protists were co-limited by nitrate and iron.

### Abundance of species bins from 15 to 130 m varied with trophic capabilities

**Figure 7:**
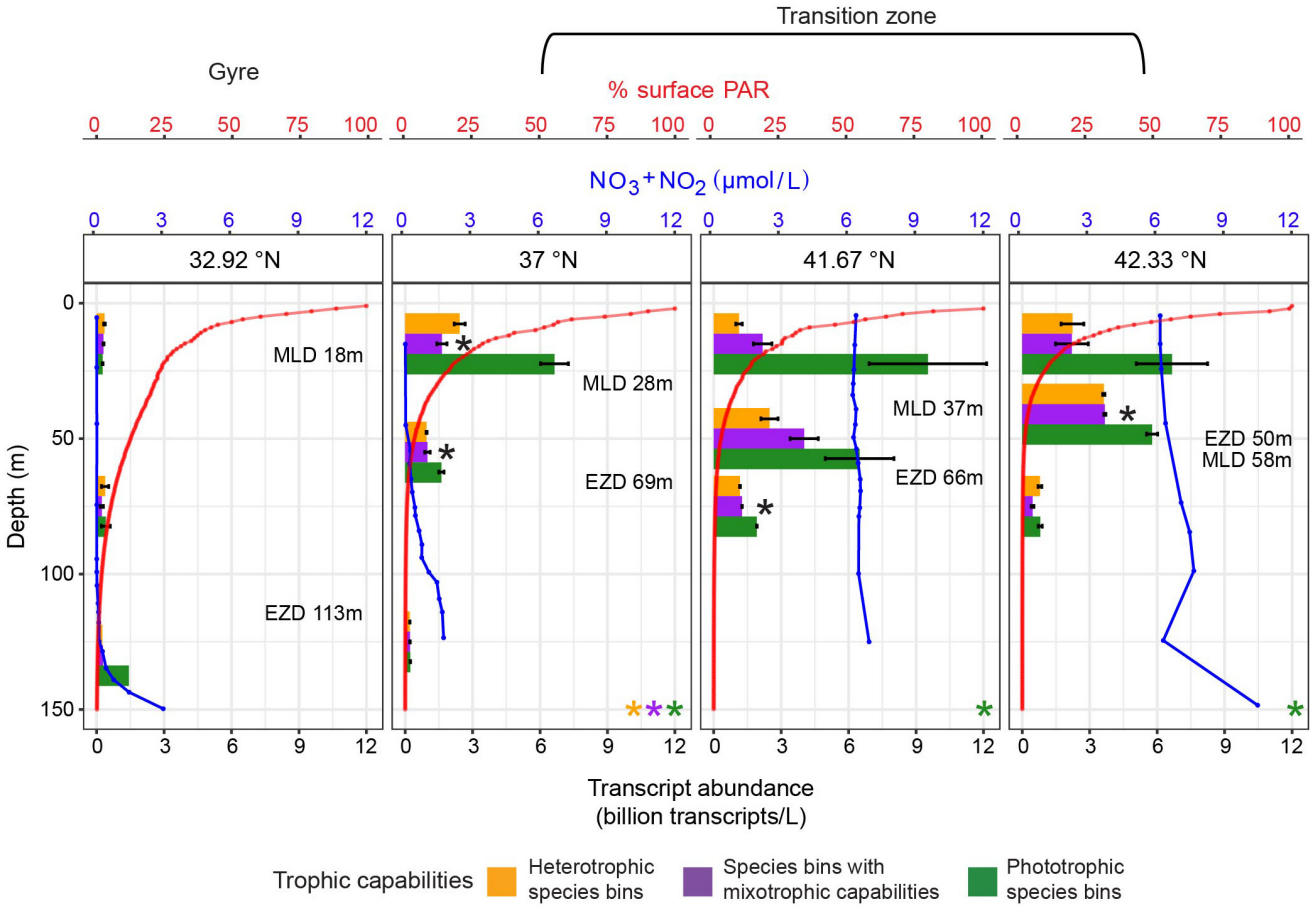
Transcript abundance throughout the G3 depth profiles for the 28 species bins for which trophic predictions were possible. Transcript abundance was summed across five heterotrophic species bins (orange), eight species bin with mixotrophic capabilities (purple), and 15 phototrophic species bins (green), then averaged across replicates. Transcripts per liter for dinoflagellate species bins were corrected by dividing by 6.4 (Coesel et al., 2025). Two replicates were collected at three depths for each depth profile except 130 m at 32.92°N, which had just one replicate. Error bars represent standard error. A black asterisk signifies a significant difference (*p*-value ≤ 0.05) in total transcript abundance between the trophic groups at the respective depth, as determined by one-way ANOVA. An asterisk at the bottom of the plot signifies a significant difference (*p*-value ≤ 0.05) in total transcript abundance for the respective trophic group (asterisk color) between the three depths, as determined by one-way ANOVA. Nitrate/nitrite concentrations (blue, μmol/L) and percentage surface photosynthetically active radiation (PAR, red) are plotted throughout depth. The approximate mixed layer depth (MLD) and euphotic zone depth (EZD, 1% surface PAR) are labeled for each depth profile.

We evaluated transcript abundance across depth profiles for the 28 species bins under the hypothesis that across depth, both light and nitrate availability would affect the transcript abundance of species bins with different trophic capabilities. The metatranscriptomes were collected at three depths at each of four latitudes. At 32.92°N, nitrate concentrations remained low until ~99 m, after which they increased, and the euphotic zone (1% surface PAR) extended to 113 m (Figure 7). At this latitude, transcript abundance was low at both 15 and 75 m, with no significant difference (one-way ANOVAs, *p*-values > 0.05) observed between the three trophic groups (Figure 7). At 130 m (0.42% surface PAR), the transcript abundance of species bins with mixotrophic capabilities and heterotrophic species bins was low and comparable, while phototrophic species bins exhibited a maximum in transcript abundance, surpassing the other trophic groups; only one metatranscriptomic replicate for 130 m prevented significance testing (Figure 7). At 37°N, the euphotic zone extended to 69 m, while nitrate concentrations increased with depth, showing a steeper rise below 45 m (Figure 7). At the two most shallow depths sampled at this latitude (15 and 55 m), transcript abundance of the phototrophic species bins was significantly greater (one-way ANOVAs, *p*-values ≤ 0.05) than the other trophic groups (Figure 7). Transcript abundance of each trophic group significantly decreased (one-way ANOVAs, *p*-values ≤ 0.05) with depth, leading to a low total transcript abundance, and no significant difference (one-way ANOVA, *p*-value = 0.40) in transcript abundance between trophic groups at 125 m (0.08% surface PAR) (Figure 7). At 41.67°N, the euphotic zone depth was 66 m, and nitrate concentrations were high throughout the sampled depths (Figure 7). Transcript abundance of the phototrophic species bins decreased significantly (one-way ANOVA, *p*-value = 3.1e-02) with depth, but was still significantly higher (one-way ANOVA, *p*-value = 6.2e-05) than the other trophic groups at 75 m (0.59% surface PAR) (Figure 7). At 42.33°N, the euphotic zone extended to 50 m, and nitrate concentrations remained high throughout the sampled depths (Figure 7). The transcript abundance of the phototrophic species bins decreased significantly (one-way ANOVA, *p*-value = 1.5e-02) with depth (Figure 7). The phototrophic species bins had significantly higher (one-way ANOVA, *p*-value = 3.2e-03) transcript abundance than the other trophic groups at 41 m, while at 75 m (0.17% surface PAR), the three trophic groups did not significantly differ (one-way ANOVA, *p*-value = 8.0e-02) in transcript abundance.

A number of the species bins with high transcript abundance across the surface transects had high transcript abundance throughout the depth profiles as well. Of the species bins with mixotrophic capabilities, the *K. veneficum* species bin had the highest transcript abundance below 15 m (Supplementary Figure S10). Of the phototrophic species bins, the *P. calceolata* species bin and to a lesser extent, the *B. prasinos, Phaeocystis antarctica*, and *T. pacifica* species bins had high transcript abundance below 15 m (Supplementary Figure S10). The *P. calceolata* species bin made up a large portion of the total transcript abundance of the target species bins at 130 m in the gyre (Supplementary Figure S10). The species bins corresponding to *O. trifallax* and Stramenopiles sp. TOSAG23-2 were the heterotrophic species bins with the highest transcript abundance below 15 m (Supplementary Figure S10).

## Discussion

We set out to expand the diversity and number of metatranscriptomic samples analyzed for the *in situ* trophic mode of marine protist species, incorporating newly available metatranscriptomes from the North Pacific Ocean, which span diel experiments, surface transects, depth profiles (to 130 m), and nutrient-amended incubations. The study region, located at the subtropical gyre to the subtropical–subpolar transition zone, features gradients in nutrients, light, temperature, prey availability, and plankton community structure, thus serving as a natural laboratory to examine the *in situ* trophic mode and abundance of marine protist species.

### Refined model to predict the *in situ* trophic mode of marine protist species

MarPRISM performed well overall and for each trophic mode despite the MMETSP-derived transcriptomes not representing a gold-standard dataset for training a model to predict *in situ* trophic mode. Trophic mode labels were assigned based on the trophic capabilities of taxa known from the literature and culture conditions (e.g., light vs. dark, presence vs. absence of bacteria) rather than through direct measurement of trophic mode. The assignment of trophic mode labels involved several assumptions: (1) that the specific strain used for sequencing possesses mixotrophic capabilities if it belongs to a taxon with members documented to have mixotrophic capabilities, and (2) that species with mixotrophic capabilities cultured in the light and with prey were actively engaged in phagotrophy, rather than solely photosynthesis, the latter of which could have been more favorable in nutrient-rich culture. In addition, some species may have been mischaracterized in the literature—for example, erroneously identified as having mixotrophic capabilities. Despite these potential sources of error, model performance remained high overall and for each trophic mode. Potential errors in the trophic mode labeling of individual transcriptomes likely had a limited impact on model performance. One specific example of potential error in the training dataset labels involves *Micromonas*, a genus for which the literature is divided regarding its mixotrophic capabilities (e.g., McKie-Krisberg et al., 2018; Jimenez et al., 2021). However, model performance was not sensitive to the trophic mode labeling of *Micromonas* strains in the training dataset. Furthermore, the ability of MarPRISM to perform well when trained on just 75% of its training dataset suggests that the model captured generalizable transcriptional patterns across protist species, such that accurate predictions can be made even for species that are not closely related to those in the training dataset. Supporting this, the transcriptome from a culture of the non-photosynthetic diatom *Nitzschia* sp. Nitz4 was correctly predicted by MarPRISM to be heterotrophic, despite all diatom transcriptomes in the training dataset being phototrophic.

Despite similar accuracy to the previous version of the model Lambert et al., (2022), MarPRISM made trophic predictions using a substantially reduced set of feature Pfams—183 Pfams compared to the original 1,046. This refinement enabled more precise identification of the Pfams with transcriptional patterns that influence protist trophic mode. It is possible that the expression of contigs unannotated by the Pfam database could aid trophic predictions; however, we were able to achieve high accuracy without including functionally unannotated contigs. We compared feature selection and model performance between TPM-based expression values and binarized data (where TPM > 0 was set to 1) to assess whether the features used by MarPRISM reflected nuanced differential expression vs. simple presence or absence of expression. The binarized model required more feature Pfams (245 vs. 183) to achieve comparable performance, suggesting that continuous TPM values carry additional information useful for trophic predictions. Unexpectedly, only 37 feature Pfams overlapped between the two models. Because it uses the full information contained in continuous expression data and relies on fewer features, we selected MarPRISM over the binary-based model. Importantly, the lack of a significant difference in model performance between the two input types does not imply applicability of a version of the model to genome-derived data as the binarized expression of feature Pfams was still based on transcriptomes and thus reflects gene expression rather than the genomic presence or absence of Pfams.

Pfams utilized as features by MarPRISM provide insights into the moleccular functions associated with different trophic strategies. As expected, photosynthesis-related Pfams were indicative of phototrophic and mixotrophic transcriptomes. Motility-related Pfams were characteristic of mixotrophic and heterotrophic transcriptomes, suggesting the importance of motility for protists that ingest prey. One feature Pfam characteristic of mixotrophic and heterotrophic transcriptomes was associated with phagocytosis of apoptotic cells in mammals (Gumienny et al., 2001), and thus may play a role in the phagocytosis of prey by protists. The inability to directly link feature Pfams to prey ingestion could stem from limited functional annotations for protists or from overlaps between Pfams involved in motility and prey ingestion, and carbon metabolism and prey ingestion. While we could not discern links between some of the feature Pfams and trophic mode, their transcriptional patterns were still essential to model performance. This demonstrates that the trophic mode of marine protist species cannot be predicted by the expression of specific genes alone—especially genes chosen *a priori*—but rather, is governed by complex, interacting transcriptional patterns. Further functional characterization of the 183 feature Pfams through targeted laboratory experiments will enable greater insight into these patterns.

### Trophic capabilities of species determined from aggregated trophic predictions

Our classifications of species bins' trophic capabilities, determined from trophic predictions aggregated across samples, were largely in line with the known trophic abilities of their closest relatives in culture. Differences between our classifications and laboratory culture observations may highlight ecological dynamics specific to the ocean that are not replicated in controlled settings. For example, laboratory-cultured protists may lose their mixotrophic capabilities after being grown in light and high nutrient conditions for long periods of time (Flynn et al., 2019; Blossom and Hansen, 2020). Our trophic predictions also provide insights into species that remain understudied in laboratory research. The taxonomic annotations in our study rely on available reference sequences, which is an important limitation, given that even closely related protist species can differ in trophic strategy (Lie et al., 2018). As a result, species bins predicted to have mixotrophic capabilities could have originated from closely related taxa with different trophic modes. Additionally, our classifications of species bins' trophic capabilities may differ from the known trophic capabilities of their closest relatives in the literature because the actual taxonomy of these species bins may differ from that of their taxonomic annotation. For example, the species bin identified as *T. pacifica*, which we predicted to be a phototrophic specialist, may differ in taxonomy and/or behavior from the only *Triparma* strain, identified at the genus level, shown to ingest prey in culture (Li et al., 2022). In contrast to the *T. pacifica* species bin, which only received phototrophy predictions, we found its close relative, the species bin identified as *Triparma* sp. 1657, to have mixotrophic capabilities. Similarly, little is known about the trophic capabilities of *P. antarctica*. The Burns et al. (2018) model, trained to predict trophic capabilities from the presence or absence of genes, predicted three *P. antarctica* strains to have a high likelihood of mixotrophy and another strain to have an intermediate probability of mixotrophy (Koppelle et al., 2022), but phagocytosis by this species has not been directly observed. In contrast to Burns et al. (2018), we found the species bin most closely related to *P. antarctica* to be a phototrophic specialist. The trophic capabilities of *Dictyocha speculum*, which our predictions suggested to be strictly phototrophic, are also uncertain in the literature. One study found that *D. speculum* did not uptake fluorescently labeled bacteria (Havskum and Riemann, 1996), while another found operational taxonomic units similar to *Dictyocha* to be putatively mixotrophic (Gast et al., 2018). Although limited experiments have not yet observed *Azadinium* to ingest prey (Tillmann et al., 2014), MarPRISM's trophic predictions indicated that the *A. spinosum* species bin had mixotrophic capabilities. Our results provide insights into protist species like *T. pacifica, P. antarctica, D. speculum*, and *A. spinosum* whose trophic capabilities are not well-studied. It is also possible that for some of the species bins, we did not sample the marine conditions that induce other trophic modes. This would be most likely for the species bins identified as *D. acuminata, G. catenatum* GC744, *Calcidiscus leptoporus, P. antarctica, Phaeocystis globosa*, and *P. polylepis*, as few trophic predictions were possible for these species bins. Additionally, we may not have detected mixotrophic capabilities for species bins that utilize phagocytosis at low rates, as MarPRISM likely reflects the dominant trophic mode of the population. This may explain why the species bins most closely related to *C. leptoporus* and *E. huxleyi* were classified as phototrophic specialists, despite previous observations of infrequent and sporadic prey ingestion by these species in laboratory cultures: (Avrahami and Frada 2020) found < 1% of cultured cells from both species to contain prey, with no significant differences observed between nutrient-replete and nutrient-limited conditions. These findings suggest that MarPRISM may not detect heterotrophy and mixotrophy when phagotrophy occurs at a low frequency within a population. MarPRISM's detection of only the dominant trophic mode of cells may explain why species bins with closest relatives identified in the literature as constitutive mixotrophs—*K. veneficum, P. beii, P. minimum, S. trochoidea*, and *T. fusus*—were sometimes predicted to be heterotrophic. Although these taxa retain plastids and cannot discard them, they may have been photosynthesizing at very low rates during instances in which they were predicted to be heterotrophic. The fact that MarPRISM's predictions aligned with the known trophic capabilities of the closest relative in the literature for the majority of species bins supports the model's application to field samples. Instances where MarPRISM's predictions diverge from literature expectations highlight the model's potential to provide insights into understudied species or to uncover trophic capabilities present in nature but absent in laboratory cultures.

### Mixotrophs showed species-specific trophic responses to nutrient and light availability

The species bin identified as *K. veneficum* exhibited the clearest trophic shifts across environmental gradients; however, it is possible such patterns could reflect different, but closely related, strains grouped under the same species bin. Because contigs and their mapped transcripts were aggregated based on the closest known taxonomic relative in the reference database, species bins may represent distinct taxa, each with potentially different distributions and trophic strategies. In the surface ocean, from the gyre to the transition zone, the *K. veneficum* species bin transitioned from heterotrophy to a mix of heterotrophy and mixotrophy predictions. With the experimental amendment of nitrate and iron at transition zone stations, the *K. veneficum* species bin displayed a shift from heterotrophy to more mixotrophy and phototrophy predictions. These results align with what is known about *K. veneficum* (previously known as *Gyrodinium galatheanum*) from laboratory studies. The species can grow as a phototroph in the absence of a food source (Li et al., 1999), and some strains can sustain heterotrophic growth for months (Calbet et al., 2011). Phagocytosis serves as a strategy for *K. veneficum* to supplement nitrate and phosphate to carry out photosynthetic carbon assimilation (Li et al., 2000), consistent with our observations of heterotrophy predictions in the oligotrophic gyre and more mixotrophy predictions in the transition zone. Trophic predictions across the nutrient amendment incubations showed that the *K. veneficum* species bin may also use phagocytosis to acquire iron. Additionally, the species bin identified as *K. veneficum* may compensate for reduced light availability at depth by increasing prey ingestion to acquire carbon, as exclusively heterotrophy predictions were observed below 15 m down to 130 m for the species bin. Cohen et al. (2021) hypothesized that across the tropical Pacific, autotrophic/mixotrophic Kareniaceae (the family containing *K. veneficum*) species reside at euphotic depths in contrast to heterotrophic Kareniaceae species that reside in the mesopelagic zone. This hypothesis may hold true, but we add to the complexity by predicting a trophic shift within one Kareniaceae species—the species bin with the closest relative *K. veneficum*—across depths of the upper 130 m of the ocean, including within and below the euphotic zone.

Protist species bins with mixotrophic capabilities showed varied trophic responses across the North Pacific Ocean. In contrast to the *K. veneficum* species bin, within the surface ocean, we found three species bins, *Chrysochromulina* sp. KB-HA01, *Triparma* sp. 1657, and *P. beii*, to shift from phototrophy toward mixotrophy or heterotrophy from the gyre to the transition zone. Very little is known about the factors that regulate the trophic mode of these taxa. Only recently were *P. beii* and a strain of *Triparma* shown to ingest prey (Li et al., 2022). While some species of *Chrysochromulina* increase prey ingestion under nutrient limitation, there is a large spectrum of mixotrophic strategies across *Chrysochromulina* species (Jones et al., 1993). Few studies have investigated the balance of photosynthesis and grazing by mixotrophic protists in response to environmental changes within natural communities. However, laboratory experiments have demonstrated that mixotrophic protist species differ in which resources modulate their trophic mode. Some mixotrophic species increase grazing under nutrient limitation, while primarily phagotrophic species rely more on photosynthesis when prey is scarce (Rothhaupt, 1996). Other species enhance prey ingestion under low light to offset reduced photosynthetic carbon assimilation (Sibbald and Albright, 1991; Jones et al., 1993). This diversity in mixotrophic strategies may explain the species-specific variations in trophic shifts we observed across the North Pacific Ocean, which hosts changes in temperature and the availability of light, prey, and macro- and micronutrients.

Enhanced data availability and improved taxonomic annotations likely allowed us to identify the diversity of intraspecies trophic shifts across the North Pacific Ocean. Lambert et al. (2022) observed a shift toward phototrophy from the gyre to the transition zone in all three species bins they analyzed for trophic shifts across latitude: *Chrysochromulina rotalis, Chrysochromulina brevifilum*, and *Micromonas pusilla*. *C. rotalis, C. brevifilum*, and *M. pusilla* lacked sufficient coverage in our samples to receive trophic predictions. We used a more stringent coverage cutoff for species bins to receive a trophic prediction—at least 70% of CTGs expressed vs. at least 800 Pfams expressed—than Lambert et al. (2022), and we used a different database for taxonomic annotations—MarFERReT vs. a reference database created by Coesel et al. (2021). This led to different species bins passing the coverage threshold for trophic predictions between our study and Lambert et al. (2022). Our use of MarFERReT for improved taxonomic annotations, our analysis of a greater number of species bins with varied trophic predictions (eight vs. three), and our examination of surface transects spanning 3 years instead of one likely allowed us to capture a broader diversity of responses of mixotrophic species to environmental conditions than Lambert et al. (2022).

### Phototropic specialists dominated the protist community in regions with high nitrate availability

The transcript abundance patterns we observed support the hypothesis that an increase in nutrient availability under well-lit conditions favors phototrophic protists over protists with mixotrophic capabilities (Edwards et al., 2023). In the low nitrate conditions of the gyre surface ocean, total transcript abundance of the 28 target species bins was low and was not dominated by any particular trophic group. Nitrate availability increased with latitude across the G1–G3 surface transects, reaching the highest concentrations at the northern latitudes. This increase in nitrate availability was associated with the greatest increase in the transcript abundance of phototrophic species bins. Incubations showed that at 32.93°N, in the gyre, the surface community was nitrate-limited, and the addition of nitrate favored the phototrophic specialists, particularly *P. calceolata*. Of the target species bins, *P. calceolata* also increased the most in transcript abundance across the surface ocean from the gyre to transition zone. In the transition zone, at 37 and 41.42°N, incubations demonstrated that the surface community was co-limited by nitrate and iron, as has been previously found for the transition zone in spring (Hawco et al., 2025). The addition of macronutrients and iron favored phototrophic specialists at 37°N in the transition zone. Protist species specialized in phototrophy were best poised to take advantage of enhanced nitrate availability with increasing latitude across the surface ocean. Under high nitrate availability in the transition zone during G1 and G3, this led to phototrophic species bins dominating over the other protist trophic groups.

Different trends in nutrient availability and protist community composition across the surface ocean were observed during G2 compared to G1 and G3, likely due to nitrate-iron co-limitation and bloom seasonality. Dissolved iron concentrations were higher in the transition zone during G2 and G3 than in G1 (Park et al., 2023; Hawco et al., 2025). The differences in iron distributions across G1 (April to May 2016) and G2 (May to June 2017) were likely driven by seasonality in the deposition of natural and anthropogenic aerosols originating from Asia, which deliver soluble iron to the transition zone (Pinedo-González et al., 2020; Park et al., 2023). This seasonal iron supply allows the plankton community to bloom and draw down nitrate concentrations, as observed on G2, which occurred later in the seasonal cycle. G2 was distinct from G1 and G3 in that the species bins with mixotrophic capabilities and the heterotrophic species bins increased in transcript abundance with latitude to a greater extent. Given that net community production was high in the transition zone during G2, net community production was calculated based on O_2_/Ar (Juranek et al., 2020), and oxygen's residence time in the mixed layer is around 2 weeks, we likely captured protist community dynamics soon after nitrate was depleted. During this phase, heterotrophic specialists may thrive by feeding on bloom remnants. At the same time, protists with mixotrophic capabilities may gain an advantage either from having more to feed on or by competing more effectively with phototrophic specialists under lower nitrate conditions.

Species bins with mixotrophic capabilities comprised a greater share of the protist community in the nutrient-limited gyre and during a transient period of nutrient limitation in the transition zone. Species with mixotrophic capabilities likely help maintain steady, though moderate, primary production in the gyre, supporting year-round carbon export and energy transfer to higher trophic levels. While in the transition zone, species with mixotrophic capabilities may extend the duration of heightened primary production and associated carbon export and energy transfer to higher trophic levels further into the spring, beyond the main bloom.

### Light and nutrient availability shaped the protist community across the upper-ocean depths

Light and nitrate availability, in combination, shaped protist community composition from 15 to 130 m through effects on phototrophic specialists. In the subtropical gyre, phototrophic species bins reached their highest transcript abundance at 130 m, where they had higher transcript abundance than the other trophic groups. However, the availability of a single replicate at 130 m precluded significance testing. Depth within the gyre did not appear to affect the transcript abundance of heterotrophic specialists and species bins with mixotrophic capabilities. In the subtropical gyre, phototrophic specialists may have reached higher transcript abundance at 130 m than at the surface due to increased nitrate availability at depth and their ability to efficiently photosynthesize under low-light conditions. In contrast, mixotrophs must distribute their biomass and energy across multiple trophic functions, which may increase respiratory demand and reduce photosynthetic efficiency compared to phototrophic specialists (Raven, 1997; Berge et al., 2017). At the surface across the transition zone during G3, phototrophic specialists had higher transcript abundance than the other trophic groups. At the transition zone stations with depth profiles (37, 41.67, and 42.33°N), phototrophic specialists decreased in transcript abundance with depth. Nitrate concentrations were high in the transition zone during G3, which may have made it advantageous for phototrophic specialists to be at the surface, where there was greater light availability than at depth. As was observed in the gyre, the transcript abundance of heterotrophic specialists and species bins with mixotrophic capabilities was less sensitive to depth than the phototrophic specialists. In the transition zone, the phototrophic specialists often had higher transcript abundance than the other trophic groups at depths between 41 and 75 m. This may again reflect the ability of phototrophic specialists to more efficiently photosynthesize than mixotrophs under low-light conditions. The protist community was balanced across the three trophic groups at 125 m at 37°N, likely because only 0.08% of surface PAR was available at this depth. A low percentage of surface PAR (0.17%) may also explain why the protist community was balanced at 75 m at 42.33°N. Very low irradiances like these likely prevented the phototrophic specialists from reaching higher transcript abundances than the other trophic groups. As irradiance decreased and light attenuation throughout depth increased with latitude, phototrophic specialists became restricted to shallower depths.

### Conclusion

North Pacific protists displayed intraspecies trophic mode shifts across surface transects, upper-ocean depths, and in response to experimental amendment of nitrate and iron, with trophic shifts varying across species. These shifts may reflect true intraspecies flexibility or the presence of distinct, closely related strains grouped within the same species bin due to limited taxonomic resolution. At the surface, the protist community was nitrate-limited in the gyre and nitrate and iron co-limited in the transition zone. Nitrate availability emerged as a key factor driving the protist community from a balanced mix of species with mixotrophic capabilities, phototrophic specialists, and heterotrophic specialists in the gyre to a transition zone under high nitrate availability dominated by phototrophic specialists. Both nitrate and light availability influenced protist community composition across depth, down to 130 m, through effects on phototrophic specialists. Phototrophic specialists had high transcript abundance at 130 m in the subtropical gyre due to deep nitrate availability and at the surface in the transition zone where nitrate was abundant. In contrast, the transcript abundance of species bins with mixotrophic capabilities and heterotrophic specialists was relatively insensitive to depth, down to 130 m. These findings underscore the importance of metatranscriptomes and machine learning models such as MarPRISM in enhancing our understanding of the trophic capabilities, *in situ* activity, and abundance of protist species across diverse marine ecosystems.

## Data Availability

The original contributions presented in the study are publicly available. The data can be found at the NCBI database under accession numbers PRJNA1148215 and PRJNA690575. Code for model development, testing, and running can be found in the following GitHub repository: https://github.com/armbrustlab/MarPRISM. Additionally, this repository contains code for processing of metatranscriptomes.

## References

[B1] Alexander H. Hu S. K. Krinos A. I. Pachiadaki M. Tully B. J. Neely C. J.. (2023). Eukaryotic genomes from a global metagenomic data set illuminate trophic modes and biogeography of ocean plankton. mBio 14:e0167623. 10.1128/mbio.01676-23 37947402 PMC10746220

[B2] Ashkezari M. D. Hargen N. R. Denholtz M. Neang A. Burns T. C. Morales R. L.. (2021). Simons Collaborative Marine Atlas Project (Simons CMAP): an open-source portal to share, visualize, and analyze ocean data. Limnol. Oceanogr. Methods 19, 488–496. 10.1002/lom3.10439

[B3] Avrahami Y. Frada M. J. (2020). Detection of phagotrophy in the marine phytoplankton group of the coccolithophores (Calcihaptophycidae, Haptophyta) during nutrient-replete and phosphate-limited growth. J. Phycol. 56, 1103–1108. 10.1111/jpy.12997 32233088

[B4] Bateman A. Coi L. Durbin R. Finn R. D. Hollich V. Griffiths-Jones S.. (2004). The Pfam protein families database. Nucleic Acids Res. 32(suppl_1), D138–D141. 10.1093/nar/gkh121 14681378 PMC308855

[B5] Benoit G. Raguideau S. James R. Phillippy A. M. Chikhi R. Quince C. (2024). High-quality metagenome assembly from long accurate reads with metaMDBG. Nat. Biotechnol. 42, 1378–1383. 10.1038/s41587-023-01983-6 38168989 PMC11392814

[B6] Berge T. Chakraborty S. Hansen P. J. Andersen K. H. (2017). Modeling succession of key resource-harvesting traits of mixotrophic plankton. ISME J. 11, 212–223. 10.1038/ismej.2016.92 27482925 PMC5315484

[B7] Blossom H. E. Hansen P. J. (2020). The loss of mixotrophy in Alexandrium pseudogonyaulax: implications for trade-offs between toxicity, mucus trap production, and phagotrophy. Limnol. Oceanogr. 66, 528–542. 10.1002/lno.11621

[B8] Bock N. A. Charvet S. Burns J. Gyaltshen Y. Rozenberg A. Duhamel S.. (2021). Experimental identification and *in silico* prediction of bacterivory in green algae. ISME J. 15, 1987–2000. 10.1038/s41396-021-00899-w 33649548 PMC8245530

[B9] Bolger A. M. Lohse M. Usadel B. (2014). Trimmomatic: a flexible trimmer for Illumina sequence data. Bioinformatics 30, 2114–2120. 10.1093/bioinformatics/btu170 24695404 PMC4103590

[B10] Bray N. L. Pimentel H. Melsted P. Pachter L. (2016). Near-optimal probabilistic RNA-seq quantification. Nat. Biotechnol. 34, 525–527. 10.1038/nbt.3519 27043002

[B11] Buchfink B. Xie C. Huson D. H. (2015). Fast and sensitive protein alignment using DIAMOND. Nat. Methods 12, 59–60. 10.1038/nmeth.3176 25402007

[B12] Burns J. A. Pittis A. A. Kim E. (2018). Gene-based predictive models of trophic modes suggest Asgard archaea are not phagocytotic. Nat. Ecol. Evol. 2, 697–704. 10.1038/s41559-018-0477-7 29459706

[B13] Cain K. Ribalet F. Armbrust E. V. (2020a). Discrete Flow Cytometry From the Gradients 2016 Cruise Using a BD Influx Cell Sorter. Zenodo.

[B14] Cain K. Ribalet F. Armbrust E. V. (2020b). Discrete Flow cytometry From the Gradients 2017 Cruise Using a BD Influx Cell Sorter. Zenodo.

[B15] Cain K. Ribalet F. Armbrust E. V. (2020c). Discrete Flow Cytometry of Underway Samples From the Gradients 2019 Cruise Using a BD Influx Cell Sorter. Zenodo. 10.5281/zenodo.4085897

[B16] Calbet A. Bertos M. Fuentes-Grünewald C. Alacid E. Figueroa R. Renom B.. (2011). Intraspecific variability in *Karlodinium veneficum*: growth rates, mixotrophy, and lipid composition. Harmful Algae 10, 654–667. 10.1016/j.hal.2011.05.001

[B17] Chang J. Carpenter E. J. (1994). Inclusion bodies in several species of *Ceratium* Schrank (Dinophyceae) from Caribbean Sea examined with DNA-specific staining. J. Plankton Res. 16, 197–202. 10.1093/plankt/16.2.197

[B18] Charvet S. Bock N. Kim E. Duhamel S. (2024). Transcriptomics reveal a unique phago-mixotrophic response to low nutrient concentrations in the prasinophyte *Pterosperma cristatum*. ISME Commun. 4:ycae083. 10.1093/ismeco/ycae083 38957873 PMC11217555

[B19] Chen T. Guestrin C. (2016). “XGBoost: a scalable tree boosting system,” in Proceedings of the 22nd ACM SIGKDD International Conference on Knowledge Discovery and Data Mining, 785–794. 10.1145/2939672.2939785

[B20] Coesel S. N. Durham B. P. Groussman R. D. Hu S. K. Caron D. A. Morales R. L.. (2021). Diel transcriptional oscillations of light-sensitive regulatory elements in open-ocean eukaryotic plankton communities. Proc. Natl. Acad. Sci. U. S. A., 118:e2011038118. 10.1073/pnas.2011038118 33547239 PMC8017926

[B21] Coesel S. N. Graff van Creveld S. Dugenne M. Henderikx-Freitas F. White A. E. Armbrust E. V. (2025). Proportional relationship between transcript concentrations and carbon biomass for open ocean plankton groups. ISME J. 19:wraf079. 10.1093/ismejo/wraf079 40302033 PMC12085914

[B22] Cohen N. R. McIlvin M. R. Moran D. M. Held N. A. Saunders J. K. Hawco N. J.. (2021). Dinoflagellates alter their carbon and nutrient metabolic strategies across environmental gradients in the central Pacific Ocean. Nat. Microbiol. 6, 173–186. 10.1038/s41564-020-00814-7 33398100

[B23] Connell P. E. Ribalet F. Armbrust E. V. White A. Caron D. A. (2020). Diel oscillations in the feeding activity of heterotrophic and mixotrophic nanoplankton in the North Pacific Subtropical Gyre. Aquat. Microb. Ecol. 85, 167–181. 10.3354/ame01950

[B24] Dave Karl Lab (2023). Gradients 3 KM1906 Organic and Inorganic Nutrients. Zenodo.

[B25] de Boyer Montégut C. Madec G. Fischer A. S. Lazar A. Iudicone D. (2004). Mixed layer depth over the global ocean: an examination of profile data and a profile-based climatology. J. Geophys. Res. Oceans 109:C12003. 10.1029/2004JC002378

[B26] Dong K. Wang Y. Zhang W. Li Q. (2024). Prevalence and preferred niche of small eukaryotes with mixotrophic potentials in the global ocean. Microorganisms 12:750. 10.3390/microorganisms12040750 38674694 PMC11051772

[B27] Edwards K. F. (2019). Mixotrophy in nanoflagellates across environmental gradients in the ocean. Proc. Natl. Acad. Sci. U. S. A. 116, 6211–6220. 10.1073/pnas.1814860116 30760589 PMC6442547

[B28] Edwards K. F. Li Q. McBeain K. A. Schvarcz C. R. Steward G. R. (2023). Trophic strategies explain the ocean niches of small eukaryotic phytoplankton. Proc. Roy. Soc. Lond. Ser. B Biol. Sci. 290:20222021. 10.1098/rspb.2022.2021 36695036 PMC9874276

[B29] Edwards K. F. Rii Y. M. Li Q. Peoples L. M. Church M. J. Steward G. F. (2024). Trophic strategies of picoeukaryotic phytoplankton vary over time and with depth in the North Pacific Subtropical Gyre. Environ. Microbiol. 26:e16689. 10.1111/1462-2920.16689 39168489

[B30] Finn R. D. Clements J. Eddy S. R. (2011). HMMER web server: interactive sequence similarity searching. Nucleic Acids Res. 39(suppl_2), W29–W37. 10.1093/nar/gkr367 21593126 PMC3125773

[B31] Flynn K. J. Mitra A. Anestis K. Anschütz A. A. Calbet A. Ferreira G. D.. (2019). Mixotrophic protists and a new paradigm for marine ecology: where does plankton research go now? J. Plankton Res. 41, 375–391. 10.1093/plankt/fbz026

[B32] Flynn K. J. Stoecker D. K. Mitra A. Raven J. A. Glibert P. M. Hansen P. J.. (2013). Misuse of the phytoplankton–zooplankton dichotomy: the need to assign organisms as mixotrophs within plankton functional types. J. Plankton Res. 35, 3–11. 10.1093/plankt/fbs062

[B33] Frias-Lopez J. Thompson A. Waldbauer J. Chisholm S. W. (2009). Use of stable isotope-labelled cells to identify active grazers of picocyanobacteria in ocean surface waters. Environ. Microbiol. 11, 512–525. 10.1111/j.1462-2920.2008.01793.x 19196281 PMC2702499

[B34] Gast R. J. Fay S. A. Sanders R. W. (2018). Mixotrophic activity and diversity of antarctic marine protists in Austral summer. Front. Mar. Sci. 5:13. 10.3389/fmars.2018.00013

[B35] Glibert P. M. Burkholder J. M. Kana T. M. Alexander J. Skelton H. Shilling C. (2009). Grazing by *Karenia brevis* on *Synechococcus* enhances its growth rate and may help to sustain blooms. Aquat. Microb. Ecol. 55, 17–30. 10.3354/ame01279

[B36] Grabherr M. G. Haas B. J. Yassour M. Levin J. Z. Thompson D. A. Amit I.. (2011). Full-length transcriptome assembly from RNA-seq data without a reference genome. Nat. Biotechnol. 29, 644–652. 10.1038/nbt.1883 21572440 PMC3571712

[B37] Graff van Creveld S. Coesel S. N. Blaskowski S. Groussman R. D. Schatz M. J. Armbrust E. V. (2023). Divergent functions of two clades of flavodoxin in diatoms mitigate oxidative stress and iron limitation. eLife 12:e84392. 10.7554/eLife.84392 37278403 PMC10287166

[B38] Groussman R. D. (2021). Diel-Regulated Transcriptional Cascades of Microbial Eukaryotes in the North Pacific Subtropical Gyre. Zenodo. 10.5281/zenodo.5009803 34659137 PMC8511712

[B39] Groussman R. D. Blaskowski S. Coesel S. N. Armbrust E. V. (2023a). MarFERReT, an open-source, version-controlled reference library of marine microbial eukaryote functional genes. Sci. Data 10:926. 10.1038/s41597-023-02842-4 38129449 PMC10739892

[B40] Groussman R. D. Blaskowski S. Coesel S. N. Armbrust E. V. (2023b). MarFERReT: An Open-Source, Version-Controlled Reference Library of Marine Microbial Eukaryote Functional Genes. Zenodo. 10.5281/zenodo.7055912 38129449 PMC10739892

[B41] Groussman R. D. Blaskowski S. Coesel S. N. Armbrust E. V. (2024b). The North Pacific Eukaryotic Gene Catalog: Metatranscriptome Assemblies With Taxonomy, Function and Abundance Annotations. Zenodo. 10.5281/zenodo.12630398 39438508 PMC11496615

[B42] Groussman R. D. Coesel S. N. Armbrust E. V. (2023c). The North Pacific Eukaryotic Gene Catalog: Raw Assemblies From Gradients 1, 2 and 3. Zenodo. 10.5281/zenodo.10699458 PMC11496615 39438508

[B43] Groussman R. D. Coesel S. N. Armbrust E. V. (2024a). The North Pacific Eukaryotic Gene Catalog: Clustered Nucleotide Metatranscripts and Read Counts. Zenodo. 10.5281/zenodo.13826820 PMC11496615 39438508

[B44] Groussman R. D. Coesel S. N. Durham B. P. Armbrust E. V. (2021). Diel-regulated transcriptional cascades of microbial eukaryotes in the North Pacific Subtropical Gyre. Front. Microbiol. 12:682651. 10.3389/fmicb.2021.682651 34659137 PMC8511712

[B45] Groussman R. D. Coesel S. N. Durham B. P. Schatz M. J. Armbrust E. V. (2024c). The North Pacific Eukaryotic Gene Catalog of metatranscriptome assemblies and annotations. Sci. Data 11:1161. 10.1038/s41597-024-04005-5 39438508 PMC11496615

[B46] Gumienny T. L. Brugnera E. Tosello-Trampont A. C. Kinchen J. M. Haney L. B. Nishiwaki K.. (2001). CED-12/ELMO, a novel member of the CrkII/Dock180/Rac pathway, is required for phagocytosis and cell migration. Cell 107, 27–41. 10.1016/S0092-8674(01)00520-7 11595183

[B47] Havskum H. Riemann B. (1996). Ecological importance of bacterivorous, pigmented flagellates (mixotrophs) in the Bay of Aarhus, Denmark. Mar. Ecol. Prog. Ser. 137, 251–263. 10.3354/meps137251

[B48] Hawco N. J. Conway T. M. Coesel S. N. Barone B. Seelen E. A. Yang S. C.. (2025). Anthropogenic iron alters the spring phytoplankton bloom in the North Pacific transition zone. Proc. Natl. Acad. Sci. U. S. A. 122:e2418201122. 10.1073/pnas.2418201122 40455985 PMC12168011

[B49] Hsu V. Pfab F. Moeller H. V. (2022). Niche expansion via acquired metabolism facilitates competitive dominance in planktonic communities. Ecology 103:e3693. 10.1002/ecy.3693 35349727

[B50] Hynes A. M. Winter J. Berthiaume C. T. Shimabukuro E. Cain K. White A.. (2024). High-frequency sampling captures variability in phytoplankton population-specific periodicity, growth, and productivity. Limnol. Oceanogr. 69, 2516–2531. 10.1002/lno.12683

[B51] Jacobson D. M. Andersen R. A. (1994). The discovery of mixotrophy in photosynthetic species of Dinophysis (Dinophyceae): light and electron microscopical observations of food vacuoles in *Dinophysis acuminata, D. norvegica* and two heterotrophic dinophysoid dinoflagellates. Phycologia 33, 97–110. 10.2216/i0031-8884-33-2-97.1

[B52] Jeong H. J. Yoo Y. D. Park J. Y. Song J. Y. Kim S. T. Lee S. H.. (2005). Feeding by phototrophic red-tide dinoflagellates: five species newly revealed and six species previously known to be mixotrophic. Aquat. Microb. Ecol. 40, 133–150. 10.3354/ame040133

[B53] Jimenez V. Burns J. A. Le Gall F. Not F. Vaulot D. (2021). No evidence of phago-mixotropy in *Micromonas polaris* (Mamiellophyceae), the dominant picophytoplankton species in the Arctic. J. Phycol. 57, 435–446. 10.1111/jpy.13125 33394518

[B54] John S. (2023). Gradients 1 and Gradients 2 Trace Metal Concentration Data. Zenodo. 10.5281/zenodo.7872301

[B55] Johnson L. K. Alexander H. Brown C. T. (2017). (All Datasets) MMETSP Re-assemblies. Zenodo. 10.5281/zenodo.3247846

[B56] Johnson L. K. Alexander H. Brown C. T. (2019). Re-assembly, quality evaluation, and annotation of 678 microbial eukaryotic reference transcriptomes. Gigascience 8:giy158. 10.1093/gigascience/giy158 30544207 PMC6481552

[B57] Jones H. L. J. Leadbeater B. S. C. Green J. C. (1993). Mixotrophy in marine species of *Chrysochromulina* (Prymnesiophyceae): ingestion and digestion of a small green flagellate. J. Mar. Biol. Assoc. U. K. 73, 283–296. 10.1017/S0025315400032859

[B58] Juranek L. W. (2020a). KM1906_Gradients3_Surface_O2Ar_NCP. Zenodo. 10.5281/zenodo.4009653

[B59] Juranek L. W. (2020b). KOK1606_Gradients1_Surface_O2Ar_NCP. Zenodo. 10.5281/zenodo.4104636

[B60] Juranek L. W. (2020c). MGL1704_Gradients2_Surface_O2Ar_NCP. Zenodo. 10.5281/zenodo.4079505

[B61] Juranek L. W. Quay P. D. Feely R. A. Lockwood D. Karl D. M. Church M. J. (2012). Biological production in the NE Pacific and its influence on air-sea CO_2_ flux: evidence from dissolved oxygen isotopes and O_2_/Ar. J. Geophys. Res. Oceans 117:C05022. 10.1029/2011JC007450

[B62] Juranek L. W. White A. E. Dugenne M. Henderikx Freitas F. Dutkiewicz S. Ribalet F.. (2020). The importance of the phytoplankton “middle class” to ocean net community production. Glob. Biogeochem. Cycles 34:e2020GB006702. 10.1029/2020GB006702

[B63] Kanehisa M. Goto S. (2000). KEGG: kyoto encyclopedia of genes and genomes. Nucleic Acids Res. 28, 27–30. 10.1093/nar/28.1.27 10592173 PMC102409

[B64] Kavanaugh M. T. Hales B. Saraceno M. Spitz Y. H. White A. E. Letelier R. M. (2014). Hierarchical and dynamic seascapes: a quantitative framework for scaling pelagic biogeochemistry and ecology. Prog. Oceanogr. 120, 291–304. 10.1016/j.pocean.2013.10.013

[B65] Keeling P. J. Burki F. Wilcox H. M. Bassem A. Allen E. E. Amaral-Zettler L. A.. (2014). The Marine Microbial Eukaryote Transcriptome Sequencing Project (MMETSP): illuminating the functional diversity of eukaryotic life in the oceans through transcriptome sequencing. PLoS Biol. 12:e1001889. 10.1371/journal.pbio.1001889 24959919 PMC4068987

[B66] Koppelle S. López-Escardó D. Brussaard C. P. Huisman J. Philippart C. J. M. Massana R.. (2022). Mixotrophy in the bloom-forming genus *Phaeocystis* and other haptophytes. Harmful Algae 117:102292. 10.1016/j.hal.2022.102292 35944956

[B67] Lambert B. S. Groussman R. D. Schatz M. J. Coesel S. N. Durham B. P. Alverson A. J.. (2022). The dynamic trophic architecture of open-ocean protist communities revealed through machine-guided metatranscriptomics. Proc. Natl. Acad. Sci. U. S. A. 119:e2100916119. 10.1073/pnas.2100916119 35145022 PMC8851463

[B68] Langmead B. Salzberg S. L. (2012). Fast gapped-read alignment with Bowtie 2. Nat. Methods 9, 357–359. 10.1038/nmeth.1923 22388286 PMC3322381

[B69] Lasek-Nesselquist E. Johnson M. D. (2019). A phylogenomic approach to clarifying the relationship of *Mesodinium* within the ciliophora: a case study in the complexity of mixed-species transcriptome analyses. Genome Biol. Evol. 11, 3218–3232. 10.1093/gbe/evz233 31665294 PMC6859813

[B70] Li A. Stoecker D. K. Adolf J. E. (1999). Feeding, pigmentation, photosynthesis and growth of the mixotrophic dinoflagellate *Gyrodinium galatheanum*. Aquat. Microb. Ecol. 19, 163–176. 10.3354/ame019163

[B71] Li A. Stoecker D. K. Coats D. W. (2000). Mixotrophy in *gyrodinium galatheanum* (DINOPHYCEAE): grazing responses to light intensity and inorganic nutrients^*^. J. Phycol. 36, 33–45. 10.1046/j.1529-8817.2000.98076.x

[B72] Li A. Stoecker D. K. Coats D. W. (2001). Use of the ‘food vacuole content' method to estimate grazing by the mixotrophic dinoflagellate *Gyrodinium Galatheanum* on cryptophytes. J. Plankton Res. 23, 303–318. 10.1093/plankt/23.3.303

[B73] Li A. Stoecker D. K. Coats D. W. Adam E. J. (1996). Ingestion of fluorescently labeled and phycoerythrin-containing prey by mixotrophic dinoflagellates. Aquat. Microb. Ecol. 10, 139–147. 10.3354/ame010139

[B74] Li Q. Edwards K. F. Schvarcz C. R. Steward G. F. (2022). Broad phylogenetic and functional diversity among mixotrophic consumers of *Prochlorococcus*. ISME J. 16, 1557–1569. 10.1038/s41396-022-01204-z 35145244 PMC9122939

[B75] Lie A. A. Y. Liu Z. Terrado R. Tatters A. O. Heidelberg K. B. Caron D. (2017). Effect of light and prey availability on gene expression of the mixotrophic chrysophyte, *Ochromonas* sp. BMC Genomics 18:163. 10.1186/s12864-017-3549-1 28196482 PMC5310065

[B76] Lie A. A. Y. Liu Z. Terrado R. Tatters A. O. Heidelberg K. B. Caron D. A. (2018). A tale of two mixotrophic chrysophytes: insights into the metabolisms of two *Ochromonas* species (Chrysophyceae) through a comparison of gene expression. PLoS ONE 13:e0192439. 10.1371/journal.pone.0192439 29438384 PMC5811012

[B77] Magoč T. Salzberg S. L. (2011). FLASH: fast length adjustment of short reads to improve genome assemblies. Bioinformatics 27, 2957–2963. 10.1093/bioinformatics/btr507 21903629 PMC3198573

[B78] Mak E. W. K. Turk-Kubo K. A. Caron D. A. Harbeitner R. C. Magasin J. D. Coale T. H.. (2024). Phagotrophy in the nitrogen-fixing haptophyte *Braarudosphaera bigelowii*. Environ. Microbiol. Rep. 16:e13312. 10.1111/1758-2229.13312 39049182 PMC11269211

[B79] McDougall T. J. Barker P. M. (2011). Getting started with TEOS-10 and the Gibbs Seawater (GSW) oceanographic toolbox. SCOR/IAPSO Working Group 127, 1–28.

[B80] McKie-Krisberg Z. Sanders R. W. Gast R. J. (2018). Evaluation of mixotrophy-associated gene expression in two species of polar marine algae. Front. Mar. Sci. 5:273. 10.3389/fmars.2018.00273

[B81] Mitra A. Caron D. A. Faure E. Flynn K. J. Leles S. G. Hansen P. J.. (2023a). The Mixoplankton Database (MDB): diversity of photo-phago-trophic plankton in form, function, and distribution across the global ocean. J. Eukaryot. Microbiol. 70:e12972. 10.1111/jeu.12972 36847544

[B82] Mitra A. Flynn K. J. Burkholder J. M. Berge T. Calbet A. Raven J. A.. (2014). The role of mixotrophic protists in the biological carbon pump. Biogeosciences 11, 995–1005. 10.5194/bg-11-995-2014

[B83] Mitra A. Flynn K. J. Stoecker D. K. Raven J. A. (2023b). Trait trade-offs in phagotrophic microalgae: the mixoplankton conundrum. Eur. J. Phycol. 59, 51–70. 10.1080/09670262.2023.2216259

[B84] Moeller H. V. Archibald K. M. Leles S. G. Pfab F. (2024). Predicting optimal mixotrophic metabolic strategies in the global ocean. Sci. Adv. 10:eadr0664. 10.1126/sciadv.adr0664 39671489 PMC11641107

[B85] NASA Goddard Space Flight Center Ocean Ecology Laboratory, Ocean Biology Processing Group. (2022). Moderate-resolution Imaging Spectroradiometer (MODIS) Aqua Photosynthetically Available Radiation Data; 2022 Reprocessing. NASA OB.DAAC, Greenbelt, MD, USA.

[B86] Nygaard K. Tobiesen A. (1993). Bacterivory in algae: a survival strategy during nutrient limitation. Limnol. Oceanogr. 38, 273–279. 10.4319/lo.1993.38.2.027330108563

[B87] Onyshchenko A. Roberts W. R. Ruck E. C. Lewis J. A. Alverson A. J. (2021). The genome of a nonphotosynthetic diatom provides insights into the metabolic shift to heterotrophy and constraints on the loss of photosynthesis. New Phytol. 232, 1750–1764. 10.1111/nph.17673 34379807 PMC9292941

[B88] Orsi W. D. Wilken S. del Campo J. Heger T. James E. Richards T. A.. (2018). Identifying protist consumers of photosynthetic picoeukaryotes in the surface ocean using stable isotope probing. Environ. Microbiol. 20, 815–827. 10.1111/1462-2920.14018 29215213

[B89] Park J. Durham B. P. Key R. S. Groussman R. D. Bartolek Z. Pinedo-Gonzalez P.. (2023). Siderophore production and utilization by marine bacteria in the North Pacific Ocean. Limnol. Oceanogr. 68, 1636–1653. 10.1002/lno.1237334341506

[B90] Patro R. Duggal G. Love M. I. Irizarry R. A. Kingsford C. (2017). Salmon provides fast and bias-aware quantification of transcript expression. Nat. Methods 14, 417–419. 10.1038/nmeth.4197 28263959 PMC5600148

[B91] Pinedo-González P. Hawco N. J. Bundy R. M. Armbrust E. V. Follows M. J. Cael B. B.. (2020). Anthropogenic Asian aerosols provide Fe to the North Pacific Ocean. Proc. Natl. Acad. Sci. U. S. A. 117, 27862–27868. 10.1073/pnas.2010315117 33093199 PMC7668086

[B92] Raven J. A. (1997). Phagotrophy in phototrophs. Limnol. Oceanogr. 42, 198–205. 10.4319/lo.1997.42.1.0198

[B93] Ribalet F. Berthiaume C. Hynes A. Swalwell J. Carlson M. Clayton S.. (2019). SeaFlow data v1, high-resolution abundance, size and biomass of small phytoplankton in the North Pacific. Sci. Data 6:277. 10.1038/s41597-019-0292-2 31757971 PMC6874581

[B94] Ribalet F. Berthiaume C. Hynes A. Swalwell J. Carlson M. Clayton S.. (2024). SeaFlow Data v1, High-Resolution Abundance, Size and Biomass of Small Phytoplankton in the North Pacific. Zenodo. 10.5281/zenodo.2678021 31757971 PMC6874581

[B95] Rice P. Longden I. Bleasby A. (2000). EMBOSS: the European molecular biology open software suite. Trends Genet. 16, 276–277. 10.1016/S0168-9525(00)02024-2 10827456

[B96] Rothhaupt K. O. (1996). Utilization of substitutable carbon and phosphorus sources by the mixotrophic chrysophyte *Ochromonas* Sp. Ecology 77, 706–715. 10.2307/2265495

[B97] Sibbald M. J. Albright L. J. (1991). The influence of light and nutrients on phagotrophy by the mixotrophic nanoflagellate *Ochromonas* sp. Mar. Microb. Food Webs 5, 39–47.

[B98] Simons CMAP Curator (2022). 2019 SCOPE Gradients Downcast CTD Data. Zenodo. 10.5281/zenodo.7015756

[B99] Stoecker D. K. Li A. Coats D. W. Gustafson D. E. Nannen M. K. (1997). Mixotrophy in the dinoflagellate *Prorocentrum minimum*. Mar. Ecol. Prog. Ser. 152, 1–12. 10.3354/meps152001

[B100] Suzuki S. Kawachi M. Tsukakoshi C. Nakamura A. Hagino K. Inouye I.. (2021). Unstable relationship between *Braarudosphaera bigelowii* (= *Chrysochromulina parkeae*) and its nitrogen-fixing endosymbiont. Front. Plant Sci. 12:749895. 10.3389/fpls.2021.749895 34925404 PMC8679911

[B101] Thomas E. Groussman M. J. Coesel S. N. Armbrust E. V. (2025a). Gradients 1-3 polyA-Selected Transcripts per Million, Gradients 3 Depth Profile polyA-Selected Processed Metatranscriptomes. Zenodo. 10.5281/zenodo.14519070

[B102] Thomas E. Groussman M. J. Coesel S. N. Armbrust E. V. (2025b). MarPRISM. Zenodo. 10.5281/zenodo.1451890227562213

[B103] Tillmann U. Salas R. Jauffrais T. Hess P. Silke J. (2014). “AZA: The producing organisms—Biology and trophic transfer”, in Seafood and Freshwater Toxins: Pharmacology, Physiology, and Detection, 3rd Edn, ed. L. M. Botana (Boca Raton, FL: CRC Press), 773–789. 10.1201/b16662-26

[B104] Van Vlierberghe M. Di Franco A. Philippe H. Baurain D. (2021). Decontamination, pooling and dereplication of the 678 samples of the Marine Microbial Eukaryote Transcriptome Sequencing Project. BMC Res. Notes 14:306. 10.1186/s13104-021-05717-2 34372933 PMC8353744

[B105] Viscardi M. J. Arribere J. A. (2022). Poly(a) selection introduces bias and undue noise in direct RNA-sequencing. BMC Genomics 23:530. 10.1186/s12864-022-08762-8 35869428 PMC9306060

[B106] Ward B. A. Follows M. J. (2016). Marine mixotrophy increases trophic transfer efficiency, mean organism size, and vertical carbon flux. Proc. Natl. Acad. Sci. U. S. A. 113, 2958–2963. 10.1073/pnas.1517118113 26831076 PMC4801304

[B107] White A. (2020). Gradients1-KOK1606-PPPCPN-UW_2020-03-10_v1.2. Zenodo. 10.5281/zenodo.3958307

[B108] White A. (2021). Gradients3-KM1906-PCPN-UW. Zenodo. 10.5281/zenodo.4840298

[B109] Wilken S. Choi C. J. Worden A. Z. (2019). Contrasting mixotrophic lifestyles reveal different ecological niches in two closely related marine protists. J. Phycol. 56, 52–67. 10.1111/jpy.12920 31529498 PMC7065223

[B110] Wilson S. T. Aylward F. O. Ribalet F. Barone B. Casey J. R. Connell P. E.. (2017). Coordinated regulation of growth, activity and transcription in natural populations of the unicellular nitrogen-fixing cyanobacterium *Crocosphaera*. Nat. Microbiol. 2:17118. 10.1038/nmicrobiol.2017.118 28758990

[B111] Zubkov M. V. Tarran G. A. (2008). High bacterivory by the smallest phytoplankton in the North Atlantic Ocean. Nature 455, 224–226. 10.1038/nature07236 18690208

