# Supplementary material for: Latitude- and depth-driven divergence in protist trophic strategies revealed by a machine learning model
Source: Front Microbiol. 2025 Sep 1;16:1602162. doi: 10.3389/fmicb.2025.1602162 (PMC12434009; doi:10.3389/fmicb.2025.1602162)
Supplement: Supplementary file 1 [file Supplementary_file_1.docx]

Supplementary Material

**
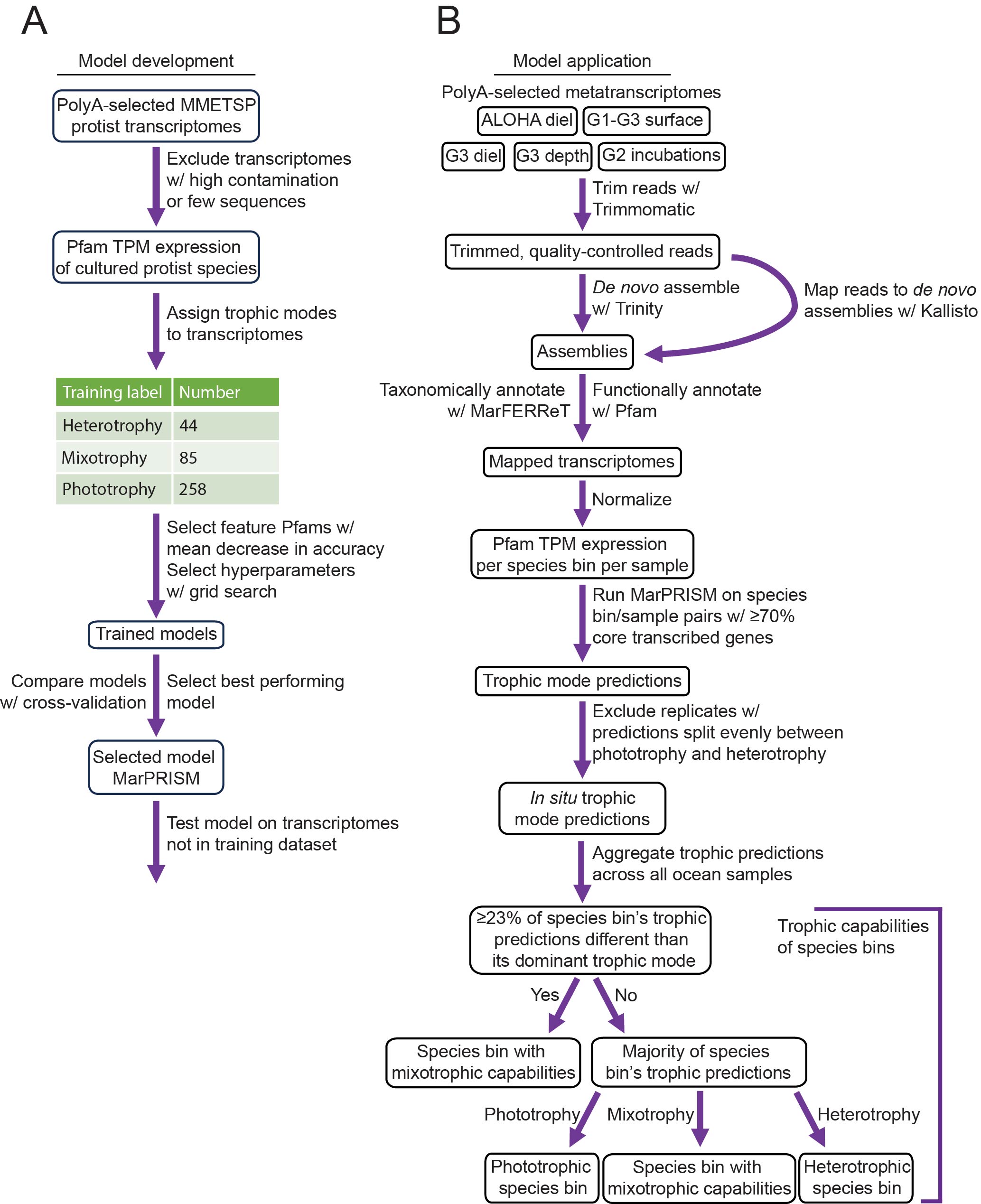
Supplementary Figure 1.** Flowchart of model development and application. (A) Machine-learning model development for predicting the *in situ* trophic mode of marine protist species bins. (B) Model application to North Pacific Ocean metatranscriptomes. MMETSP stands for Marine Microbial Eukaryote Transcriptome Sequencing Project, TPM transcripts per million, MarPRISM Marine PRotist *In Situ* trophic Mode predictor, G1-G3 Gradients cruises, and MarFERReT Marine Functional EukaRyotic Reference Taxa.**
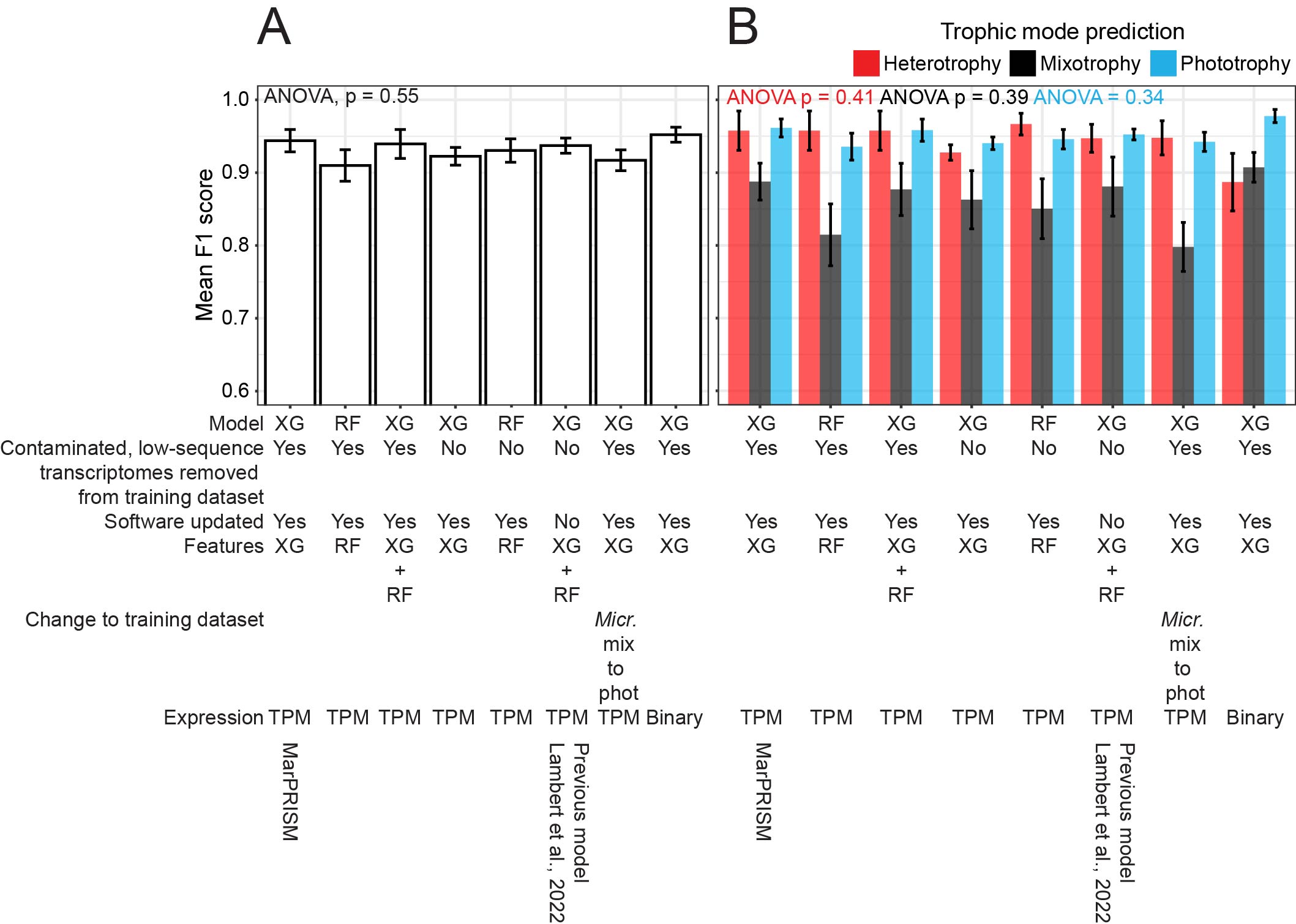
Supplementary Figure 2.** Performance of XGBoost (XG) and Random Forest (RF) machine learning models using different training datasets, software versions, and types of feature expression values. (A) Mean F1 (two ⋅ precision ⋅ recall/(precision + recall)) score of machine learning models for predictions overall determined from 6-fold cross-validation. The p-value is provided for the comparison of F1 scores between models, as determined by one-way ANOVA. (B) Mean F1 score of machine learning models for predictions separated by trophic mode determined from 6-fold cross-validation. P-values are provided for comparisons of F1 scores between models for each trophic mode (color), as determined by one-way ANOVAs. Random Forest and XGBoost models were tested with different training datasets (contaminated and low-sequence transcriptomes removed vs. contaminated and low-sequence entries not removed), with different software versions of XGBoost (0.90 vs. 1.7.4) and Random Forest (0.21.3 vs. 1.5.1) models, with different Pfam feature sets (features determined for Random Forest, XGBoost, vs. the union of Random Forest and XGBoost features), with permutations to the training dataset (transcriptomes from *Micromonas* species with mixotrophic labels converted to phototrophic labels), and with different feature expression values (transcripts per million (TPM) vs. binary (TPM values > 0 were converted to 1)). Error bars represent the standard error of 6-fold cross-validation.
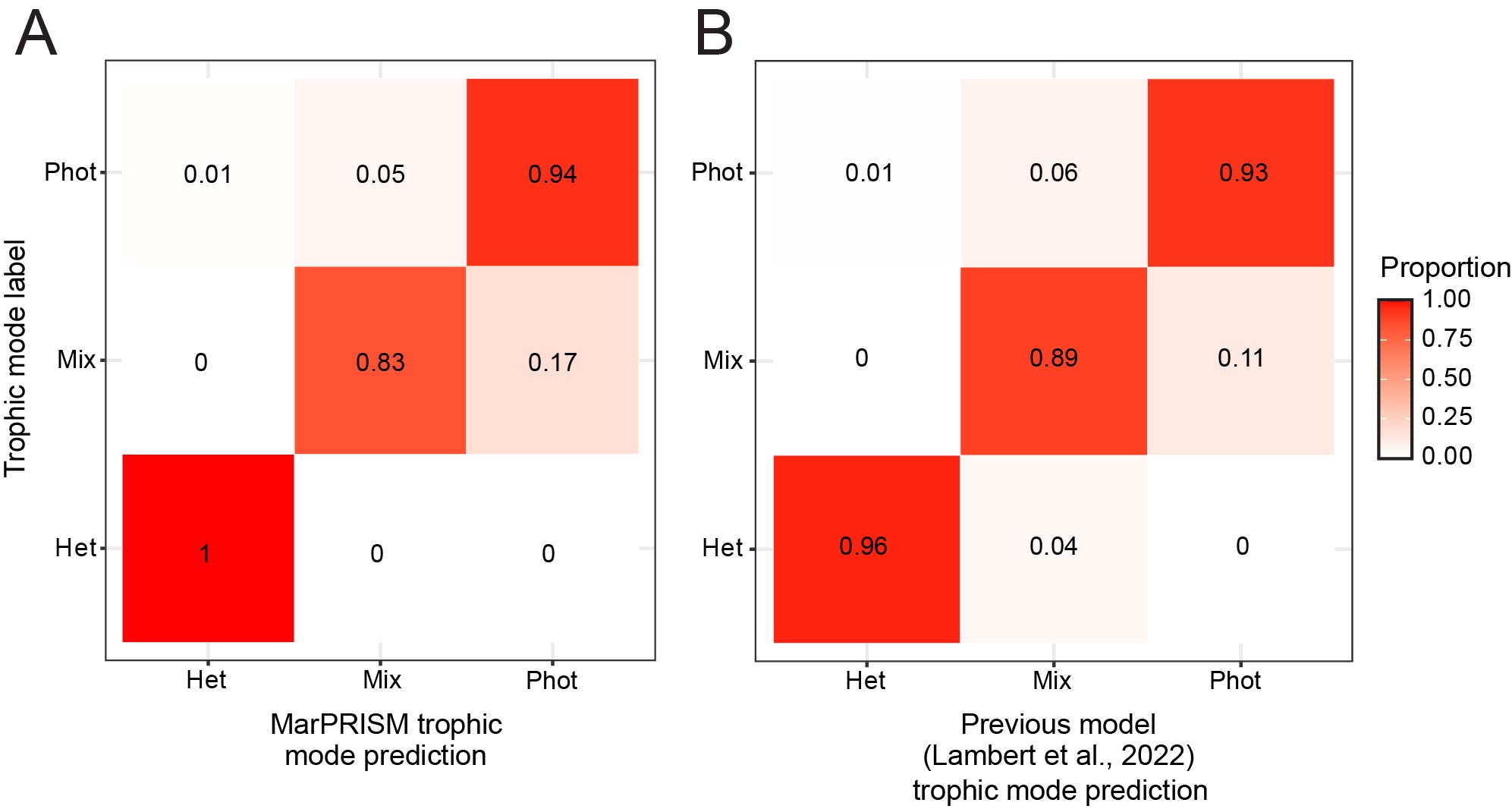
**Supplementary Figure 3.** Cumulative confusion matrices from 6-fold cross-validation of MarPRISM and the previous version of the model (Lambert et al., 2022). Panels show the proportion of predictions in each confusion matrix cell for (A) MarPRISM and (B) the earlier model.

**
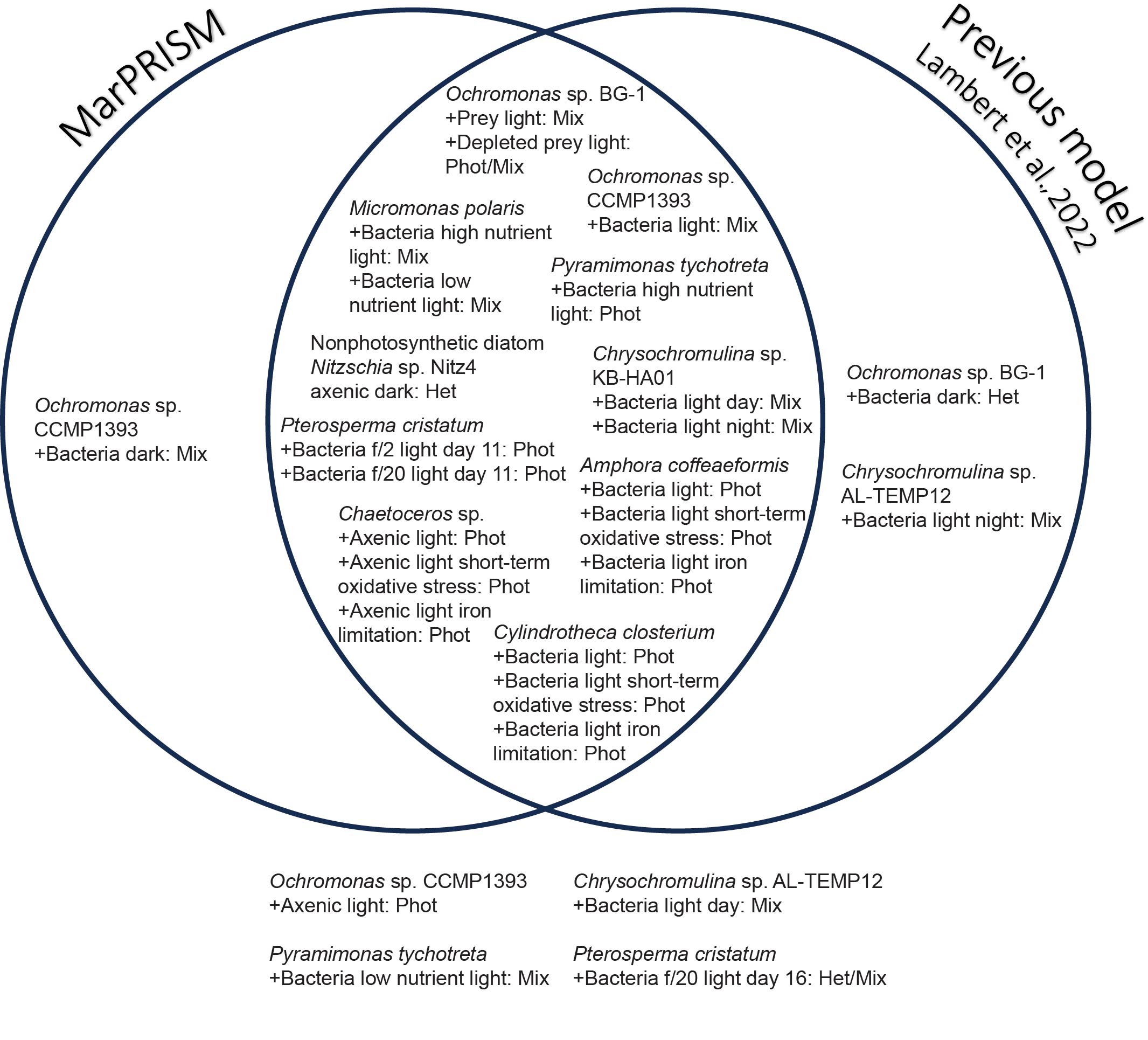
Supplementary Figure 4.** Results of MarPRISM and the previous version of the model (Lambert et al., 2022) tested on cultured protist species transcriptomes not present in the training dataset for either model. Transcriptomes are included in the Venn diagram based on which model(s) correctly predicted their expected trophic mode across all replicates. Neither model predicted the expected trophic mode correctly across all replicates for the transcriptomes outside of the Venn diagram. For the transcriptomes that at least one model correctly predicted the expected trophic mode, the predicted trophic mode is provided. For the transcriptomes that neither model successfully predicted the expected trophic mode, the expected trophic mode is provided.


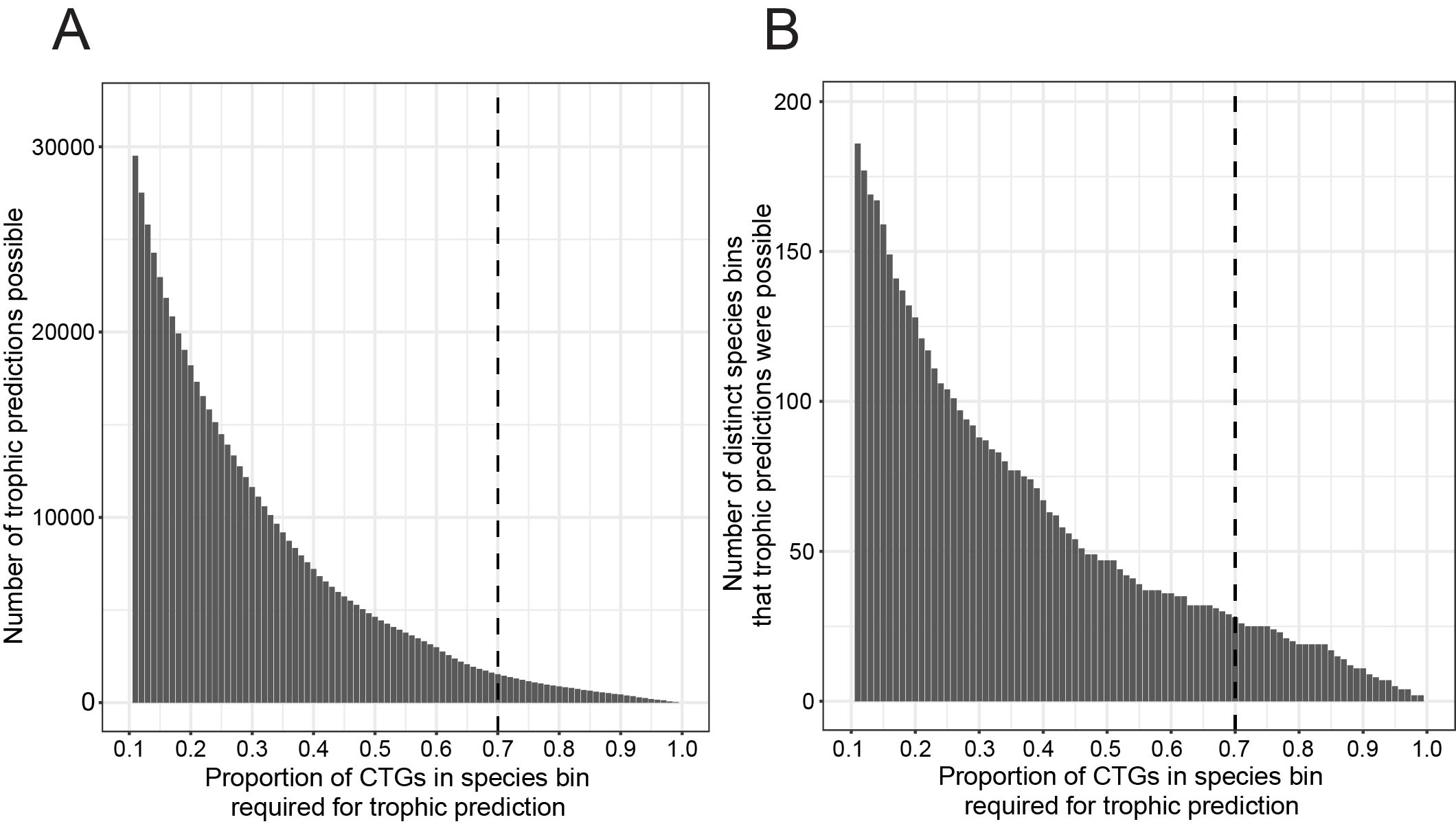
**Supplementary Figure 5.** Sensitivity of results to proportion of Core Transcribed Genes (CTG) required to be expressed for a species bin in a sample to receive a trophic prediction. For different proportions of CTGs required, (A) the total number of trophic predictions possible for protist species bins and (B) the total number of distinct protist species bins for which trophic predictions were possible across five cruise datasets: G1-G3 surface, ALOHA diel, G2 incubation, G3 diel, and G3 depth profile samples. 70% CTG recovery is marked with a dashed line.
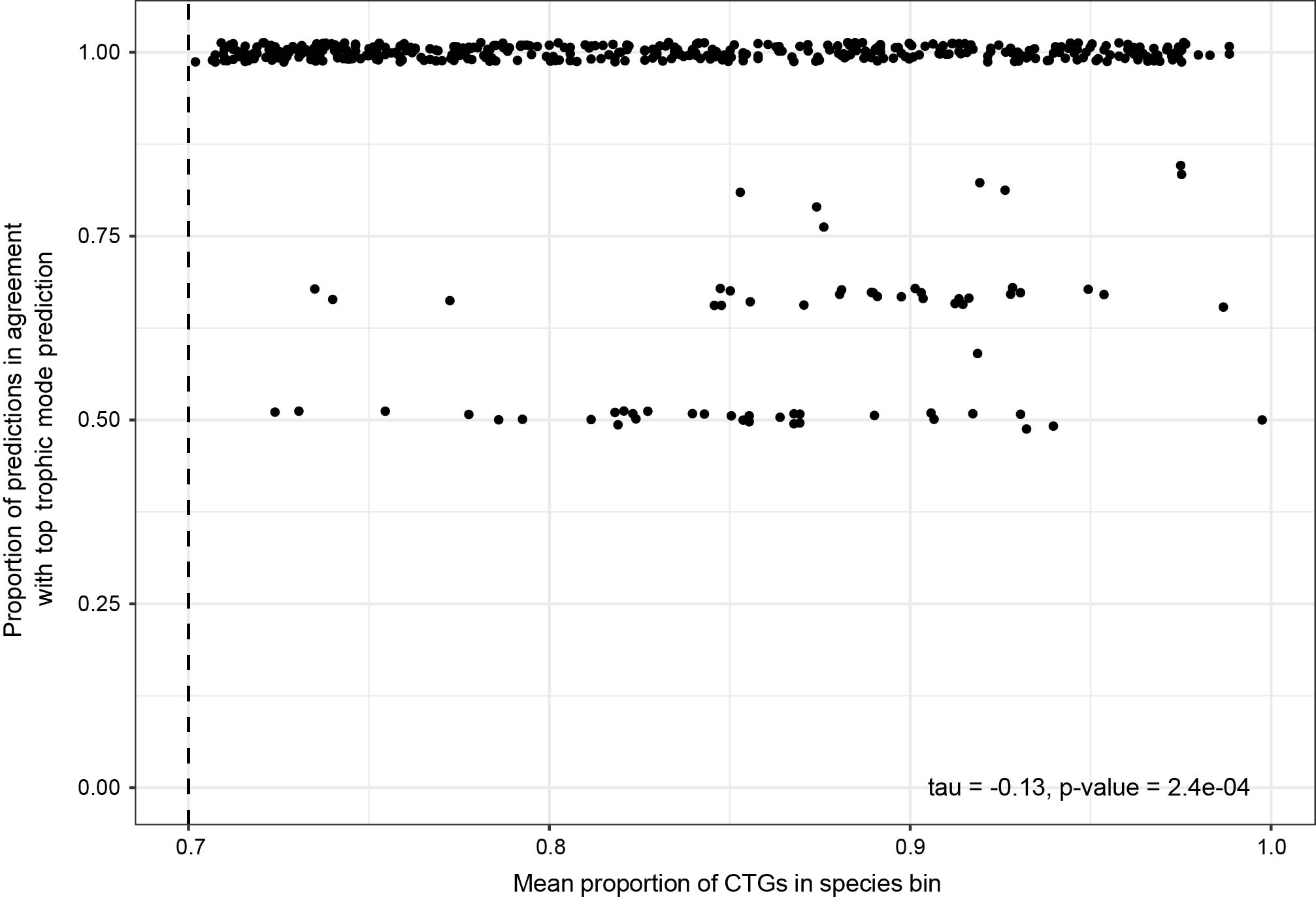
**Supplementary Figure 6.** Proportion of trophic predictions in agreement with the most abundant trophic prediction among replicates and size fractions collected from the same environment compared to Core Transcribed Gene (CTG) recovery across five cruise datasets: G1-G3 surface, ALOHA diel, G2 incubation, G3 diel, and G3 depth profile samples. First, predictions determined to be from model failure were excluded. For a proportion of predictions in agreement to be calculated, more than one metatranscriptome from the particular environment had to have a trophic prediction for the species bin. Each point represents multiple replicates and/or size fractions for a species bin. The proportion of CTGs expressed in the species bin was averaged across replicates and size fractions. 70% CTG recovery is marked with a dashed line. Tau and p-value of Kendall rank correlation test are provided.


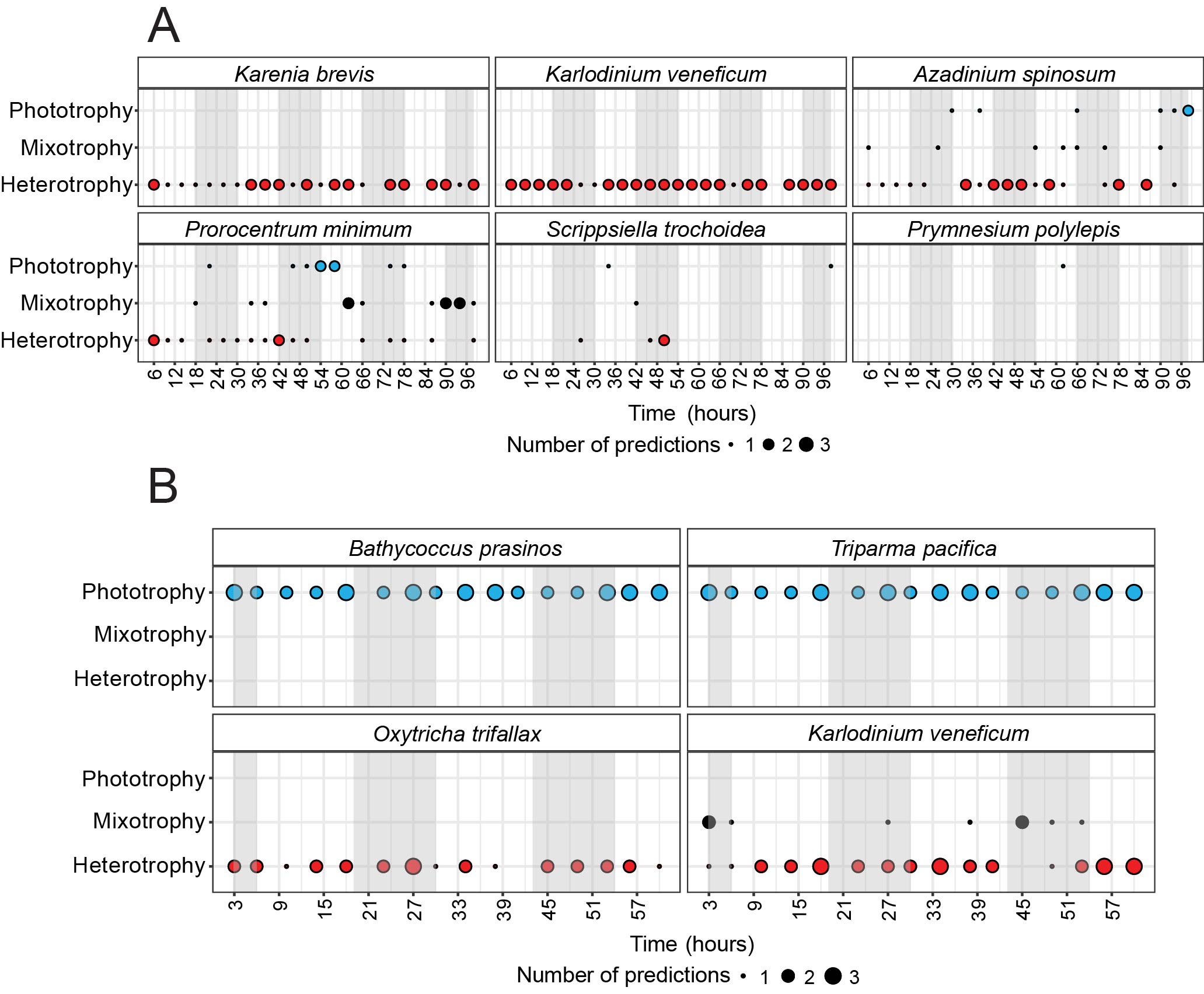
**Supplementary Figure 7.** Trophic predictions of species bins in the surface ocean across diel studies. Number of predictions of each trophic mode for species bins during the (A) Station ALOHA (158°W, 22.75°N) and (B) G3 diel studies (158°W, 41.6°N) summed across replicates. The x-axis represents the number of hours since the start of sampling during each diel study. Grey shading represents darkness during the diel cycle.

**
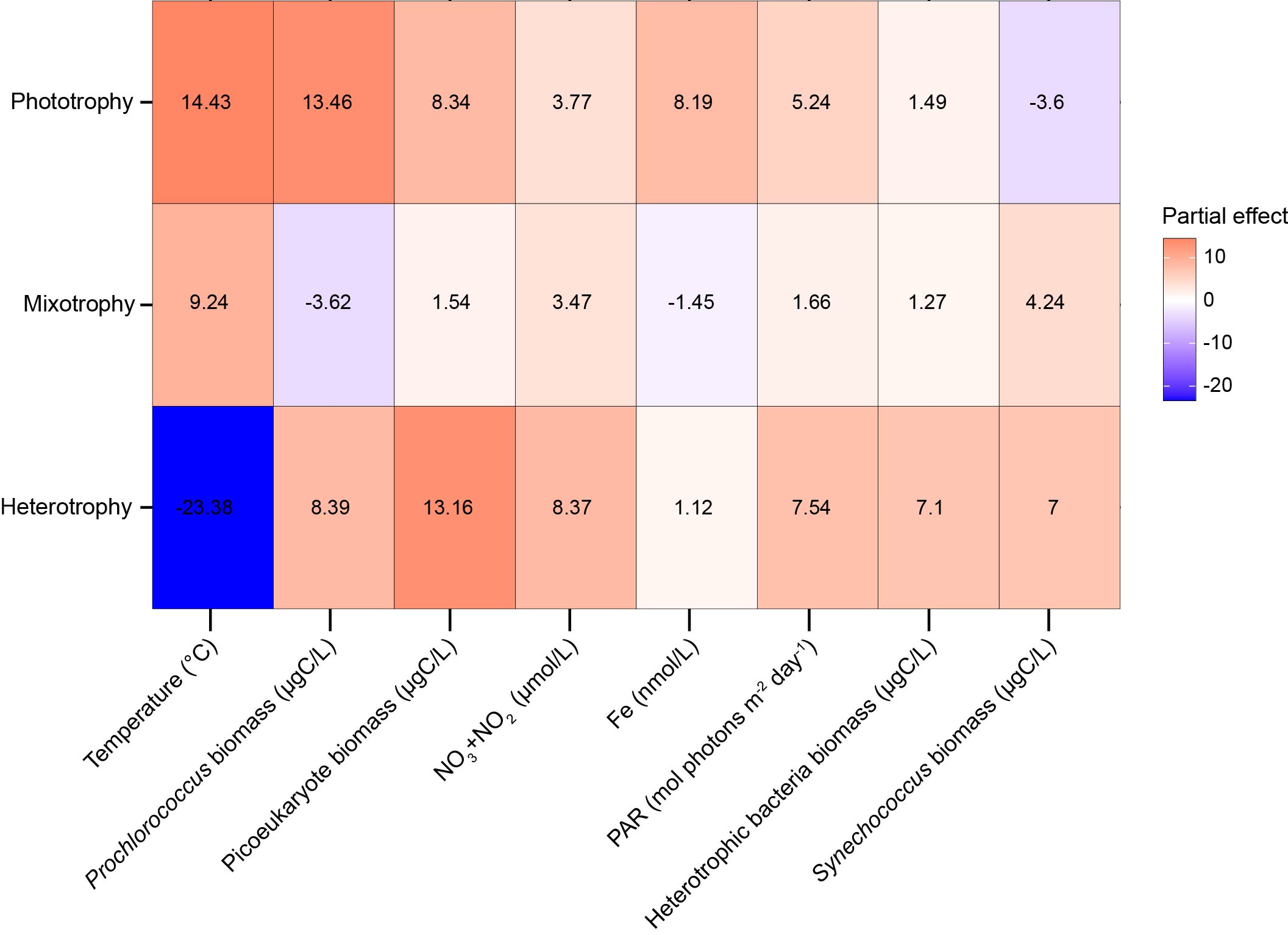
Supplementary Figure 8.** Partial effects of the number of *in situ* trophic mode predictions for species bins with mixotrophic capabilities across the G1-G3 surface transects in relation to surface measurements of nitrate/nitrite, iron, surface photosynthetically active radiation (PAR), temperature, and the biomass of *Prochlorococcus*, *Synechococcus*, heterotrophic bacteria, and picoeukaryotes. Trophic predictions and environmental data were aligned by cruise and proximity in latitude (within 0.5°), selecting the closest metadata measurement or averaging in the case of ties. Generalized Additive Models (GAMs) were applied to quantify the partial effects of these environmental variables on the number of predictions for each trophic mode. No significant partial effects were detected (GAMs assessing partial effects, multiple hypothesis testing using Benjamini-Hochberg method, adjusted p-values > 0.05). The environmental variables are ordered from highest to lowest mean absolute partial effect.


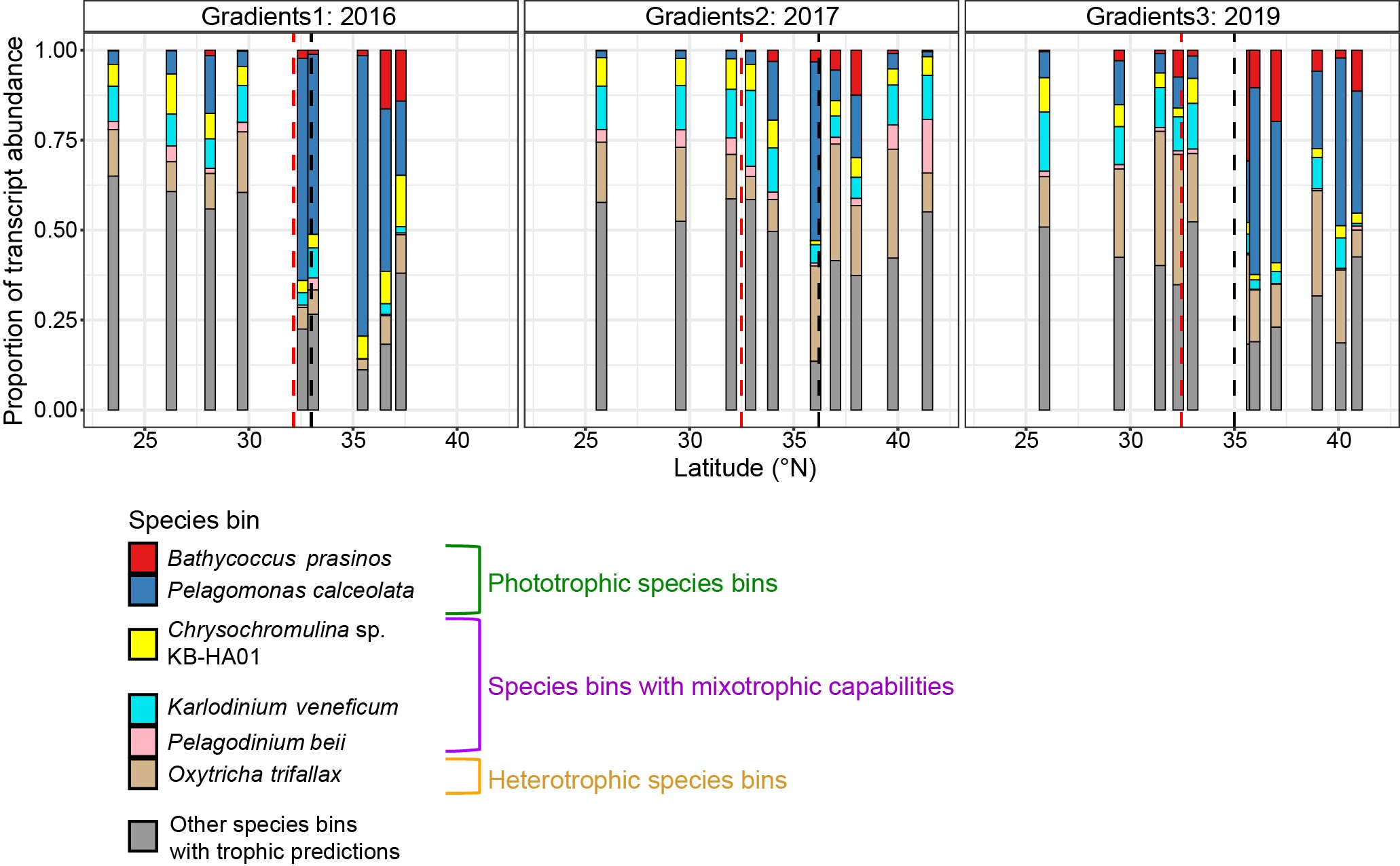
**Supplementary Figure 9.** Transcript abundance across the G1-G3 surface transects for the 28 species bins for which trophic predictions were possible. Proportion of transcript abundance by species bin at the surface across the three cruises. Only the species bins with the highest average transcript abundance for at least one latitude are given a unique color. Transcripts per liter for dinoflagellate species bins were corrected by dividing by 6.4 (Coesel et al., 2025). The transcript abundance for each species bin was averaged across replicates, then divided by the total transcript abundance of the 28 species bins at each latitude. Red dashed line: location of salinity isohaline (34.82); black dashed line: location of transition zone chlorophyll front (0.15 mg m^−3^ chlorophyll).
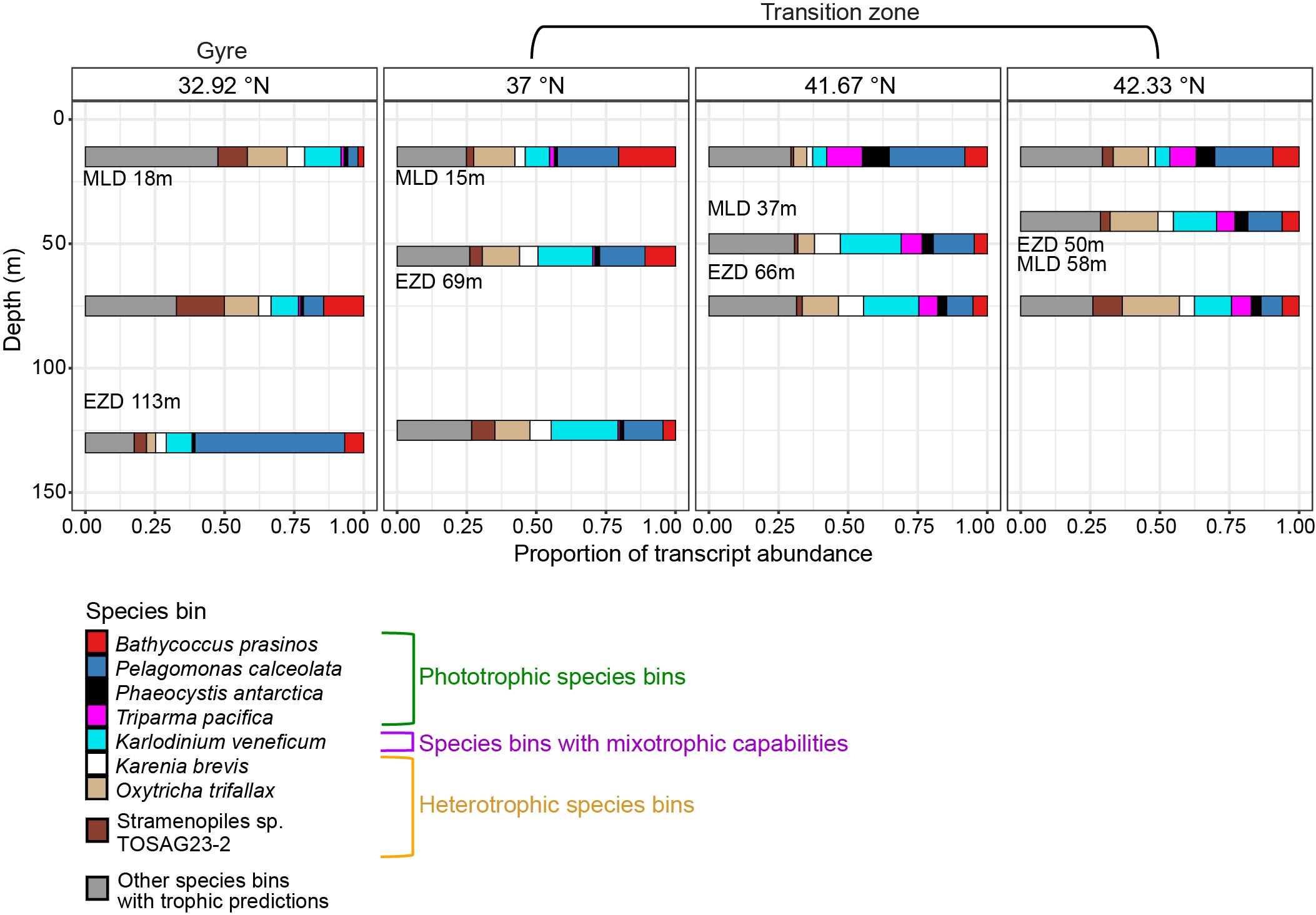
**Supplementary Figure 10.** Transcript abundance throughout the G3 depth profiles for the 28 species bins for which trophic predictions were possible. Proportion of transcript abundance by species bin with depth. Only the species bins with the first to third highest transcript abundance for at least one latitude, depth pair are given a unique color. Transcripts per liter for dinoflagellate species bins were corrected by dividing by 6.4 (Coesel et al., 2025). The transcript abundance for each species bin was averaged across replicates, then divided by the total transcript abundance of the 28 species bins at each latitude and depth. Two replicates were collected at three depths for each depth profile except 130 m at 32.92°N which had just one replicate. The approximate mixed layer depth (MLD) and euphotic zone depth (EZD, 1% surface PAR) are labeled for each depth profile.

**Supplementary Data Sheet 1.** Training dataset with contaminated and low-sequence entries removed. This is the training dataset used for MarPRISM. PolyA-selected marine eukaryotic transcriptomes were generated through the Marine Microbial Eukaryote Transcriptome Sequencing Project (MMETSP). The assemblies, functional annotations, and mapping results for these transcriptomes were derived from re-assembly efforts (Johnson et al. 2019; Patro et al. 2017). Contaminated and low-sequence entries removed: entries had to have at least 1200 total sequences, at least 500 total assigned Pfam domains, and less than 50% contamination from non-target organisms (percent of ribosomal protein sequences with taxonomic identity other than the recorded identity). Contamination rates for the MMETSP transcriptomes were taken from (Lasek-Nesselquist and Johnson, 2019; Van Vlierberghe et al., 2021; Groussman et al., 2023a). 387 of the MMETSP transcriptomes that passed these cutoffs could be assigned a trophic mode label. The trophic mode labels were assigned in (Lambert et al., 2022). Ax: culture was grown axenically. Dark: culture was grown in the dark. Reference shows where transcriptome was published.

**Supplementary Data Sheet 2.** The contaminated and low-sequence Marine Microbial Eukaryote Transcriptome Sequencing Project (MMETSP)-generated entries. Contaminated and low-sequence entries: entries had to have at least 1200 total sequences, at least 500 total assigned Pfam domains, and less than 50% contamination from non-target organisms (percent of ribosomal protein sequences with taxonomic identity other than the recorded identity). Contamination rates for the MMETSP-generated transcriptomes were taken from (Lasek-Nesselquist and Johnson, 2019; Van Vlierberghe et al., 2021; Groussman et al., 2023a). The union of this table and Table S1 is the set of MMETSP entries used by the previous version of the model (Lambert et al., 2022). No light: culture was not grown in the light.

**Supplementary Data Sheet 3.** Transcriptomes used for testing MarPRISM and the previous version of the model (Lambert et al., 2022), and the resulting trophic predictions. Transcriptomes were collected from publicly available sources and were not included in the training dataset for either model. The expected trophic prediction is based on the known trophic capabilities and culture conditions of the species as well as the detection of prey ingestion and transcriptomic analysis if described in the reference.

**Supplementary Data Sheet 4.** Transcriptomes removed from the training dataset for MarPRISM due to high contamination or low-sequence abundance that were used to test MarPRISM and the resulting trophic predictions. Transcriptomes were removed from the training dataset and used for testing that had less than 1200 total sequences, less than 500 total assigned Pfam domains, and/or greater than 50% contamination from non-target organisms (percent of ribosomal protein sequences with taxonomic identity other than the recorded identity). Contamination and low-sequence entries were identified by (Lasek-Nesselquist and Johnson, 2019; Van Vlierberghe et al., 2021; Groussman et al., 2023a) and curated by (Groussman et al., 2023a). Identity of ribosomal sequences was analyzed by (Groussman et al., 2023a).

qc_flag_Groussman: LOW_SEQS; less than 1,200 raw sequences; LOW_PFAMS; less than 500 Pfam annotations.

num_sequences_Groussman: Number of sequences in original sequence file.

num_pfams_Groussman: Number of Pfams identified in protein sequences.

flag_Lasek: Flag notes from Lasek-Nesselquist and Johnson (2019); CONTAM NOTED; ciliate samples reported as contaminated in this study.

flag_VanVlierberghe: Flag for a high level of estimated contamination from Van Vlierberghe et al. (2021); CONTAM_50PCT; contamination percentages over 50%.

flag_ribosomalContamination_Groussman: Flag for a high level of estimated contamination, from ‘ribosomal_contam_pct_Groussman'; CONTAM_50PCT; contamination percentages over 50%.

ribosomal_contam_pct_Groussman: Percent of total ribosomal protein sequences with an inferred taxonomic identity in any lineage other than the recorded identity.

Ribosomal taxonomy of most abundant contaminant: For entries with greater than 50% ribosomal protein sequences with an inferred taxonomic identity in any lineage other than the recorded identity, the taxonomic identity of the most abundant ribosomal protein sequences not identified as the recorded identity of the transcriptome in the MMETSP.

Expected taxonomy of transcriptome: Recorded identity in MMETSP.

**Supplementary Data Sheet 5.** Details on collection of the metatranscriptomes from North Pacific Ocean: cruise name, cruise number, research vessel, dates of metatranscriptomic sampling (HST: Hawaii Standard Time), locations of metatranscriptomic sampling, method of sampling, depths of metatranscriptomic sampling, method of seawater collection for metatranscriptomes, times of seawater collection (HST) for metatranscriptomes, number of metatranscriptome replicates, and size fraction of metatranscriptomes. Seawater was sequentially filtered through size fractions when multiple size fractions were collected.

**Supplementary Data Sheet 6.** Details on nutrient amendment incubations conducted during G2, including amounts of iron, nitrate, and phosphate added for different treatments.

**Supplementary Data Sheet 7.** Sources of G1-G3 nutrient, irradiance, net community production, temperature, bacteria and picoeukaryote biomass data.

**Supplementary Data Sheet 8.** Best performing hyperparameters for Random Forest and XGBoost models based on different training datasets and types of feature expression values. For the XGBoost model and dataset with contaminated and low-sequence entries removed, the best performing hyperparameters were also determined after permutating some trophic mode labels in the training dataset (transcriptomes from *Micromonas* species with mixotrophic labels were converted to phototrophic labels). For the XGBoost model and dataset with contaminated and low-sequence entries removed, the best performing hyparameters were also determined for when binary was used as the metric for feature expression (TPM values > 0 were converted to 1). Grid searches with Scikit-learn’s GridSearchCV and five-fold cross-validation were run on a randomly sampled set of 100 phototrophic transcriptomes (randomly sampled one time for each training dataset), as well as all of the mixotrophic and heterotrophic transcriptomes from the different training datasets. The following hyperparameters, n_estimators (10, 100, 1000), max_depth (3, 10, 20), learning_rate (0.05, 0.1, 0.15, 0.2), gamma (0, 0.5, 1), and reg_lambda (0, 0.5, 1) were tested for XGBoost. The hyperparameters, n_estimators (10, 100, 1000, 10000), max_depth (1, 10, 1000, None), min_samples_split (2, 5, 10, 20), min_samples_leaf (1, 3, 5, 10), and min_weight_fraction_leaf (0, 0.2, 0.5) were tested for Random Forest. The F1 score was calculated for each combination of hyperparameters. The hyperparameters with the highest mean F1 score (averaged across five-fold cross-validation) were selected for each model and training dataset.

**Supplementary Data Sheet 9.** The 183 MarPRISM feature Pfams. Their function within the Pfam database is included. Six broad categories (carbon metabolism, motility, phagocytosis, photosynthesis, signaling, and transcription and translation) could be manually assigned to 106/183 of the feature Pfams based on their function. The importance score of the feature Pfams was determined from mean decrease in accuracy run for an XGBoost model on four versions of the training dataset with contaminated and low-sequence entries removed, each with a single random subset of phototrophic transcriptomes of a different subsample size (number of phototrophic transcriptomes = 50, 80, 100, 120), along with all of the mixotrophic and heterotrophic transcriptomes. Included is each feature Pfam’s median expression in transcripts per million (TPM) in the phototrophic, mixotrophic, and heterotrophic transcriptomes in the training dataset after removing the contaminated and low-sequence entries.

**Supplementary Data Sheet 10.** Trophic predictions excluded for being split between phototrophy and heterotrophy, as these predictions were hypothesized to be in conflict with model decision boundaries. For non-diel samples, we excluded instances where both phototrophy and heterotrophy received >25% of trophic predictions across replicates or size fractions at a given location for one species bin. For diel samples, we excluded instances where both phototrophy and heterotrophy received >25% of trophic predictions across samples and timepoints in a given day for one species bin.

**Supplementary Data Sheet 11.** Number and proportion of trophic predictions excluded for being split between phototrophy and heterotrophy per species bin, as these predictions were hypothesized to be in conflict with model decision boundaries. For non-diel samples, we excluded instances where both phototrophy and heterotrophy received >25% of trophic predictions across replicates or size fractions at a given location for one species bin. For diel samples, we excluded instances where both phototrophy and heterotrophy received >25% of trophic predictions across samples and timepoints in a given day for one species bin.

**Supplementary Data Sheet 12.** Every trophic prediction across surface, depth, incubation, and diel samples after removing trophic predictions split between phototrophy and heterotrophy. For non-diel samples, we excluded instances where both phototrophy and heterotrophy received >25% of trophic predictions across replicates or size fractions at a given location for one species bin. For diel samples, we excluded instances where both phototrophy and heterotrophy received >25% of trophic predictions across samples and timepoints in a given day for one species bin. HST: Hawaii Standard Time.

**Supplementary Data Sheet 13.** Number of trophic predictions by cruise dataset and species bin after removing trophic predictions split between phototrophy and heterotrophy. For non-diel samples, we excluded instances where both phototrophy and heterotrophy received >25% of trophic predictions across replicates or size fractions at a given location for one species bin. For diel samples, we excluded instances where both phototrophy and heterotrophy received >25% of trophic predictions across samples and timepoints in a given day for one species bin.

**Supplementary Data Sheet 14.** Transcript abundance of the 28 species bins with trophic predictions. Transcripts per liter were calculated using counts of the custom standards. Transcript abundance is provided for every sample that the species bin appeared in not just for samples that had a trophic prediction for that species bin. Transcripts per liter for dinoflagellate species bins were corrected by dividing by 6.4 (Coesel et al., 2025).

**Supplementary Data Sheet 15.** Species bins are labeled with the species name of their closest relative based on last common ancestor analysis, and whether previous literature (Nygaard and Tobiesen, 1993; Chang and Carpenter, 1994; Jacobson and Andersen, 1994; Havskum and Riemann, 1996; Li et al., 1996; Stoecker et al., 1997; Li et al., 1999; Li et al., 2001; Jeong et al., 2005; Calbet et al., 2011; Tillmann et al., 2014; Gast et al., 2018; Glibert et al., 2009; Avrahami and Frada, 2020; Koppelle et al., 2022; Lambert et al., 2022; Li et al., 2022), many collected by Mitra et al. (2023a), indicated their closest relative to be a phototroph, heterotroph, constitutive mixotroph, or plastidic specialist non-constitutive mixotroph, followed by ? if the trophic capabilities were uncertain due to disagreement or low taxonomic resolution in the literature, or whether the trophic capabilities of their closest relative were Unknown. Trophic capabilities of the species bins based on aggregated trophic predictions were defined as follows. Mixotrophic capabilities: ≥23% of its predictions were assigned trophic mode(s) different from its majority trophic mode, and the different trophic predictions were not solely split between replicates or size fractions. Heterotrophic: received all or almost all heterotrophy predictions (<23% non-heterotrophy predictions). Phototrophic: received all or almost all phototrophy predictions (<23% non-phototrophy predictions). Number of predictions for each trophic mode aggregated across G1-G3 surface, ALOHA diel, G2 incubation, G3 diel, and G3 depth profile samples by species bin after removing trophic predictions split between phototrophy and heterotrophy. For non-diel samples, we excluded instances where both phototrophy and heterotrophy received >25% of trophic predictions across replicates or size fractions at a given location for one species bin. For diel samples, we excluded instances where both phototrophy and heterotrophy received >25% of trophic predictions across samples and timepoints in a given day for one species bin.

**Data availability**

Code for running MarPRISM; feature and hyperparameter search and cross-validation for MarPRISM and other models tested: <https://github.com/armbrustlab/MarPRISM>.

Data used for running, feature and hyperparameter search, and cross-validation for MarPRISM and other models tested: <https://doi.org/10.5281/zenodo.14518902> (Thomas et al., 2025b).

The training dataset originates from MMETSP-derived transcriptomes assembled, functionally annotated, and mapped by (Johnson et al., 2019).

- Their MMETSP re-assemblies and functional annotations: https://doi.org/10.5281/zenodo.3247846 (Johnson et al., 2017)
- Their code for assembly, mapping, and functional annotation of the MMETSP-derived transcriptomes: <https://github.com/dib-lab/dib-MMETSP>
- Entries to remove from the training dataset based on low sequence abundance or high contamination were pulled from: <https://zenodo.org/records/10553848> (Groussman et al., 2023b)

Accession IDs for transcriptomes used to test MarPRISM are available in Supplementary Data Sheet 3.

- TPM of these transcriptomes is available here: <https://doi.org/10.5281/zenodo.14518902> (Thomas et al., 2025b).

Raw short reads and assemblies from the North Pacific Ocean metatranscriptomes in this study are available through NCBI’s Short Read Archive (SRA) and the Transcriptome Shotgun Assembly (TSA) sequence database.

- PRJNA492142: ALOHA diel
- PRJNA690573: G1 surface
- PRJNA1076191: G2 surface
- PRJNA690575: G2 incubations
- PRJNA1077380: G3 surface
- PRJNA1076851: G3 diel
- PRJNA1148215: G3 depth

The ALOHA diel, G1-G3 surface, and G3 diel samples are part of the North Pacific Eukaryotic Gene Catalog (Groussman et al., 2024c). The taxonomic and functional annotations, and estimated count files for these samples are described in [(](https://paperpile.com/c/mgrVnz/rSRD)Groussman et al., 2024c[)](https://paperpile.com/c/mgrVnz/rSRD) and can be found across the following Zenodo repositories.

- [https://doi.org/](https://doi.org/10.5281/zenodo.14519070)[10.5281/zenodo.5009803](https://doi.org/10.5281/zenodo.5009803) (Groussman, 2021): raw metatranscriptomic assemblies for ALOHA diel samples; these samples were described in a previous publication (Groussman et al. 2021)
- [https://doi.org/](https://doi.org/10.5281/zenodo.14519070)[10.5281/zenodo.10699458](https://doi.org/10.5281/zenodo.10699458) (Groussman et al., 2023c): raw metatranscriptomic assemblies for G1-G3 surface and G3 diel samples
- [https://doi.org/](https://doi.org/10.5281/zenodo.14519070)[10.5281/zenodo.](https://doi.org/10.5281/zenodo.10699458)[12630398](https://zenodo.org/records/12630398) (Groussman et al., 2024b): processed and translated protein sequences for ALOHA diel, G1-G3 surface, and G3 diel samples
- [https://doi.org/](https://doi.org/10.5281/zenodo.14519070)[10.5281/zenodo.](https://doi.org/10.5281/zenodo.10699458)[13826820](https://zenodo.org/records/13826820) (Groussman et al., 2024a): processed nucleotide sequences and short read counts for ALOHA diel, G1-G3 surface, and G3 diel samples

Code used to generate the ALOHA diel assemblies, as well as the North Pacific Eukaryotic Gene Catalog (Groussman et al., 2024c): <https://github.com/armbrustlab/NPac_euk_gene_catalog>.

<https://doi.org/10.5281/zenodo.14519070> (Thomas et al., 2025a):

- G2 incubation chlorophyll a measurements
- G2 incubation short read counts
- G3 depth profile raw metatranscriptomic assemblies, processed and translated protein sequences, processed nucleotide sequences, and taxonomic and functional annotations
- ALOHA diel, G1-G3 surface, G3 diel, G2 incubations, and G3 depth profile TPM per sample, taxonomic bin, and annotated Pfam

G2 incubations and G3 depth read processing scripts, internal standards workflow, and G2 incubation and G3 depth standards normalization factors: <https://github.com/armbrustlab/MarPRISM/tree/main/processMarineMetatranscriptomes>.

Links to environmental data downloaded from Simons CMAP (<https://simonscmap.com>) can be found in Supplementary Data Sheet 7.

- This data originates from numerous publications (Ribalet et al., 2019; Juranek et al., 2020; Pinedo-González et al. 2020; Park et al., 2023; Hawco et al., 2025) and data repositories (Cain et al., 2020a; Cain et al., 2020b; Cain et al., 2020c; Juranek, 2020a; Juranek, 2020b; Juranek, 2020c; White, 2020; White, 2021; Simons CMAP Curator, 2022; NASA Goddard Space Flight Center, Ocean Ecology Laboratory, Ocean Biology Processing Group, 2022; Dave Karl Lab, 2023; John, 2023; Ribalet et al., 2024)

Gradients 1-3 dissolved iron concentrations measured on station not underway are from Hawco et al. (2025).
